# Morphological description, character conceptualization and the reconstruction of ancestral states exemplified by the evolution of arthropod hearts

**DOI:** 10.1371/journal.pone.0201702

**Published:** 2018-09-20

**Authors:** Torben Göpel, Christian S. Wirkner

**Affiliations:** Allgemeine & Spezielle Zoologie, Institut für Biowissenschaften, Universität Rostock, Rostock, Germany; PLOS, UNITED KINGDOM

## Abstract

Arthropods are the most species-rich taxon within Metazoa and have gone through major evolutionary changes with regard to body organization. Arthropod hearts and their associated vascular systems are thus morphologically highly disparate: while some arthropods exhibit very powerful hearts and complex vascular systems, other arthropods do not possess any kind of vascular system or heart at all. A comprehensive study investigating the structure of arthropods hearts has never been undertaken. In this study, we therefore investigate the hearts of 34 species from all major arthropod groups using various imaging techniques (confocal laser scanning microscopy, micro-computed tomography, histology) and describe them by addressing different aspects of heart morphology, e.g. the structure of the myocard or the composition of ostia. In a next step, we conceptualize 18 characters related to heart morphology and their respective character states and–using additional data from the literature–score a matrix for a total of 45 species from 38 supraspecific taxa. We map the characters onto prevailing phylogenetic hypotheses and perform parsimony-based ancestral state reconstruction to trace the evolutionary transformations undergone by arthropod hearts. An exploration of the character concepts (as explanatory hypotheses) reveals ontological peculiarities of character statements that clearly distinguish them in terms of ontological status from descriptive statements (i.e. descriptions of morphemes). The implications of these findings influence the interpretation of ground patterns as explanations. This first phylogenetic approach to heart morphology in the arthropod ground pattern reveals numerous new putative synapomorphies and leads to a reconsideration of the morphology of circulatory systems in early arthropods. Hypotheses on the evolution of hearts in (Pan-) Arthropoda are illustrated and discussed.

## Introduction

To misquote May [1], we would like to specify that:

“To a rough approximation, and setting aside vertebrate chauvinism, it can be said that essentially all organisms are [arthropods].”

The circulatory system in arthropods, descriptions of which have existed since the early days of sophisticated zoomorphology (e.g. [2, 3]), has been shown to be a highly disparate organ system [4] and a good object on which to study evolutionary transformations [5]. However, although arthropods are the most species-rich taxon on earth, their ecological, economic and evolutionary influence on the development and state of our planet is usually drastically underestimated. It is therefore hardly surprising that though the heart is thought to be homologous among arthropods, no comparative investigation of the four major arthropod groups (Chelicerata, Myriapoda, “Crustacea”, Hexapda) has ever been carried out. We thus aim to approach the “heart” from a descriptional, ontological and evolutionary perspective by providing morphological descriptions, discussing these in an ontological conceptual sense and finally conceptualizing phylogenetic characters and their respective character states. To take the evolutionary interpretation further, characters are traced over a recent phylogenetic hypothesis for arthropods.

In the era of digitalization, the field of morphology has experienced a renaissance, benefitting not only from new imaging technologies such as micro-computed tomography (e.g. [6, 7]) and confocal laser scanning microscopy (e.g. [8, 9]), but also from formalizations of terminology and descriptions [10–13] (first and foremost computer-parsable semantic representations of terms and their underlying concepts and definitions, i.e. ontologies; see e.g. [13–17]) which overcome the so called “linguistic problem of morphology” [14]. As ontologies are grounded in ontology, the field of philosophy which deals with being and reality [18, 19], exploration of the connection between the entities referred to by ontologies and the entities dealt with in morphology and/or systematics is a crucial step.

As the ultimate goal in science is to explain phenomena observed in nature, a sharp distinction has to be drawn between the *description* of observations and the *explanations* for them [20, 21]. In evolutionary morphology, this leads to a distinction between the description of *morphemes* [22] and explanatory hypotheses on phylogeny [23], which include the transformation of *characters*. The differences between *morphemes* (as units of description), *ontology concepts* (as classifications), and *characters*/*character states* (as units of evolution and thus explanation) have recently been pointed out on a theoretical level [22, 24] and confirmed by statistical analysis of the phenomic content of morphological descriptions and morphology-based character matrices [25].

The present study is intended to serve as case study, bringing together theoretical findings on the differences between morphemes, ontology concepts and characters/character states and demonstrating the consequences of their practical application. We investigated heart structure in 34 representatives of all major groups of arthropods and complemented our findings with further data from the literature. The goal was to reconstruct heart evolution within arthropods and to explore the consequences of theory on the process which begins with the description of hearts and extends to the conceptualization of characters and character states and ultimately to the derivation of hypotheses on the evolutionary transformations undergone by characters. Our overarching aim was to increase understanding of the evolution of morphological disparity. Arthropods are the most species-rich taxon on earth and exhibit a high degree of morphological disparity in both internal and external morphology. Our taxon sampling represents an attempt to cover the range of disparity in heart morphology in arthropods. As is known from the literature, insects are relatively uniform when it comes to heart morphology [26], despite being the group with the highest species diversity. Heart morphology in Malacostraca, however, is extremely varied (see e.g. [5, 27, 28]), which is why a denser taxon sampling was chosen here.

## Material and methods

### Investigated species

In this study, we investigated the heart morphology of 34 arthropod species and complemented our findings with data from the literature to obtain information on heart morphology for a total of 45 species from all four so called “main groups” of Arthropoda (Table 1).

**Table 1:** List of investigated species and literature data.

Taxon		Source
**Chelicerata**		
*Endeis* sp.	Pycnogonida	[29]
*Limulus polyphemus* (Linnaeus, 1758)	Xiphosura	this study; [30]
*Euscorpius tergestrinus* (Koch, 1837)	Euscorpiidae	this study
*Hottentotta hottentotta* (Fabricius, 1787)	Buthidae	this study
*Lasiodora parahybana* Mello-Leitão, 1917	Mygalomorphae	this study
*Araneus diadematus* (Clerck, 1757)	Araneomorphae	this study; [31]
**Myriapoda**		
*Scutigera coleoptrata* (Linnaeus, 1758)	Scutigeromorpha	this study; [32]
*Geophilus flavus* (De Geer, 1778)	Geophilomorpha	this study
*Lithobius forficatus* (Linnaeus, 1758)	Lithobiomorpha	this study; [32]
*Pauropus silvaticus* Tiegs, 1943	Pauropoda	[33]
*Glomeris marginata* (Villers, 1789)	Glomeridae	this study; [34]
*Polydesmus complanatus* (Linnaeus 1761)	Polidesmidae	this study; [34]
"Crustacea"*		
*Doloria levis* (Skogsberg, 1920)	Myodocopa	[35]
*Artemia franciscana* Kellogg, 1906	Anostraca	this study
*Triops cancriformis* (Bosc, 1801)	Notostraca	this study
*Lepidurus arcticus* (Pallas, 1793)	Notostraca	[36]
*Daphnia magna* Straus, 1820	Cladocera	this study
*Centropages typicus* Krøyer, 1849	Calanoida	this study
*Eurytemora affinis* (Poppe, 1880)	Calanoida	this study
*Pseudodiaptomus pelagicus* Herrick, 1884	Calanoida	this study
*Cyclops* sp.	Cyclopoida	[37]
*Munida sarsi* Huus, 1935	Anomura	this study; [28]
*Procambarus fallax* f. *virginalis* Martin et al., 2010	Astacidea	this study
*Pasiphaea multidentata* Esmark, 1866	Pasiphaeidae	this study
*Nebalia bipes* (O. Fabricius, 1780)	Leptostraca	this study
*Nebalia herbstii* Leach, 1814	Leptostraca	this study
*Gonodactylaceus falcatus* (Forskål, 1775)	Stomatopoda	this study
*Anaspides tasmaniae* (Thompson, 1893)	Anaspidacea	this study; [38]
*Meganyctiphanes norvegica* (M. Sars, 1857)	Euphausiacea	[27, 39]
*Asellus aquaticus* (Linnaeus, 1758)	Asellota	this study; [40]
*Leucon nasica* Krøyer, 1846	Cumacea	this study; [41]
*Diastyloides biplicatus* (G. O. Sars, 1865)	Cumacea	this study
*Diastylis tumida* (Liljeborg, 1855)	Cumacea	this study
*Hemilamprops uniplicatus* (G. O. Sars, 1872)	Cumacea	this study
*Lophogaster typicus* M. Sars, 1857	Lophogastrida	[42]
*Mictocaris halope* Bowman & Iliffe, 1985	Mictacea	[43]
*Neomysis integer* (Leach, 1814)	Mysida	[42]
*Spelaeogriphus lepidops* Gordon, 1958	Spelaeogriphacea	[43]
*Apseudes spinosus* (M. Sars, 1858)	Tanaidacea	this study
*Tethysbaena argentarii* (Stella, 1951)	Thermosbaenacea	[44]
*Hutchinsoniella macrantha* Sanders, 1955	Cephalocarida	this study; [45]
*Xibalbanus tulumensis* (Yager, 1987)	Remipedia	this study
**Hexapoda**		
*Petrobius brevistylis* Carpenter, 1913	Machilidae	this study; [46]
*Procloeon bifidum* (Bengtsson, 1912)	Ephemeroptera	this study
*Blaptica dubia* (Serville, 1838)	Blattodea	this study

*Although it would be better to use only monophlya to classify the studied species, “Crustacea” is kept here to uphold the four traditional major arthropod groups.

### Specimen preparation

Specimens used for micro-computed tomography (μCT) were fixed using Bouin’s fixative (for marine animals, the fixative was set to 30 PSU). Specimens were stained with Lugol’s solution (iodine potassium iodide in water) or 0.3% phosphotungstic acid in 70% ethanol. Some specimens were additionally either freeze-dried using a UniCryo MC2L (UniEquip, Munich, Germany), critical-point dried (Leica EM CPD00; Leica Microsystems, Wetzlar, Germany) or chemically dried using HMDS [47]. Specimens used for confocal laser scanning microscopy (cLSM) were fixed in 4% paraformaldehyde in 1x PBS for 2 to 12 hours and stained with a 1:100 dilution of Alexa Fluor 546 conjugated Phalloidin (Molecular Probes: catalog no. A22283) in PBT for 45 minutes to three hours. Specimens used for histology were treated as in [42].

### Micro-computed tomography (μCT)

Specimens were mounted onto a specimen holder. X-ray imaging was performed using either a phoenix nanotom (phoenix|x-ray, GE Measurement & Control, Wunstorf, Germany) using the program datos|x acquisition (target: Tungsten, mode: 0; performance: ca. 15–24 W; 1440 projections; detector timing: 500–1000 ms) or a XRadia Versa 410 x-ray microscope (ZEISS, Oberkochen, Germany) using the program Scout and Scan v.11 (40–60 kV; 150–200 μA; 2001–2701 projections; 5–20 s acquisition time).

### Fluorescence microscopy (fluo) and confocal laser scanning microscopy (cLSM)

Stained specimens were mounted in RapiClear 1.47 (SunJin Lab Co., Taiwan) in specimen chambers made of two coverslips and iSpacers (SunJin Lab Co., Taiwan) which were sealed using clear nail polish. Specimens were analyzed using a Leica DMI6000 CFS microscope equipped with a Leica TCS SP5 II confocal laser scanning unit (Leica Microsystems, Wetzlar, Germany). Image stacks of optical sections were recorded at a step size of 0.3–2 μm. For larger overview images, specimens were investigated using a Keyence Biozero BZ-8100 fluorescence microscope (Keyence Corporation, Osaka, Japan).

### Histology

Serial semi-thin sections were digitalized using an Axio Imager.M1 (ZEISS, Jena, Germany) equipped with a ZEISS AxioCam ICc 3 and the software AxioVs40 v.4.7.1.0, converted to 8-bit grayscale using IrfanView and aligned using Autoaligner x64 6.0 (Bitplane, Zurich, Switzerland).

### 3D reconstruction and image processing

Stacks of virtual sections from histology, μCT and cLSM were analysed as virtual volumes using the software package Imaris 7.0.0 (Bitplane, Zurich, Switzerland). In the program module “Surpass”, the volume was analyzed and desired parts of the volume were masked using isosurface reconstructions to then be displayed in separate channels. All figures were arranged and labelled using CorelDraw Graphics Suite X3 (Corel Corp., Ottawa, Canada).

### Reconstruction of ancestral character states and character transformation

To obtain useful coverage of the investigated taxa by a phylogenetic hypothesis, we constructed a composite cladogram using the phylogenetic hypotheses put forward by Regier et al. [48], Wirkner & Richter [5] and Schwentner et al. [49]. We chose a supertree approach using matrix representation with parsimony (MRP) in the Baum Ragan coding scheme [50]. The MRP matrix was constructed using the software SuperTree [51] and was then analyzed using TNT (WHS edition, [52]). The single most parsimonious composite cladogram covers all the supraspecific taxa represented by the species considered in this study. We conceptualized 18 characters (all unordered) relating to heart morphology and scored a character matrix for the 45 species considered in Table 1. To obtain correspondence between matrix and cladogram, we concatenated the species-based character matrix to a matrix consisting of 38 supraspecific taxa. Ancestral states and character transformations were reconstructed under parsimony using the software package Mesquite 3.2 (build 801) by Maddison & Maddison [53] with the functions “Trace Character History” and “Trace All Characters”. Reconstructions of all characters (including ambiguities) are provided in the supplementary information (S1 File). For the exposition of hypotheses on character evolution provided in this manuscript, we chose from among the most parsimonious reconstructions (MPRs mode in Mesquite) and provide our arguments in favor of each decision.

## Results

### Descriptions of heart morphology

The descriptions provided here all use natural language. However, to ensure intersubjective comprehensibility, all terms referring to parts of the circulatory system are taken from OArCS, the Ontology of Arthropod Circulatory Systems [13], which is also available via http://oarcs.speciesfilegroup.org. All descriptions are structured in the same way, based on the five categories of the semantic model for the description of morphemes provided by Wirkner et al. [13].

#### Chelicerata: Xiphosura: *Limulus polyphemus*

**Fig 1:**
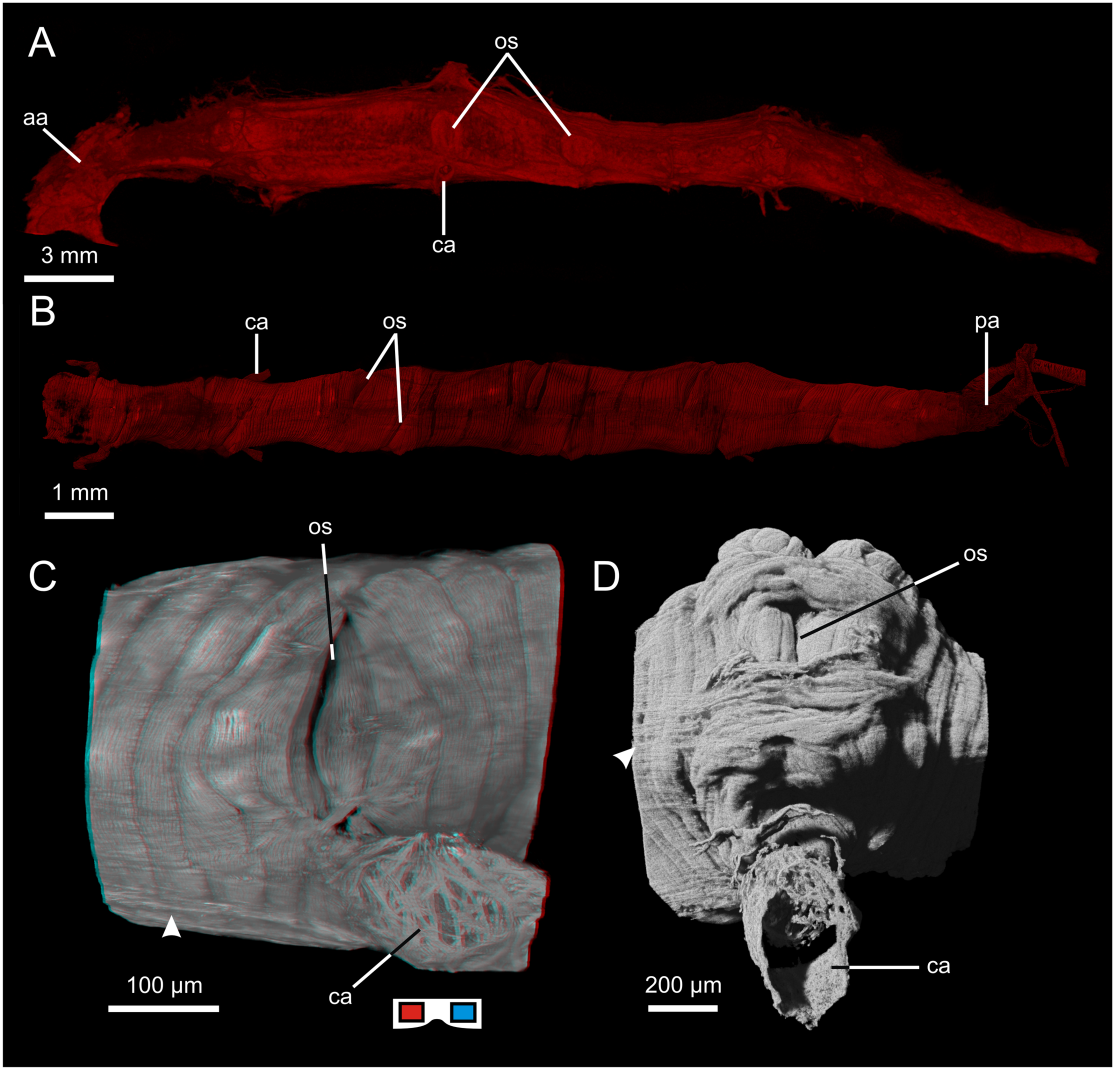
Hearts of chelicerates. A: Heart of *Limulus polyphemus* (lateral view, μCT). B: Heart of *Hottentotta hottentotta* (dorsal view, cLSM). C: Ostium and proximal part of cardiac artery of *Euscorpius tergestrinus* (lateral view, cLSM; red cyan anaglyph, use goggles), arrowhead indicates longitudinal fibers of the epicard. D: Ostium and proximal part of cardiac artery of *Lasiodora parahybana* (lateral view, cLSM), arrowhead indicates longitudinal fibers of the epicard. aa: anterior aorta; ca: cardiac artery; os: ostium; pa: posterior aorta.

The heart lies in the dorsal median line and extends from the middle of the prosoma to the middle of the opisthosoma. The heart is fusiform (Fig 1A) and largest in diameter in the anterior opisthosoma. The transversal section of the heart is round in shape in the anterior part, triangular in the middle part and describes a dorsoventrally flattened ellipsoid in the posterior part. The heart consists of an outer epicard with mostly longitudinal elastic fibers and an inner myocard made up of a meshwork of branching cardiomyocytes which run almost exclusively in the transversal plane (see also [30]). Eight pairs of ostia are situated dorsolaterally (Fig 1A) and are segmentally arranged. The ostia are shallow and consist of paired flap-like valves. An anterior aorta emanates at the anterior apex of the heart. At its origin, a single flap-like aortic valve prevents backflow of hemolymph. Four pairs of cardiac arteries emanate ventrolaterally from the prosomal part of the heart (ventrally to the first four pairs of ostia). Posteriorly, the heart ends blindly.

#### Chelicerata: Scorpiones: Buthidae: *Hottentotta hottentotta*

The heart lies in the dorsal median line, extends from the anterior end of the mesosoma to the anterior region of the last mesosomal segment and is fusiform in shape (Fig 1B). The heart consists of an outer epicard which takes the form of a thin sheath of longitudinal connective tissue fibers and an inner myocard which is a single layer of cardiomyocytes shaped like semicircular arches. The cardiomyocytes are contralaterally (cell junctions in the dorsal and ventral median line) but helically arranged, i.e. the cardiomyocytes which connect dorsally do not connect ventrally. The helical shift is by 15 to 20 cardiomyocytes per 360 °. Six pairs of slit-like ostia are situated dorsolaterally and are segmentally arranged. The contralateral ostia do not lie directly opposite each other but are diagonally shifted (Fig 1B). Seen from the inside, the muscular lips protrude deep into the heart lumen. Anteriorly, an anterior aorta emanates from the heart, exhibiting at its origin a semilunar aortic valve which is suspended dorsally. Eight pairs of cardiac arteries emanate ventrolaterally from the heart and are separated from the heart by muscular arterial valves. The first pair emanates close to the anterior apex of the heart, the following five pairs of cardiac arteries emanate directly beneath the first five pairs of ostia. The last two emanate in close vicinity to each other close to the posterior apex of the heart, where a posterior aorta emanates which is separated from the heart by a muscular aortic valve.

#### Chelicerata: Scorpiones: Euscorpiidae: *Euscorpius tergestrinus*

The heart lies in the dorsal median line, extends throughout the mesosoma and is fusiform in shape. The heart consists of an outer epicard which takes the form of a thin sheath of longitudinal connective tissue fibers (Fig 1C) and an inner myocard which is a single layer of cardiomyocytes shaped like semicircular arches. The cardiomyocytes are contralaterally (cell junctions in the dorsal and ventral median line) but helically arranged, i.e. the cardiomyocytes which connect dorsally do not connect ventrally. The helical shift is by three to six cardiomyocytes per 360 °. Seven pairs of slit-like ostia (Fig 1C) are situated dorsolaterally and are segmentally arranged. The left and right ostium lie directly opposite each other. Seen from the outside, the ostia appear as mere slits (Fig 1C). Seen from the inside, the muscular lips protrude deep into the heart lumen. Anteriorly, an anterior aorta emanates from the heart. At its origin a semilunar aortic valve is suspended dorsally. Six pairs of cardiac arteries (Fig 1C) emanate ventrolaterally from the heart. Posteriorly, a posterior aorta emanates from the heart.

#### Chelicerata: Araneae: Mygalomorphae: *Lasiodora parahybana*

The heart lies in the dorsal median line of the opisthosoma and extends from the pedicel to the posterior end of the opisthosoma. The heart is fusiform and runs just beneath the dorsal cuticle. The heart consists of a thin outer epicard (Fig 1D) made up of longitudinal fibers and a single layered myocard made up of cardiomyocytes arranged in parallel. The cardiomyocytes are shaped like semicircular arches, the cell junctions lie in the dorsal and ventral median line. Three pairs of ostia are situated dorsolaterally in the heart. The 1^st^ is just behind the anterior apex, the 2^nd^ at about one third of the length and the 3^rd^ at about two thirds of the length of the heart. The size of the ostia increases from anterior to posterior. Seen from the outside, the ostia appear as mere slits (Fig 1D), but muscular lips protrude deep into the lumen. Three pairs of cardiac arteries (Fig 1D) emanate ventrolaterally directly beneath the three pairs of ostia. At the origin of each cardiac artery, an arterial valve in the form of a muscular cuff protrudes into the proximal part of the arterial lumen.

#### Chelicerata: Araneae: Araneomorphae: *Araneus diadematus*

The heart lies in the dorsal median line of the opisthosoma and extends from the pedicel to the mid-posterior region of the opisthosoma. The heart is fusiform and consists of an outer epicard made up of longitudinal fibers of connective tissue and an inner, single-layer myocard made up of cardiomyocytes arranged in parallel. The cardiomyocytes are shaped like semicircular arches with their junctions in the dorsal and ventral median line. Three pairs of ostia are situated dorsolaterally at equal intervals along the length of the heart. Seen from the outside, the ostia appear as mere slits, but muscular lips protrude deep into the lumen. Anteriorly, an anterior aorta emanates from the heart and is separated from the heart by a semilunar aortic valve which is suspended dorsally. Three pairs of cardiac arteries emanate from the heart ventrolaterally: the 1^st^ beneath the 2^nd^ pair of ostia, the 2^nd^ beneath the 3^rd^ pair of ostia and the 3^rd^ close to the posterior apex of the heart. At the origin of each cardiac artery, an arterial valve in the form of a muscular cuff protrudes into the proximal part of the arterial lumen. Posteriorly, a posterior aorta emanates from the heart and is separated from the heart by a vertically orientated aortic valve with paired muscular lips.

#### Myriapoda: Chilopoda: Scutigeromorpha: *Scutigera coleoptrata*

**Fig 2:**
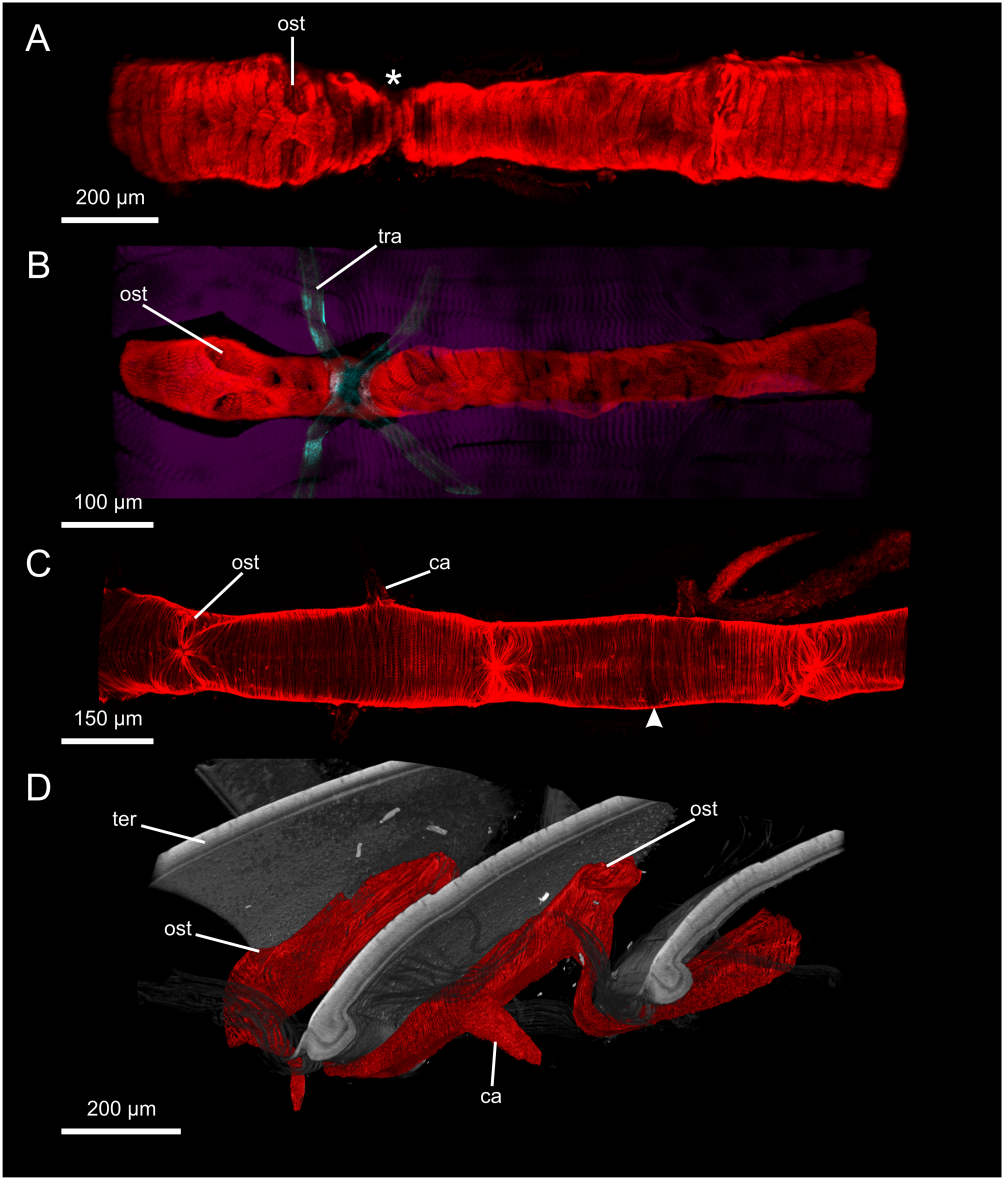
Hearts of myriapods. A: Part of the heart of *Scutigera coleoptrata* (dorsal view, cLSM), asterisk indicates narrowed region under the overlap of two consecutive tergites. B: Part of the heart of *Geophilus flavus* (dorsal view, cLSM) between the longitudinal trunk musculature (purple), tracheae are located near the ostia. C: Part of the heart of *Polydesmus complanatus* (dorsal view, cLSM), arrowhead indicates the origin of a cardiac artery which was ripped off during preparation. D: Part of the heart of *Glomeris marginata* (lateral view, μCT) in an unrolled specimen. ca: cardiac artery; ost: ostium; ter: tergite; tra: trachea.

The heart (Fig 2A) lies in the dorsal median line right beneath the dorsal integument. It is tube-shaped and extends from the height of the maxilliped through the trunk to about the middle of the distance between the two last pairs of walking legs. The heart consists of a single-layer myocard made up of cardiomyocytes arranged in parallel and shaped like semicircular arches. Cell junctions are situated in the dorsal and ventral median line. In horizontal sections of the heart, the cardiomyocytes have a cone-like profile with the tips protruding relatively deep into the lumen. The heart becomes thinner at the regions where it curves underneath the borders between adjacent tergites (Fig 2A, asterisk). Ostia lie dorsolaterally and are distributed segmentally. Seen from the outside, the ostia appear as mere slits (Fig 2A), the ostial valves protrude into the lumen. Anteriorly, an anterior aorta emanates from the heart and is separated from the heart via an aortic valve with paired lips which are suspended laterally. Cardiac arteries emanate segmentally ventrolaterally from the heart right beneath the ostia. The last pair of cardiac arteries emanates ventrally close to the posterior apex of the heart, which ends blindly, and the two contralateral arteries are fused at their origin.

#### Myriapoda: Chilopoda: Geophilomorpha: *Geophilus flavus*

The heart (Fig 2B) lies in the dorsal median line and extends from the maxilliped segment to the last leg-bearing segment. The heart is tube-shaped and the myocard consists of a single layer of broad flat cardiomyocytes which are arranged in parallel and shaped like semicircular arches. The cell junctions are situated in the dorsal and ventral median line. In each segment, one pair of dorsolaterally situated ostia (Fig 2B) occurs. Seen from the outside, the ostia appear as mere slits, seen from the inside, the paired ostial valves protrude into the lumen. The opening of the ostia points posteriorly on both sides. Anteriorly, an anterior aorta emanates from the heart. As far as could be seen, one single pair of cardiac arteries, i.e. the maxilliped arch, emanates ventrolaterally from the anterior apex of the heart. Posteriorly, the heart ends blindly.

#### Myriapoda: Chilopoda: Lithobiomorpha: *Lithobius forficatus*

The heart lies in the dorsal median line and extends from the maxilliped segment to the 15^th^ segment (last walking leg-bearing segment). The heart is tube-shaped and the myocard consists of a single layer of broad flat cardiomyocytes which are arranged in parallel and shaped like semicircular arches. The cell junctions are situated in the dorsal and ventral median line. In each segment, one pair of dorsolaterally situated ostia occurs. Seen from the outside, the ostia appear as mere slits, seen from the inside, the paired ostial valves protrude into the lumen. The heart narrows posteriorly to each pair of ostia and the ostial opening points posteriorly. Anteriorly, an anterior aorta emanates from the heart and is separated from the heart by an aortic valve which is vertically orientated and consists of paired lateral lips. Three pairs of cardiac arteries emanate from the heart ventrolaterally. The first pair, i.e. the maxilliped arch, emanates close to the anterior apex of the heart in the maxillipedal segment, the second and third pair from the eleventh and twelfth segments respectively. Posteriorly, the heart ends blindly.

#### Myriapoda: Diplopoda: Polydesmidae: *Polydesmus complanatus*

The heart (Fig 2C) lies in the dorsal median line and extends throughout the trunk. The heart is tube-shaped and consists of a single-layer myocard whose narrow cardiomyocytes are arranged in parallel and shaped like semicircular arches. The cell junctions lie in the dorsal and ventral median line. There are two pairs of dorsolaterally situated ostia (Fig 2C) within each diplosegment. Seen from the outside, the ostia appear (more or less) slit-like and have paired valves protruding into the lumen. Anteriorly, an anterior aorta emanates from the heart. In each diplosegment, two pairs of cardiac arteries emanate ventrolaterally from the heart. The cardiac arteries emanate halfway between two consecutive pairs of ostia (Fig 2C).

#### Myriapoda: Diplopoda: Glomeridae: *Glomeris marginata*

The heart (Fig 2D) lies in the dorsal median line and extends from the collum to the last diplosegment. The heart is tube-shaped and consists of a single-layer myocard. The narrow cardiomyocytes are arranged in parallel, and shaped like semicircular arches. The cell junctions lie in the dorsal and ventral median line. There is extensive attachment between the heart and the dorsal integument. When the animal is straightened, the heart displays strong folds where the tergites overlap, scale-like (Fig 2D). This means that when the animal is rolled, the heart can follow the extended dorsal median line without being ripped apart. There are two pairs of dorsolaterally situated ostia within each diplosegment. The anterior pair is situated at about one third of the length of the diplosegment, the posterior pair at about two thirds. In straightened animals, the posterior pair of ostia is situated at the dorsalmost part of the fold. Seen from the outside, the ostia appear as mere slits and have paired valves protruding into the heart lumen. Anteriorly, an anterior aorta emanates from the heart. In each diplosegment (as far as we could see), one pair of cardiac arteries emanates ventrolaterally from the heart beneath the anterior pair of ostia and is separated from the heart by an arterial valve with paired lips which is orientated horizontally. However, Leiber [34] describes two pairs of cardiac arteries per diplosegment.

#### “Crustacea”: Branchiopoda: Anostraca: *Artemia franciscana*

Image data for *A*. *franciscana* in this study is only available for some regions of the heart, meaning that an exhaustive description cannot be given. For a description of the heart morphology in *A*. *salina* see Vehstedt [54] and Økland et al. [55]. Our own results show that in *A*. *franciscana*, the cardiomyocytes are arranged in parallel and shaped like semicircular arches. The ostia are made up of paired valves which form a V-shaped trench.

#### “Crustacea”: Branchiopoda: Notostraca: *Triops cancriformis*

The heart lies in the dorsal median line and extends through the first eleven thoracic segments. The heart is fusiform and consists of a single-layer myocard made up of cardiomyocytes arranged in parallel and shaped like semicircular arches. Eleven pairs of ostia are situated laterally; one pair at the end of each segment. The ostia consist of paired muscular valves which form a V-shaped trench.

#### “Crustacea”: Branchiopoda: Cladocera: *Daphnia magna*

**Fig 3:**
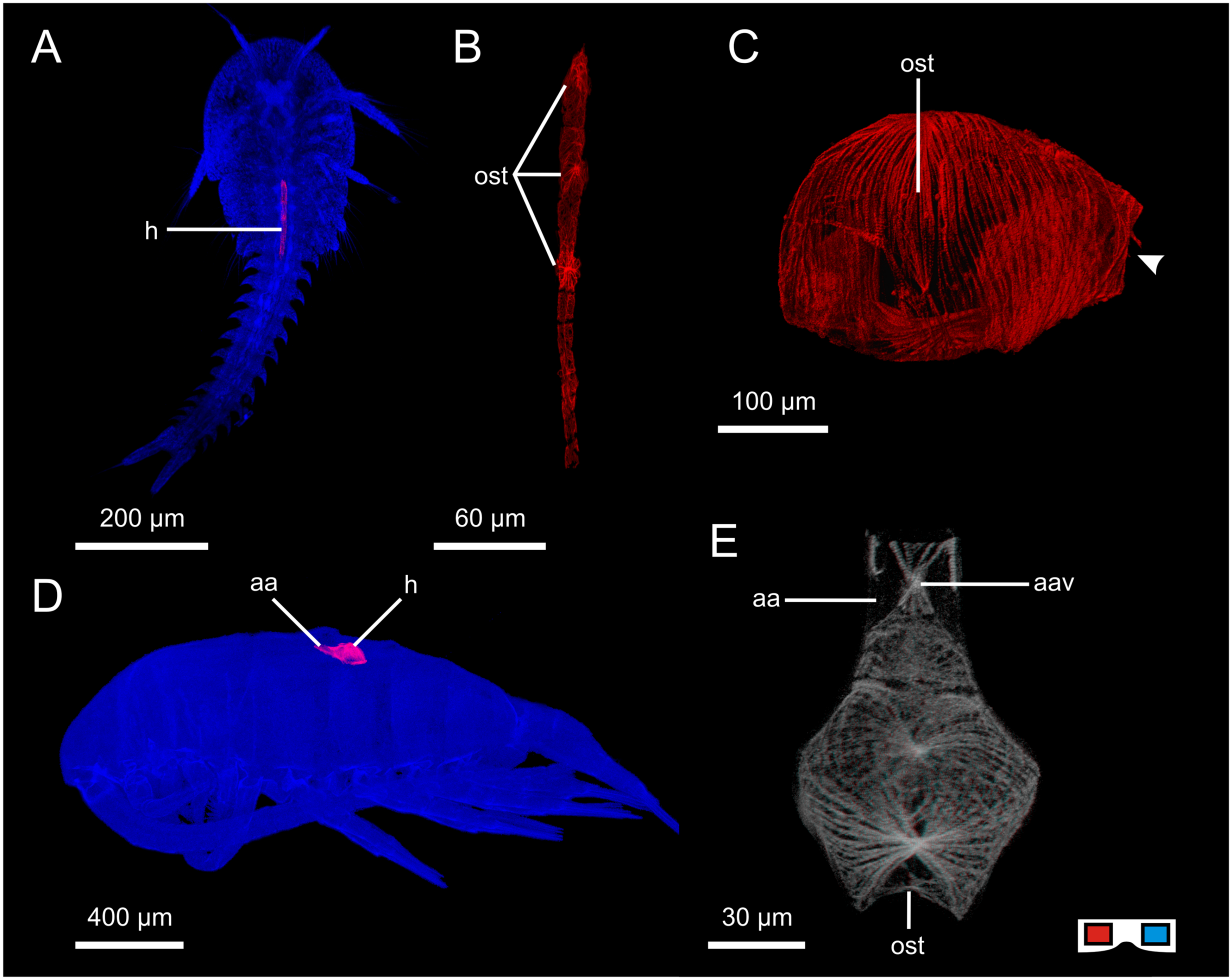
Hearts of some non-malacostracan crustaceans. A: Heart of *Hutchinsoniella macracantha*, location in the body (ventral view, cLSM). B: Heart of *H*. *macracantha* (dorsal view, cLSM). C: Heart of *Daphnia magna* (lateral view, anterior is right, cLSM), arrowhead indicates anterior opening of the heart. D: Heart of *Pseudodiaptomus pelagicus*, location in the body (lateral view, cLSM). E: Heart of *Centropages typicus* (dorsal view, cLSM, red cyan anaglyph, use goggles). aa: anterior aorta; aav: anterior aortic valve; h: heart; ost: ostium.

The heart lies dorsomedially in the anterior part of the body directly behind the compound eye. The heart is roundish/sack-like (Fig 3C) and consists of a single-layer myocard. The cardiomyocytes span meridionally from a crest in the dorsal median line to a ventral crest. One pair of ostia (Fig 3C, ost) is situated laterally. Each ostium consists of paired valves which form a V-shaped trench.

#### “Crustacea”: Copepoda: Calanoida: *Centropages typicus*; *Eurytemora affinis*; *Pseudodiaptomus pelagicus*

As the heart morphology of these three species does not differ at the given level of granularity it is described for all three species as one.

The heart lies dorsomedially in the thorax (Fig 3D). The heart is roundish/sack-like with a pointed anterior apex. The myocard consists of a single layer of cardiomyocytes which span meridionally from a dorsal crest to a ventral crest (Fig 3E). The myofibers branch frequently between the two crests. There is one large unpaired ostium at the posterior apex situated directly beneath the dorsal crest. The ostial opening forms a V-shaped trench. An anterior aorta emanates from the anterior apex of the heart and is separated from the heart by an aortic valve with paired lips which are suspended laterally (Fig 3E, aav).

#### “Crustacea”: Malacostraca: Leptostraca: *Nebalia herbstii*; *N*. *bipes*

As the heart morphology of these two species does not differ at the given level of granularity, it is described for both species as one.

**Fig 4:**
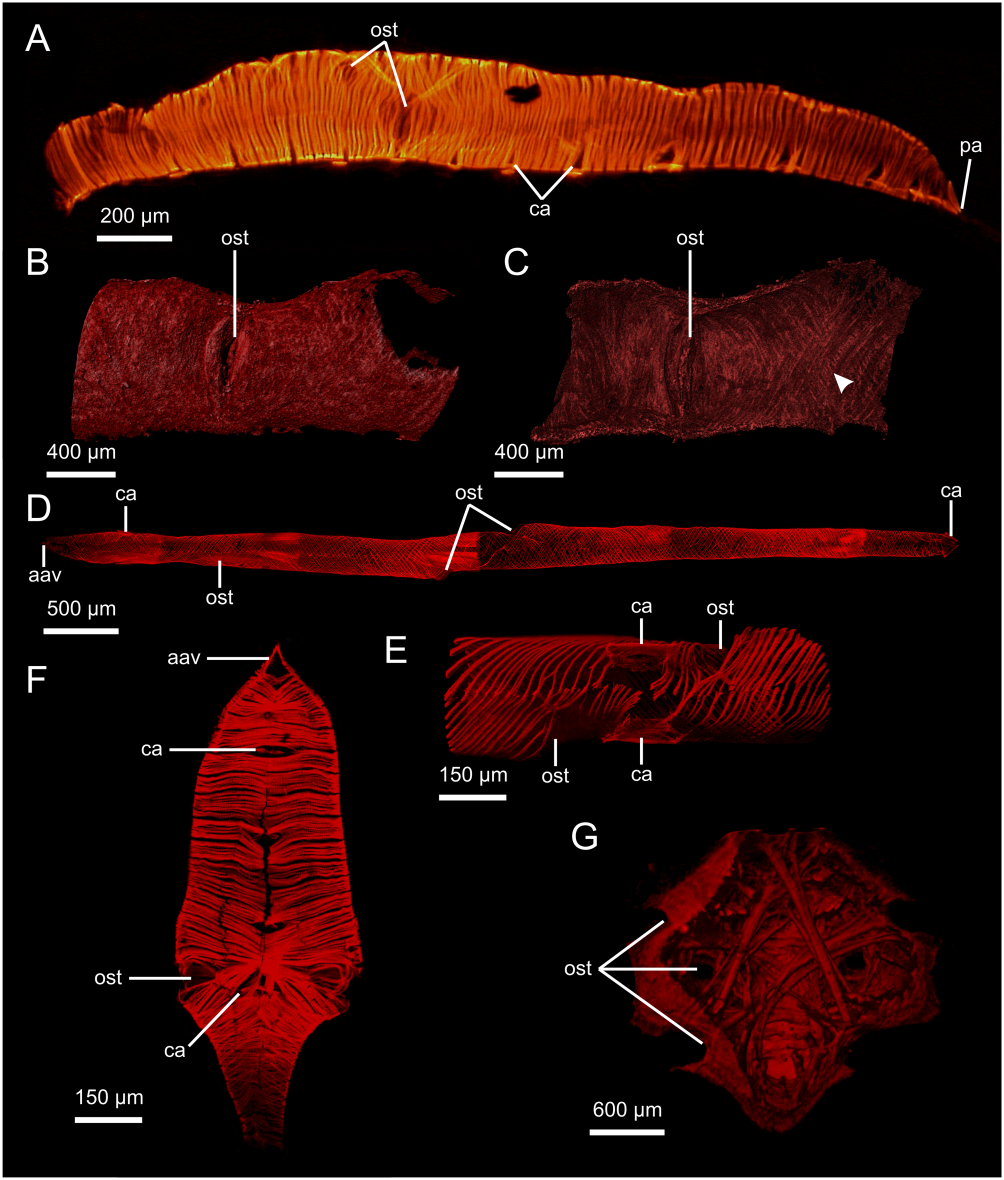
Hearts of malacostracan crustaceans. Heart of *Nebalia herbstii* (lateral view, fluo). B, C: Myocard and ostium of *Anaspides tasmaniae* (B: lateral view of exterior, C: interior, μCT), arrowhead indicates double layer of myofibers running in opposite directions. D: Heart of *Apseudes spinosus* (ventral view, cLSM). E: Ostia and origins of cardiac arteries of *A*. *spinosus* (ventral view, cLSM). F: Heart of *Neomysis integer* (ventral view, cLSM). G: Heart of *Munida sarsi* (dorsal view, μCT), dorsal surface virtually clipped to show bundles of myofibers running through the heart lumen. aav: anterior aortic valve; ca: cardiac artery; ost: ostium; pa: posterior aorta.

The heart lies in the dorsal median line and extends from the posterior margin of the head to the fourth pleomere. The heart is tube-shaped (Fig 4A) and ellipsoid in cross-section. The heart consists of an outer epicard and an inner myocard. The myocard is a single layer of cardiomyocytes shaped like semicircular arches which are arranged in parallel. The cell junctions are situated in the dorsal and ventral median line. On the inside, the heart displays eight longitudinal rows of non-muscular pouches. There are six pairs of ostia all situated in the anterior half of the heart. The first two pairs of ostia are situated laterally, the third to fifth pairs are situated dorsolaterally and the sixth pair again laterally. The sixth pair of ostia is significantly larger than the others (Fig 4A). Each ostium is shallow, has a vertically orientated slit and consists of paired valves. The two valves are dorsally and ventrally attached to cardiomyocyte processes which bend around the ostium. The dorsolateral ostia almost touch each other; in the fourth and fifth pair, the two ostia on each side share a dorsal knot. The sixth ostium, which is larger, also differs in that the dorsal knots of this pair are attached to the dorsomedian portion of the heart via three or four large cardiomyocytes which run anteriorly from the right ostium but posteriorly from the left ostium. The ventral knots of the sixth ostia are directly connected to each other via thick cardiomyocytes.

#### “Crustacea”: Malacostraca: Stomatopoda: *Gonodactylaceus falcatus*

The heart lies in the dorsal median line and extends from the first maxilliped segment to the posterior end of the fifth pleomere. The heart is tube-shaped and appears ellipsoid in cross-section. The myocard consists of a single layer of cardiomyocytes which are arranged helically. The cardiomyocytes are shaped like semicircular arches with their junctions in the dorsal and ventral median line. There are 13 pairs of diagonally tilted ostia which are situated dorsolaterally. The first three pairs are found in the maxillipedal region and are unevenly distributed: the second and third pair are found in close vicinity to each other while the distance between the first and the second pair is markedly larger. The fourth to twelfth pairs are situated at the posterior end of each of the post-maxillipedal segments. The 13^th^ pair of ostia is significantly smaller than the others and situated close to the posterior apex of the heart. Anteriorly, an anterior aorta emanates from the heart and is separated from the heart by an aortic valve which is orientated vertically and consists of paired lateral lips. Posteriorly, a posterior aorta emanates from the heart and is separated from the heart by an aortic valve which is orientated vertically and consists of paired lateral lips. 14 pairs of cardiac arteries emanate from the heart ventrolaterally, most of which originate ventrally to a pair of ostia. The first pair emanates slightly anterior to the first pair of ostia. The second and third pair of cardiac arteries emanate between the first and second pair of ostia. The remaining eleven pairs of cardiac arteries emanate ventrally to the second to twelfth pairs of ostia. Each cardiac artery is separated from the heart by a horizontally orientated arterial valve with paired lips.

#### “Crustacea”: Malacostraca: Decapoda: Caridea: *Pasiphaea multidentata*

The heart lies in the posterodorsomedian portion of the cephalothorax. The heart is roundish in its overall geometry and is roughly trapezoid in horizontal section and roughly pentagonal in transverse section. The myocard is a dense compound of irregularly arranged cardiomyocytes. Bundles of cardiomyocytes run across the lumen in various directions. There are five pairs of ostia (the first three pairs lie on the dorsal surface of the heart and are arranged in a V-shape): the first pair is situated almost at the lateral margin, the second pair halfway between median line and the lateral margin, the third pair close to the median line, the fourth pair on the ventral surface of the heart posteriorly (close to the lateral margin), and the fifth at the posterolateral edges of the heart. The ostia are shallow and have a pair of valves each. An anterior aorta emanates from the anterior apex of the heart and is separated from the heart by an aortic valve which is orientated vertically and has paired lips. A posterior aorta emanates from the posterior apex of the heart and is separated from the heart by an aortic valve which is orientated horizontally and has paired lips. Four cardiac arteries leave the heart, two of which are unpaired. Anteriorly, beneath the origin of the anterior aorta, a pair of cardiac arteries emanates and runs ventrally. Posteroventrally, two unpaired arteries, the origins of which lie in the in the median line, emanate from the heart. The anterior unpaired cardiac artery is slightly smaller than the posterior unpaired cardiac artery, i.e. the descending artery, which has a larger diameter at its origin. The cardiac arteries are separated from the heart by an arterial valve which is orientated perpendicular to the sagittal plane and consists of paired lips.

#### “Crustacea”: Malacostraca: Decapoda: Astacidea: *Procambarus fallax* f. *virginalis*

The heart lies in the posterodorsomedian portion of the cephalothorax. The heart is roundish in its overall geometry and is roughly pentagonal in horizontal and transversal section. The heart consists of an outer epicard and an inner myocard. The myocard is a dense compound of irregularly arranged cardiomyocytes. Bundles of cardiomyocytes run across the lumen in various directions. There are three pairs of ostia: the first pair is situated anterodorsolaterally, the second laterally and the third ventrolaterally. The ostia are flat and consist of paired valves. The ostial slits are orientated horizontally. An anterior aorta emanates from the anterior apex of the heart and is separated from the heart by an aortic valve made up of one pair of laterally suspended lips. Posteriorly, the aortic bulb (i.e. the enlarged proximal part of the posterior aorta) emanates from the posteroventral apex of the heart and is separated from the heart by an aortic valve which is orientated horizontally and has paired lips. From the aortic bulb, the descending artery emanates ventrally. Two pairs of cardiac arteries emanate from the anterior region of the heart. The anterior lateral arteries emanate directly beside the anterior aorta. The hepatic arteries emanate from the anteroventral portion of the heart. Both these paired cardiac arteries are separated from the heart by vertically orientated arterial valves with paired lips.

#### “Crustacea”: Malacostraca: Decapoda: Anomura: *Munida sarsi*

The heart lies in the posterodorsomedian portion of the cephalothorax. The heart is roundish in its overall geometry and is roughly pentagonal in horizontal and transversal section (Fig 4G). The heart consists of an outer epicard and an inner myocard. The myocard is a dense compound of irregularly arranged cardiomyocytes. Bundles of cardiomyocytes run across the heart lumen in various directions (Fig 4G). There are three pairs of ostia: the first pair is situated dorsolaterally, the second laterally and the third ventrolaterally. The ostia are flat and consist of paired valves. An anterior aorta emanates from the anterior apex of the heart and is separated from the heart by an aortic valve made up of one pair of laterally suspended lips. A posterior aorta emanates from the posteroventral apex of the heart. Two pairs of cardiac arteries and one unpaired cardiac artery emanate from the heart: the paired anterior-lateral arteries emanate anteriorly close to the origin of the anterior aorta. The paired hepatic arteries emanate anteroventrally from the heart. The unpaired descending artery emanates ventrally from the posteroventral apex of the heart, close to the origin of the posterior aorta.

#### “Crustacea”: Malacostraca: Anaspidacea: *Anaspides tasmaniae*

The overall morphology of the heart of *Anaspides tasmaniae* has been described by Siewing [38]. In this study, investigations were limited to the morphology of the myocard in terms of fiber arrangement. The myocard consists of two layers of helically arranged fibers which run in opposite directions (Fig 4B and 4C). The ostia are flat, consist of paired valves and the ostial slit is orientated vertically.

#### “Crustacea”: Malacostraca: Peracarida: Mysida: *Neomysis integer*

The heart lies in the dorsal median line. It is fusiform (Fig 4F) and extends throughout the thorax. The heart consists of an outer epicard and an inner myocard. The myocard consists of a single layer of cardiomyocytes which are arranged in parallel. The cardiomyocytes are shaped like semicircular arches and the cell junctions are situated in the dorsal and ventral median line. There are two pairs of ostia, both at about half the length of the heart, situated in close vicinity to each other. The anterior ostium lies anterodorsally of the second one. The ostia are flat and each ostium consists of paired muscular valves which do not protrude into the lumen. Anteriorly, an anterior aorta emanates from the heart and is separated from the heart by a vertically orientated aortic valve with paired lateral lips. Posteriorly, a posterior aorta emanates from the heart. Nine cardiac arteries, i.e. four pairs and an unpaired one, emanate from the ventral median line of the heart (Fig 4F). The four pairs of cardiac arteries emanate from the anterior half of the heart: the first pair close to the anterior apex, the fourth pair between the two pairs of ostia. The contralateral arteries lie directly adjacent to each other (some have been described previously as being unpaired; see [42]) and are separated from the heart by arterial valves with paired muscular lips, the distinctness of which clearly identifies these arteries as being paired. The ninth cardiac artery is the unpaired descending artery which emanates ventrally from the posterior part of the heart.

#### “Crustacea”: Malacostraca: Peracarida: Tanaidacea: *Apseudes spinosus*

The heart lies in the dorsal median line and extends from the third to the eighth thoracomere. The heart is tube-shaped and the myocard consists of a single layer of cardiomyocytes which are arranged helically (Fig 4D). The cardiomyocytes are shaped like semicircular arches with their junctions in the dorsal and ventral median line. There are three ostia: one unpaired ostium is situated laterally on the right side at about one fourth of the length of the heart from anterior and one pair of ostia is situated laterally at about half the length of the heart (Fig 4E). The unpaired ostium is significantly smaller than the paired ostia. The ostia are flat and each ostium consists of paired muscular valves which do not protrude into the lumen. The ostial opening of the paired ostia points in an anterior direction on the left side and in a posterior direction on the right side (Fig 4E). The paired ostia are not situated directly opposite each other—the right is more anterior than the left. Anteriorly, an anterior aorta emanates from the heart and is separated from the heart by a vertically orientated aortic valve with paired lateral lips. Three pairs of cardiac arteries emanate ventrolaterally from the heart and are separated from the heart by a horizontally orientated valve with paired lips. The first pair emanates just behind the anterior apex. The second pair emanates between the paired ostia. The third pair emanates at the posterior apex of the heart.

#### “Crustacea”: Malacostraca: Peracarida: Cumacea: *Leucon nasica*

The heart is fusiform and lies in the dorsal median line. It extends from around the middle of the cephalothorax to the sixth thoracomere. The heart consists of an outer epicard and an inner myocard. The myocard consists of a single layer of cardiomyocytes which are arranged in parallel. The cardiomyocytes are shaped like semicircular arches and only in the ostial region do they lose their parallel orientation and run rather irregularly (though predominantly diagonally), fusing in a ventral knot. The single pair of ostia is located laterally at about one third of the length of the heart (seen from anterior). The ostia consist of paired valves which form a V-shaped trench. The origins of the ostia are extruded. Thus, despite forming a V-shaped trench, the ostial valves do not protrude deeply into the lumen of the heart. Each ostium is suspended in the myocard by two prominent bundles of fibers which run almost longitudinally. Anteriorly, an anterior aorta emanates from the heart and is separated from the heart by a vertically orientated aortic valve with paired lateral lips. Posteriorly, the heart ends blindly. Five pairs of cardiac arteries emanate ventrolaterally from the heart and are each separated from the heart by a horizontally orientated valve with paired lips. The first pair of cardiac arteries emanates at about two thirds of the length of the heart (seen from anterior), the other four pairs emanate in close vicinity to each other in the posterior portion of the heart.

#### “Crustacea”: Malacostraca: Peracarida: Cumacea: *Diastyloides biplicatus*

The heart is fusiform and lies in the dorsal median line. The myocard consists of a single layer of cardiomyocytes which are arranged in parallel. The cardiomyocytes are shaped like semicircular arches and only in the ostial region do they lose their parallel orientation. The single pair of ostia is located laterally at about half the length of the heart. The ostia consist of paired valves which form a V-shaped trench. The origins of the ostia are extruded. Thus, despite forming a V-shaped trench, the ostial valves do not protrude deeply into the lumen of the heart. Each ostium is suspended in the myocard by two prominent bundles of fibers which run almost longitudinally. Anteriorly, an anterior aorta emanates from the heart and is separated from the heart by a vertically orientated aortic valve with paired lateral lips. Posteriorly, the heart ends blindly. At least four (maybe five, we were unable to tell with certainty) pairs of cardiac arteries emanate ventrolaterally from the heart, each of which is separated from the heart by an arterial valve which is horizontally orientated and has paired lips. The first pair of cardiac arteries emanates directly posterior to the ostia, the others emanate in close vicinity to each other at the posterior end of the heart.

#### “Crustacea”: Malacostraca: Peracarida: Cumacea: *Diastylis tumida*

The heart is fusiform and lies in the dorsal median line. The myocard consists of a single layer of cardiomyocytes which are arranged in parallel. The cardiomyocytes are shaped like semicircular arches and only in the ostial region do they lose their parallel orientation. The single pair of ostia is located laterally at about half the length of the heart. The ostia consist of paired valves which form a V-shaped trench. The origins of the ostia are extruded. Thus, despite forming a V-shaped trench, the ostial valves do not protrude deeply into the lumen of the heart. Each ostium is suspended in the myocard by two prominent bundles of fibers which run almost longitudinally. Anteriorly, an anterior aorta emanates from the heart and is separated from the heart by a vertically orientated aortic valve with paired lateral lips. Posteriorly, the heart ends blindly.

#### “Crustacea”: Malacostraca: Peracarida: Cumacea: *Hemilamprops uniplicatus*

The heart is fusiform and lies in the dorsal median line. It extends from around the middle of the cephalothorax to the seventh thoracomere. The heart consists of an outer epicard and an inner myocard. The myocard consists of a single layer of cardiomyocytes which are arranged in parallel. The cardiomyocytes are shaped like semicircular arches and only in the ostial region do they lose their parallel orientation and run rather irregularly (though predominantly diagonally), fusing in a ventral knot. The single pair of ostia is located laterally at about half the length of the heart. The ostia consist of paired valves which form a V-shaped trench. The origins of the ostia are extruded. Thus, despite forming a V-shaped trench, the ostial valves do not protrude deeply into the lumen of the heart. Each ostium is suspended in the myocard by two prominent bundles of fibers which run almost longitudinally. Anteriorly, an anterior aorta emanates from the heart and is separated from the heart by a vertically orientated aortic valve with paired lateral lips. Posteriorly, the heart ends blindly. Five pairs of cardiac arteries emanate ventrolaterally from the heart and are separated from the heart by a horizontally orientated valve with paired lips. The first pair of cardiac arteries emanates just posterior of the ostia, the other four pairs emanate in close vicinity to each other in the posterior portion of the heart.

#### “Crustacea”: Malacostraca: Peracarida: Isopoda: Asellota: *Asellus aquaticus*

The heart is tube-shaped and lies in the dorsal median line. It extends from the border between the fifth and sixth thoracomeres almost to the posterior end of the trunk. The myocard consists of a single layer of cardiomyocytes which are arranged helically (with a helical shift of around 16 to 20 cardiomyocytes per 360 °). The cardiomyocytes are shaped like semicircular arches with their junctions in the dorsal and ventral median line. There are two pairs of ostia which are situated laterally in the seventh and eighth thoracomeres. Each ostium is flat and has paired muscular valves. The two ostia of each pair are not situated directly opposite each other—the left lies more anteriorly than the right in each case. Anteriorly, an anterior aorta emanates from the heart and is separated from the heart by an aortic valve which is vertically orientated and has paired lips. Five pairs of cardiac arteries emanate ventrolaterally from the heart. Each cardiac artery is separated from the heart by an arterial valve which is horizontally orientated and has paired lips. The first pair of cardiac arteries (anterior lateral arteries) emanates from the heart at the anterior apex, close to the origin of the anterior aorta. The second to fourth pairs emanate in the posterior part of thoracic segments six to eight, respectively. The fifth pair of cardiac arteries emanates in the anterior pleon, close to the posterior apex of the heart. Posteriorly, the heart ends blindly.

#### “Crustacea”: Cephalocarida: *Hutchinsoniella macracantha*

We only had the opportunity to study few specimens left over from previous projects (see [56, 57]). However, phalloidin staining never works in every single specimen, especially not in specimens stored in PBS for some years. The only two successful stainings were performed in late larval stages. Heart morphology in Cephalocarids has only ever been described by Hessler & Elofsson [45]; with a focus on ultrastructure.

The heart lies in the dorsal median line (Fig 3A). It is tube-shaped and extends through the first six thoracic segments. The myocard consists of a single layer of cardiomyocytes which are shaped like semicircular arches. The contractile fibers within the cells are not arranged in any recognisable order (Fig 3B) but connect in a dorsal and a ventral longitudinal crest. There are three pairs of laterally situated ostia in the anterior half of the heart, all consisting of paired muscular valves which form a V-shaped trench. Anteriorly, the myocard forms a narrowed tip which could be interpreted as an excurrent valve to a putative anterior aorta (aortic valve; see discussion in [45]).

#### “Crustacea”: Remipedia: *Xibalbanus tulumensis*

**Fig 5:**
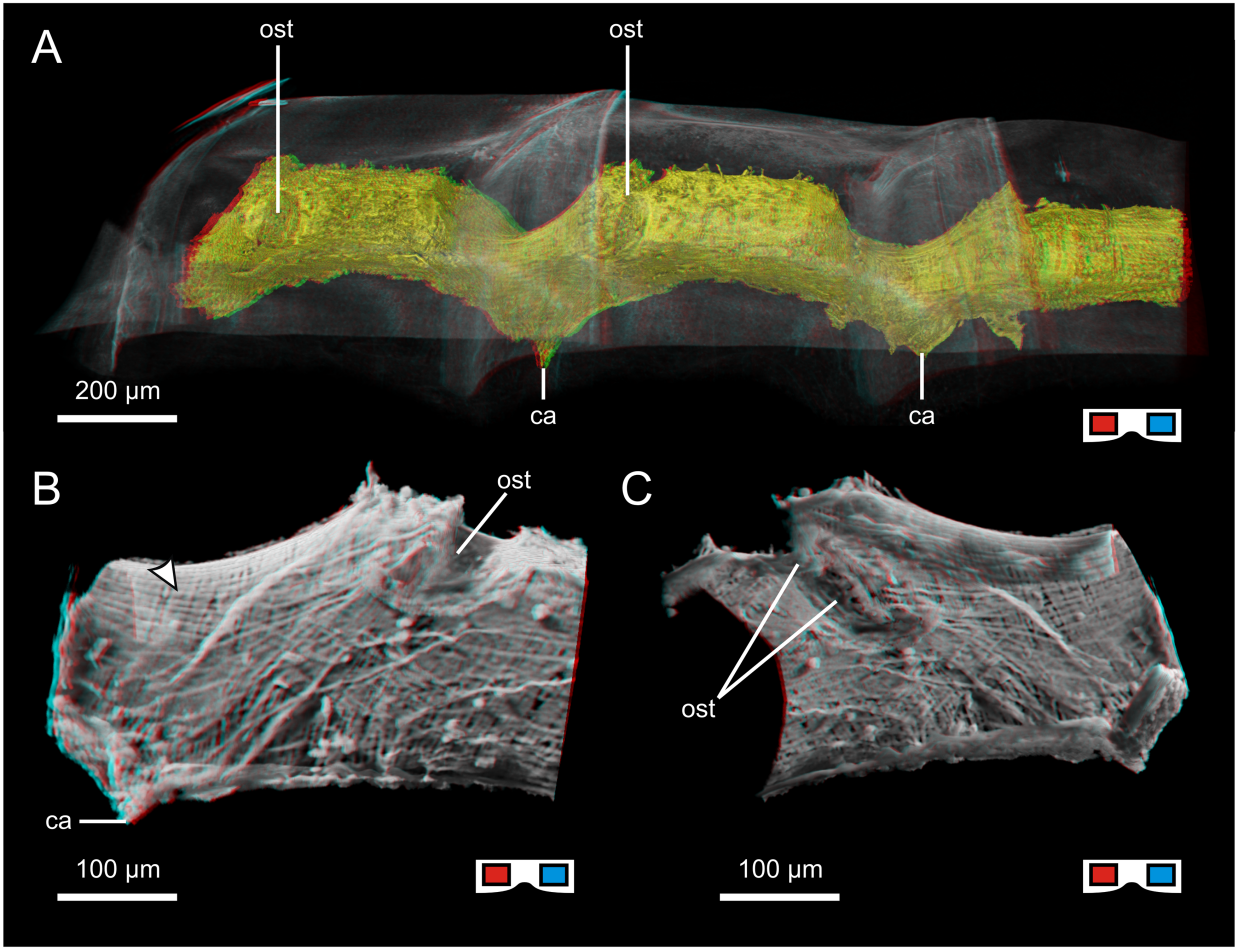
The heart of *Xibalbanus tulumensis* („Crustacea“: Remipedia;). All tiles are red cyan anaglyphs, use goggles to see images correctly. A: Volume rendering of the heart in the mid-posterior trunk segments (dorsolateral view, μCT); thinner regions at the border between two consecutive segments are visible beneath the overlap of the two respective tergites. B, C: Shadow projection of the outer (B) and inner (C) view of the heart in the anterior portion of the segment (lateral/medial view, μCT), arrowhead indicates longitudinal fibers of the epicard overlapping the semicircular cardiomyocytes. ca: cardiac artery; ost: ostium.

The heart lies in the dorsal median line and extends from the posterior margin of the head to the posterior end of the trunk. The heart is tube-shaped and the myocard consists of a single layer of cardiomyocytes which are arranged in parallel. The cardiomyocytes are shaped like semicircular arches with their junctions in the dorsal and ventral median line. The heart becomes thinner where it curves underneath the borders between adjacent tergites (Fig 5A). There are segmental ostia which are situated dorsolaterally and consist of paired muscular valves which form a V-shaped trench (Fig 5B and 5C). The ostial opening points in a posterior direction on both sides. Anteriorly, an anterior aorta emanates from the heart and is separated from the heart by a vertically orientated aortic valve with paired lateral lips. One pair of cardiac arteries emanates ventrolaterally in each segment.

#### Hexapoda: Machilidae: *Petrobius brevistylis*

**Fig 6:**
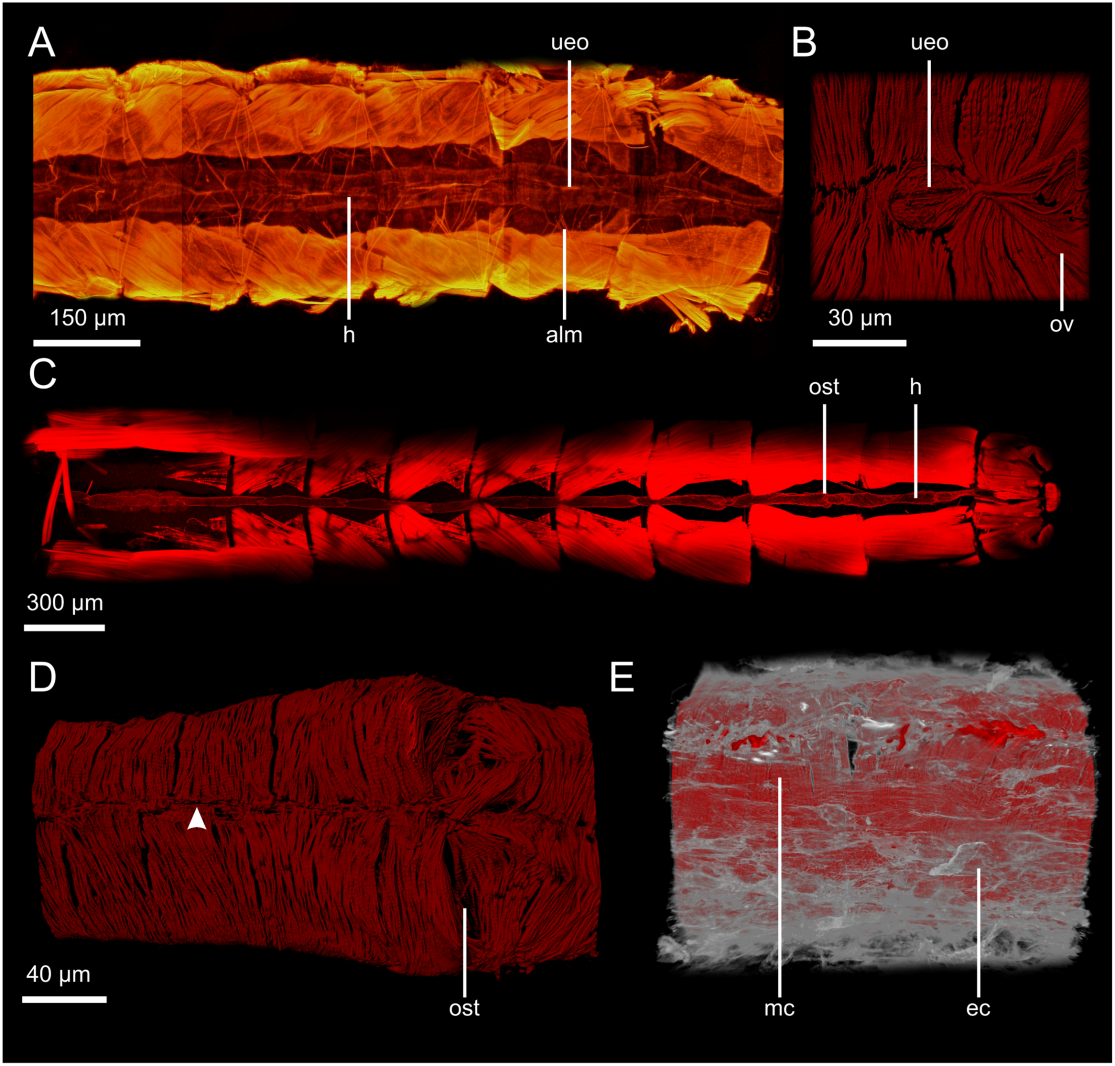
Hearts of insects. A: Heart of *Petrobius brevistylis* (ventral view, fluo), location of the heart between the longitudinal musculature. B: Unpaired excurrent opening in the ventral median line of the heart of *P*. *brevistylis* (dorsal view of the interior, ventral portion of the myocard, cLSM). C: Heart of *Procloeon bifidum*, location of the heart between the longitudinal musculature (ventral view, cLSM). D: Ostium of *P*. *brevistylis* (dorsal view, cLSM), arrowhead indicates the dorsal median line where cardiomyocytes are attached to each other. E: Epicard (grey) and myocard (red) of the heart of *Blaptica dubia* (dorsolateral view, cLSM). alm: alary muscle; ec: epicard; h: heart; mc: myocard; ost: ostium; ov: ostial valve; ueo: unpaired excurrent opening.

The heart lies in the dorsal median line and extends from the thorax to the posterior part of the ninth abdominal segment. The heart is tube-shaped (Fig 6A) and the myocard consists of a single layer of cardiomyocytes which are arranged in parallel. The cardiomyocytes are shaped like semicircular arches with their junctions in the dorsal and ventral median line (Fig 6D). There are segmental ostia in the abdomen which are situated dorsolaterally and consist of paired valves which protrude deep into the lumen. The ostial opening, of which only a slit is visible, points in a posterior direction on both sides (Fig 6D). On the luminal side, longitudinal myofibers run along the dorsal median line between the contralateral ostia of one pair of ostia and form a knot to which the ostia are attached. Anterior to each pair of ostia, an unpaired excurrent opening equipped with a paired valve is situated in the ventral median line, its opening orientated longitudinally (Fig 6B).

#### Hexapoda: Ephemeroptera: *Procloeon bifidum*

The heart lies in the dorsal median line and extends from the mid-posterior region of the thorax almost to the posterior end of the abdomen (Fig 6C). The heat is tube-shaped and the myocard consists of a single layer of cardiomyocytes. The myocard in the posterior part of the heart consists of a single layer of cardiomyocytes which are arranged helically with a helical shift of around 15 cardiomyocytes per 360 °. In the regions where ostia are situated, some additional cardiomyocytes are arranged differently. The segmental ostia (Fig 6C) are situated laterally and consist of paired valves which protrude deep into the lumen. The ostial opening, of which only a slit is visible, points in a posterior direction on both sides.

#### Hexapoda: Blattodea: *Blaptica dubia*

The heart lies in the dorsal median line and extends from the mesothorax to the posterior end of the abdomen. The heart is tube-shaped with a slightly greater diameter in the border regions between segments. This phenomenon is strong in the thorax but becomes weaker in a posterior direction. The heart follows the dorsal integument, displaying two z-folds in the thorax (under the wings) and slight bendings in the abdomen. The heart consists of an outer epicard and an inner myocard (Fig 6E). The epicard is made of mostly longitudinal fibers of connective tissue. The myocard consists of a single layer of broad, flat cardiomyocytes which are arranged in parallel and shaped like semicircular arches. The cell junctions are situated in the dorsal and ventral median line. Paired ostia are situated dorsolaterally in the posterior region of each segment. The ostia consist of paired muscular valves which protrude deep into the heart lumen. In the thorax, the ostia lie anterior to the z-folds. Seen from the outside, the ostia appear as mere slits and one prominent dorsal bundle of myofibers to which the ostial valves are attached runs longitudinally across the thickened area where the ostia are situated. The ostial opening points in a posterior direction on both sides. Anteriorly, an anterior aorta emanates from the heart. Paired cardiac arteries emanate from the heart, though we were unable to find them in each segment.

### Character conceptualization

In the following section, all our character conceptualizations are listed and explained. The characters in the conceptualizations are to be regarded as hypotheses on putative transformation series and their respective character states. Each conceptualization is formulated as a character statement, as proposed by Sereno [58], and takes the following form: *Locator(s)*, *variable*: *character state 0 (0)*, *…*, *character state n (n)*.

We understand characters as transformation series *sensu* Hennig [59, 60] and thus as natural kinds encompassing all the different homologous character states which evolved from the original character state in the common ancestor [24, 53, 59, 61]. Character conceptualizations are hypotheses on transformation series, and character states, “properly defined and delineated, are the real subject of adaptation” [62].

#### Overall shape and geometry

*1. Heart: absent (0); present (1)*.

This character refers to the presence or absence of the heart (OARCS_0000253) and is the prerequisite character for all further characters in this study. To our knowledge, the absence of a heart is equivalent to the absence of any kind of hemolymph vascular system. However, we chose this formulation in line with the focus on arthropod hearts in this study. If the heart in a species/taxon is *absent* (0), all further characters have to be coded as *inapplicable* (-).

*2. Heart, general shape: tube-like (0); fusiform (1); roughly spherical (2); dumbbell-shaped (3); trough-like anteriorly and tube-like posteriorly (4); trough-like (5)*.

This character refers to the overall shape of the heart. (0): Tube-like hearts are significantly longer than they are wide and exhibit the same diameter all the way along their length; e.g. Myriapoda. (1): Fusiform hearts are also significantly longer than they are wide but have an area of maximum diameter and then narrow towards both ends; Xiphosura. (2): Roughly spherical hearts can be ball- or sack-shaped. Length and width do not differ significantly; e.g. Decapoda. (3): Dumbbell-shaped hearts have a thickened anterior and posterior region and are narrow in the middle; e.g. Thermosbaenacea. (4): The anterior half of the heart forms a dorsally opened trough, only the posterior half of the heart is tube-like; e.g. Anostraca. (5): The heart is trough-like along its entire length. The lateral myocard is attached to the dorsal epidermis, which thus forms the dorsal closure of the heart; e.g. Pycnogonida.

*3. Heart, extension within trunk: approx. entire trunk (0); approx. half of the trunk, anteriorly (1); approx. half of the trunk, middle (2); approx. half of the trunk, posteriorly (3); only small portion, anteriorly (4); only small portion, middle (5)*.

This character refers to the extension of the heart within the trunk. The trunk has been chosen as a reference system due to the fact that statements on the homology of tagmata such as “thorax” or “abdomen” are problematic. Enumerating the individual segments is also problematic as the number of segments differs greatly within Arthropoda and again, homology relationships between segments are hard to unravel. Another problem with segments lies in the fact that in numerous arthropods, segments are no longer distinguishable. However, the extension of the heart in relation to the overall body is, in our view, a putative subject of specific selection pressures. (0): The heart extends more or less through the entire trunk; e.g. Myriapoda. (1): the heart extends through about half of the trunk and is situated in the anterior part of the trunk; e.g. Leptostraca. (2): the heart extends through about half of the trunk and is situated in the mid part of the trunk; e.g. Xiphosura. (3): the heart extends through about half of the trunk and is situated in the posterior part of the trunk; e.g. Araneae. (4): the heart extends only through a single segment or very few segments of the trunk and is situated in the anterior part in the trunk; e.g. Cladocera. (5): the heart extends only through a single segment or very few segments of the trunk and is situated in the mid part of the trunk; e.g. Decapoda. One might argue that putatively convergent reductions in heart length are found under this character state. However, this character refers to the significant developmental process leading to heart development in a restricted set of segments only.

#### Histology

*4. Heart, epicard: absent (0); present (1)*.

This character refers to the absence/presence of the epicard (OARCS_0000187), an outer layer of connective tissue which surrounds the myocard (see Fig 6E).

*5. Heart, myocard, cardiomyocytes, arrangement: meshwork around the lumen, most cells perpendicular to longitudinal axis (0); single layer of semicircular cells (1); meridional with dorsal and ventral knots (2); meshwork without dominant orientation and bundles running across the lumen (3); two distinct layers of fibers (4)*.

**Fig 7:**
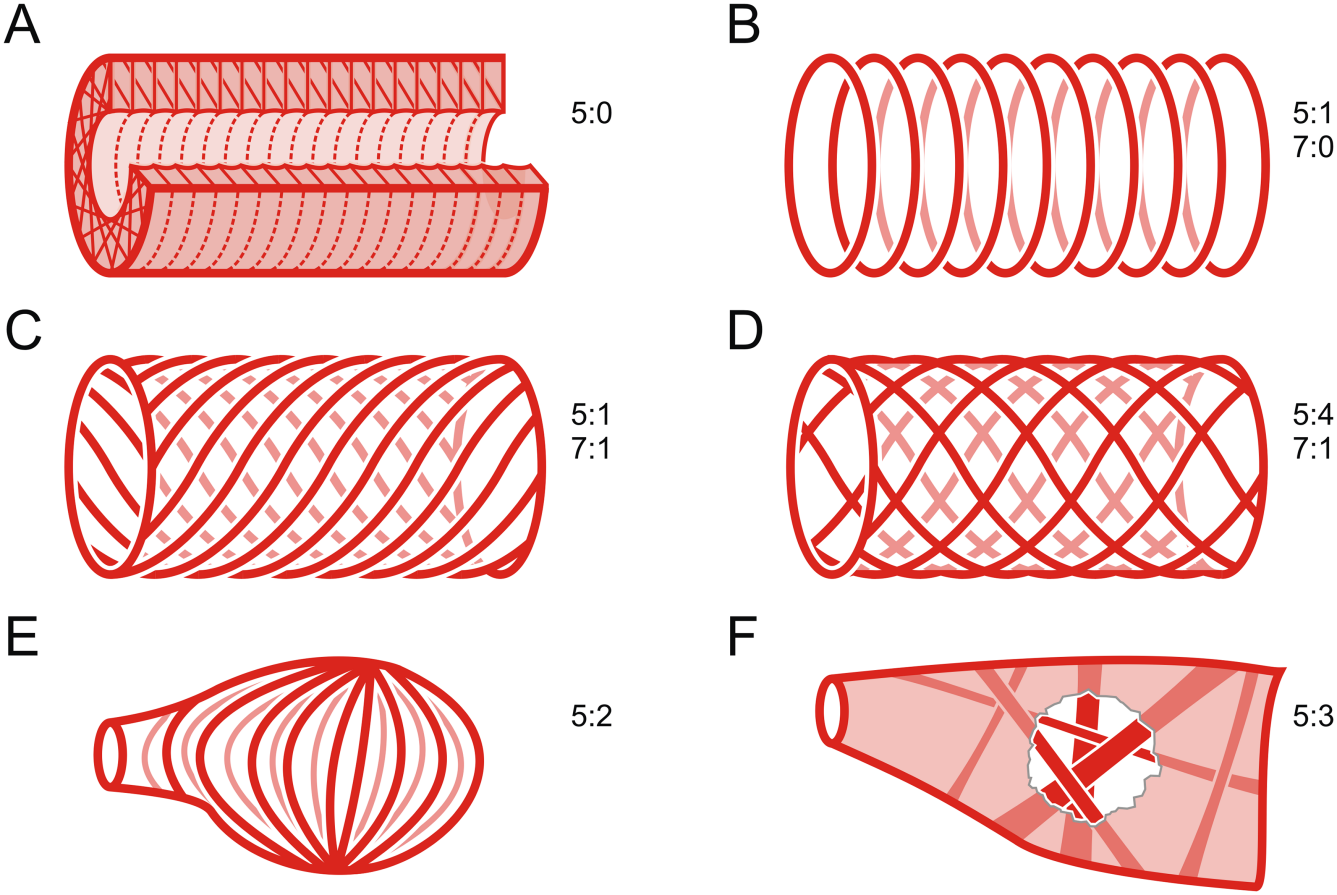
Schematic representations of the arrangement of cardiomyocytes in the myocard (character 5 and character 7). A: meshwork around the lumen, most cells perpendicular to longitudinal axis (5:0). B: single layer of semicircular cells without helical shift (5:1, 7:0). C: single layer of semicircular cells with helical shift (5:1, 7:1). D: two distinct layers of fibers with helical shift in opposite directions (5:4, 7:1). E: meridional with dorsal and ventral knots (5:2). F: meshwork without dominant orientation and bundles running across the lumen (5:3).

This character refers to the arrangement of the cardiomyocytes. Arrangement means the general pattern, although in specific regions (e.g. anterior/posterior apex or around the ostia) single cardiomyocytes can be arranged differently. (0): the cardiomyocytes branch and form a meshwork. However, most of the cardiomyocytes are perpendicular to the longitudinal axis; e.g. Xiphosura (Fig 7A). (1): the myocard consists of a single layer of semicircular cardiomyocytes. The cell junctions are found in the dorsal and ventral median line; e.g. Myriapoda (Fig 7B). (2): the cardiomyocytes are meridionally arranged and span from dorsal to ventral knots; e.g. Calanoida (Fig 7E). (3): the cardiomyocytes form a dense meshwork. There is no dominant orientation and single bundles of cardiomyocytes run across the lumen; e.g. Decapoda (Fig 7F). (4): two distinct layers of fibers can be recognized; e.g. Anaspidacea (Fig 7D).

*6. Heart, myocard, cardiomyocytes, distance between contractile parts of neighboring cardiomyocytes: directly adjoining (0); with space between (1)*.

This character refers to whether (the contractile parts of) neighboring (i.e. longitudinally consecutive) cardiomyocytes are (0): directly adjoining; e.g. Machilidae (Fig 6E and 6D) or (1) whether there is a gap between; e.g. Tanaidacea (Fig 4E).

*7. Heart, myocard, cardiomyocytes, semicircular cells, circumvolution: without helical shift (0); with helical shift (1)*.

This character refers exclusively to cardiomyocytes arranged semicircularly (character state 1 in character 5). (0): no helical shift, i.e. the cardiomyocytes that are connected via a junction in the dorsal median line are also connected in the ventral median line (sometimes these junctions are not 1-to-1 junctions, but there is still no constant shift pattern); e.g. Mysida (Figs 4F and 7B). (1): with helical shift, i.e. the cardiomyocytes that are connected via a junction in the dorsal median line are not connected in the ventral median line, thus leading to a spiral arrangement; e.g. Tanaidacea (Figs 4E and 7C).

*8. Heart, myocard, cardiomyocytes, helical shift, degree of shift: weak, i.e. 3 to 6 cardiomyocytes per 360 ° (0); strong, i.e. 14 to 20 cardiomyocytes per 360 ° (1)*.

This character refers to the helical shift of cardiomyocytes (character state 1 in character 7). Two distinct states can be distinguished. (0): a weak helical shift of three to six cardiomyocytes per 360 °; e.g. Euscorpiidae. (1): a strong helical shift of 14 to 20 cardiomyocytes per 360 °; e.g. Tanaidacea (Fig 4E).

#### Ostia

*9. Heart, ostia, shape: only a slit visible from the outside, muscular valves protrude deep into the lumen (0); deep V-shaped (horizontal section) trench; valves made up of several myofibers only protrude a little way into the lumen (1); shallow (sometimes almost flat) trench, most of the valve visible from outside (2); origin of ostial valves extruded; V-shaped trench of medium depth (3)*.

**Fig 8:**
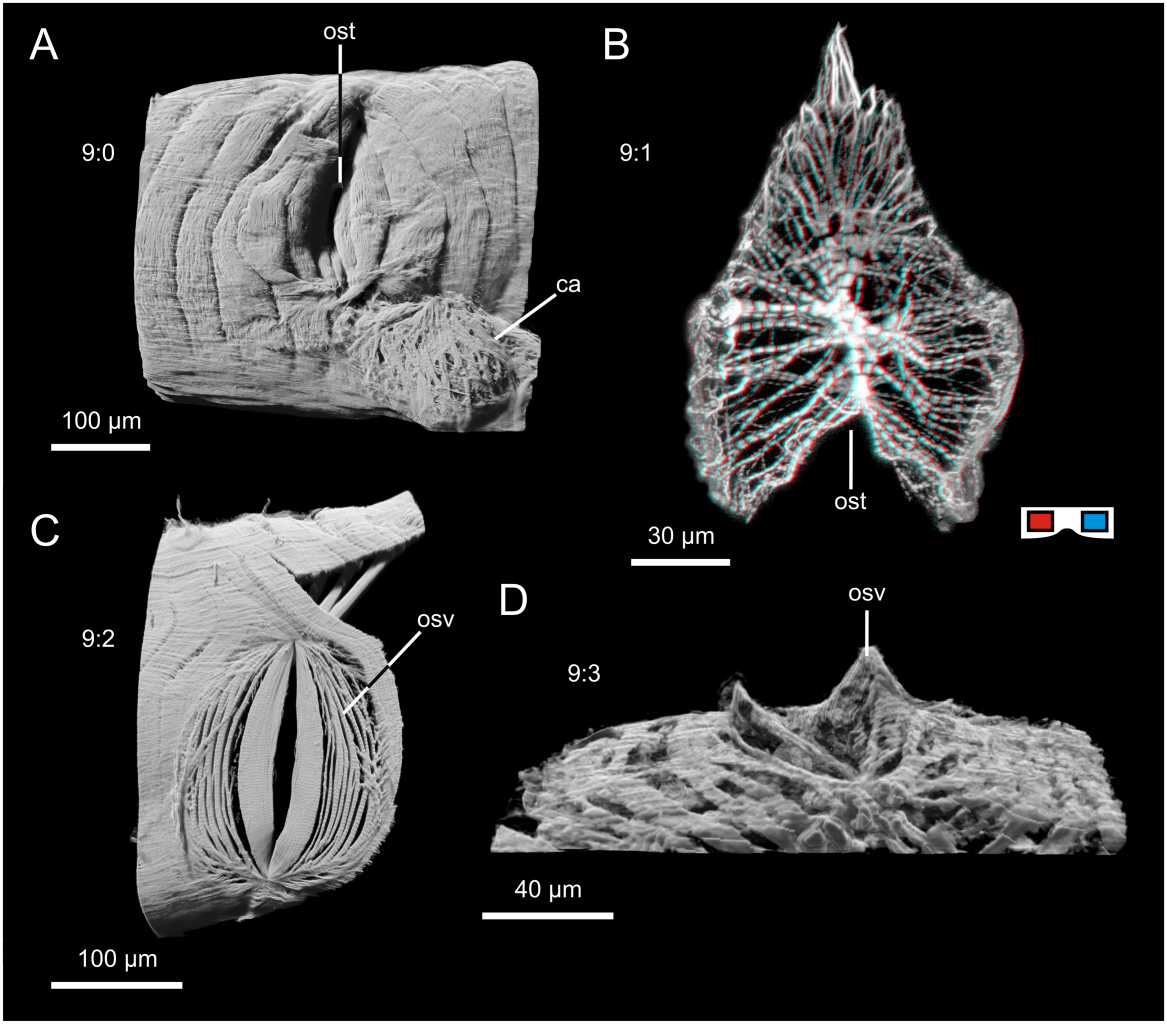
Exemplar for the character states of character 9. A: state 0, only a slit visible from the outside, muscular valves protrude deep into the lumen (example from *Euscorpius tergestrinus*, lateral view, cLSM). B: state 1: deep V-shaped (horizontal section) trench; valves which are made up of several myofibers only protrude a little way into the lumen (example from *Pseudodiaptomus pelagicus*, dorsal view, cLSM) red cyan anaglyph, use goggles; C: state 2: shallow (almost flat) trench, most of the valve visible from outside (example from *Nebalia herbstii*, lateral view, cLSM). D: state 3: origin of ostial valves extruded; V-shaped trench of medium depth (example from *Leucon nasica*, ventrolateral view, 3D visualized from histological sections).

This character refers to the overall shape of the ostia seen from outside the heart and in horizontal sections (Fig 8). (0): only a slit is visible from outside the heart (Fig 8A). The valves are regular cardiomyocytes that protrude deep into the lumen; e.g. Arachnida. (1): when seen from the outside or in horizontal sections, the valves of these ostia form a deep V-shaped trench (Fig 8B). The valves can thus be seen from the outside, more than a mere slit is visible. The valves are made up of several myofibers and protrude into the lumen, but not as far as in state 0; e.g. Calanoida, Remipedia. (2): The valves form a shallow trench or are almost flat (Fig 8C). Most of the valves, which are made up of several myofibers, can thus be seen from outside the heart; e.g. Leptostraca. (3): a V-shaped trench of medium depth (something between states 1 and 2) but with prominent extrusion of the origins of the valve (Fig 8D) which can be seen especially well in horizontal sections; e.g. Cumacea.

*10. Heart, ostia, arrangement, general pattern: evenly distributed, segmental (0); evenly distributed, non-segmental (1); unevenly distributed (2)*.

This character refers to the distribution of ostia in the heart in relation to the longitudinal axis. (0): A clear segmental pattern can be found. Ostia are evenly distributed along the full length of the heart, one pair of ostia is found in every segment; e.g. Myriapoda. (1): Ostia are evenly distributed along the full length of the heart, but no segmentality is observable; e.g. Araneomorphae. (2): Ostia are unevenly distributed; e.g. Decapoda. This character state is thought to be a manifestation of the evolutionary change in which the segmental development of ostia was overcome. Patterns of uneven distribution of ostia are further examined in character 11, but it can be said in general that this character state demonstrates how often and in which taxa the escape from segmentality in ostia distribution occurred. This character state also covers partially segmental/pseudosegmental arrangements, i.e. distributions which appear even (constant distances between the ostia) but which are restricted to a certain area of the heart rather than its full length.

*11. Heart, ostia, uneven distribution, pattern of arrangement: one single pair, halfway between the two ends (0); one single pair, anteriorly situated (1); one unpaired ostium at the posterior apex (2); 1st dorsolateral, 2nd lateral, 3rd ventrolateral (3); 1st antrodorsolateral, 2nd dorsal, 3rd dorsomedial, 4th ventrolateral, 5th posterolateral (4); segmental arrangement only in the anterior part (5); 1st to 3rd uneven, subsequent ostia segmental (6); 1st anterior, 2nd and 3rd halfway, one directly above the other (7); two pairs halfway, one directly above the other (8); one unpaired ostium anteriorly and one pair of ostia ~in the middle of the heart (9)*;

This character refers to the pattern of uneven distribution of ostia in the heart. While evenly distributed ostia (character 10, states 0 and 1) provide inflow of hemolymph into the heart all along its length, unevenly distributed ostia cause uneven inflow of hemolymph. If, for example, in a long tube-like heart a single pair of ostia is situated at the anterior apex of the heart, this represents to us a different character state than a single pair of ostia situated at half the length of the heart. The two different patterns provide different inflow of hemolymph and thus appear to be adaptations to different selective pressures. (0): The only pair of ostia in the heart is situated anteriorly; e.g. Cumacea. (1): The only pair of ostia in the heart is situated halfway between the two ends of the heart e.g. Anaspidacea. (2): An unpaired ostium only is situated medially at the posterior apex of the heart; e.g. Calanoida. (3): There are three pairs of ostia, the first is situated dorsolaterally, the second is situated laterally, and the third is situated ventrolaterally; e.g. Astacidea. (4): There are five pairs of ostia which are situated antrodorsolaterally, dorsally, dorsomedially, ventrolaterally, and posterolaterally; e.g. Pasiphaeidae. (5): There are segmental ostia, but only in the anterior half of the heart, posteriorly there are none; e.g. Leptostraca. (6): The first to third ostia are unevenly distributed but the following ostia are arranged segmentally; Stomatopoda. (7): There are three pairs of ostia, the first is situated anteriorly, the second and third are both situated halfway between the two ends of the heart, one directly above the other; e.g. Lophogastrida. (8): There are two pairs of ostia which are both situated halfway between the two ends of the heart, one directly above the other; Mysida. (9): There is an unpaired ostium situated rather anteriorly and a pair of ostia situated halfway between the two ends of the heart; e.g. Tanaidacea.

*12. Heart, ostia, position of paired ostia in relation to each other: opposite (0); diagonally shifted (1)*.

This character refers to paired ostia. The two ostia of a pair, i.e. the left and the right, can exhibit different relative positions to each other. (0): Left ostium and right ostium lie directly opposite each other; e.g. Myriapoda. (1): left ostium and right ostium are diagonally shifted with respect to each other; e.g. Buthidae.

*13. Heart, paired ostia, normal vector, angle in relation to longitudinal axis: orthogonal (0); pointing in a posterior direction on both sides (1); pointing in an anterior direction on one side and a posterior direction on the other side (2)*.

This character refers to the direction of the ostial opening indicated by its normal vector in relation to the longitudinal axis. Whether the ostial opening points dorsally, laterally or ventrally is not considered—the character refers only to whether the ostia point in an anterior direction, a posterior direction or neither. (0): The ostial opening points neither in an anterior nor in a posterior direction; e.g. Xiphosura. (1): The ostial opening points in a posterior direction on both sides; e.g. Machilidae. (2): The ostial opening points in an anterior direction on one side of the heart and in a posterior direction on the other side; e.g. Tanaidacea.

#### Arteries leaving the heart

*14*. *Anterior aorta: absent (0); present (1)*

This character refers to the presence or absence of an anterior aorta (OARCS_0000004). An anterior aorta is an unpaired longitudinal artery which leaves the heart anteriorly and can be considered the anterior part of what is often called the dorsal vessel.

*15. Anterior aorta, aortic valve, shape: single valve, dorsally attached (0); paired valves, laterally attached (1)*.

The aortic valve marks the transition from the heart to the anterior aorta. Two different kinds of aortic valve can be distinguished. (0): a single valve which is attached dorsally to the myocard. Valves like this hang scoop-like from the dorsal part of the myocard and bend anteriorly in their ventral portion to permit outflow of hemolymph from the heart into the aorta while preventing backflow; e.g. Xiphosura. (1): paired valves which are attached laterally to the myocard and thus form a vertical slit; e.g. Mysida.

*16. Posterior aorta: absent (0); present (1)*.

This character refers to whether a posterior aorta (OARCS_0000156) is present or the heart ends blindly. A posterior aorta is an unpaired longitudinal artery which leaves the heart posteriorly and can be considered the posterior part of what is often called the dorsal vessel.

*17. Cardiac arteries: absent (0); present (1)*.

This character refers to the presence or absence of cardiac arteries (OARCS_0000197) emanating from the heart.

*18. Cardiac arteries, arrangement, general pattern: evenly distributed, segmental (0); evenly distributed, non-segmental (1); unevenly distributed (2)*.

This character refers to the cardiac arteries leaving the heart. Cardiac arteries in arthropods emanate from the heart in different patterns. Though they can be paired or unpaired, that is not considered in this character as both conditions often occur in a single individual (e.g. in decapods). This character refers to the pattern of any cardiac arteries leaving the heart. (0): cardiac arteries emanate strictly segmentally from the heart along its full length; e.g. Diplopoda. (1): evenly distributed cardiac arteries emanate from the heart along its full length but do not seem to be associated with segments; e.g. Araneae. (2): unevenly distributed cardiac arteries emanate from the heart. This character state also makes reference to/covers partially segmental/pseudosegmental arrangements, i.e. distributions which appear even (constant distances between the cardiac arteries) but which are restricted to a certain area of the heart rather than its full length; e.g. Decapoda.

#### Additional remarks on problematic cells

In scorpions, the heart extends right the way through the mesosoma. We scored character 3 as state 0, i.e. extension of the heart through approx. the whole trunk. This is because, in our view, the metasoma of scorpions is derived to a more appendage-like state and we perceive the trunk as the main corpus of the body containing the main organ systems.

In our investigations of *Glomeris marginata*, only one pair of cardiac arteries per diplosegment was detectable (originating ventrally of the anterior pair of ostia). However, Leiber [34] described two pairs per diplosegment and we thus scored character 18 as state 0.

Although we did not detect an epicard in *Triops cancriformis*, we were not able to confirm its absence with confidence. As an epicard is present in *Lepidurus arcticus* [36], we scored character 4 as state 1 for Notostraca in the concatenated matrix.

Lowe [63] described three ostia in *Calanus finmarchicus*, whereas Park [64] only described a single unpaired ostium at the posterior apex of the heart for *Epilabidocera amphitrites*. Our investigations of three calanoid species revealed a single unpaired ostium at the posterior apex of the heart and we thus scored character 11 as state 2 for Calanoida in the concatenated matrix.

Huckstorf & Wirkner [27] described paired posterior arteries (which they termed “paired posterior aortae”) in krill. However, as a posterior aorta is explicitly unpaired (sensu OARCS_0000156; [13]), we interpret these paired arteries as cardiac arteries which run in a posterior direction (comparable to those found in Tanaidacea; see above and in [41]) and scored character 16 as state 0 for Euphausiacea.

## Discussion

### From morphemes to characters

**Fig 9:**
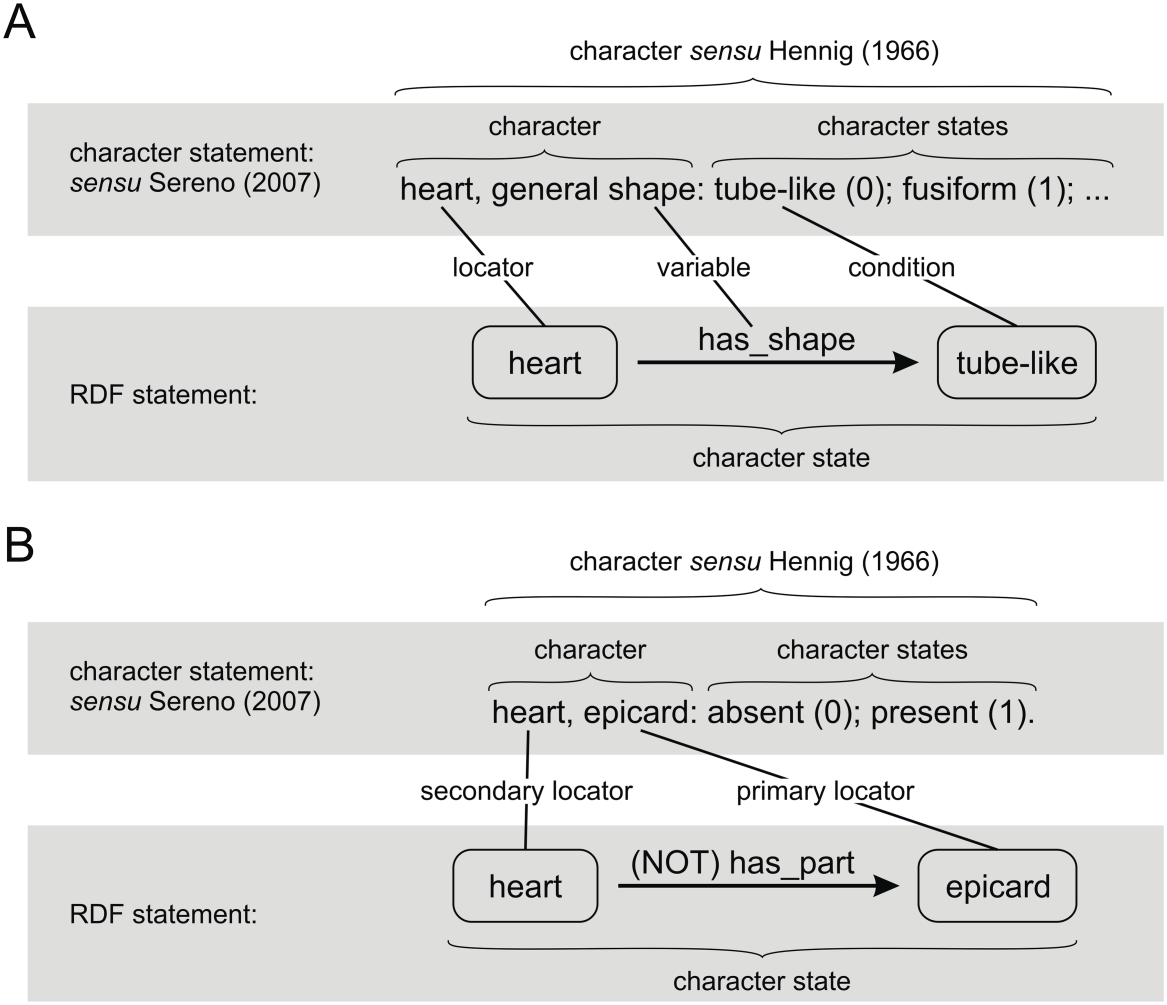
Relationships between components of character statements (*sensu* Sereno [58]) and RDF triplets describing character states. This scheme illustrates the differences between the definition of “character” *sensu* Sereno [58] and “character” *sensu* Hennig [59]. A: transformational characters (example: character 2). B: neomorphic characters (example: character 4).

In this study, the description of morphemes is followed by a conceptualization of characters and character states [5, 14]. All characters are formulated as character statements *sensu* Sereno [58]. By following this procedure, we have been able to formalize character concepts in a way which facilitates intersubjective understandability and enables the reader to immediately identify the constituents of the character statement. In a transformational character, the character statement consists of one or more locator(s), a variable and the possible conditions of the variable, i.e. character states (Fig 9). However, applying this to the character concept advocated by Hennig [59] throws up various ontological differences. According to Sereno [58], the character corresponds to the sum of the locator(s) and the variable (Fig 9). However, as Hennig’s ideographic character concept [60] includes all the respective character states, the character *sensu* Hennig [59] corresponds to the whole character statement (Fig 9) *sensu* Sereno [58]. As an example, let us look at our character no. 2: *Heart*, *general shape*: *tube-like (0); fusiform (1); roughly spherical (2); dumbbell-shaped (3); trough-like anteriorly and tube-like posteriorly (4); trough-like (5)*. In this character statement (Fig 9A), “heart” is the (primary) locator, “general shape” is the variable and “tube-like”, “fusiform”, “roughly spherical”, “dumbbell-shaped”, “trough-like anteriorly and tube-like posteriorly” and “trough-like” are the possible conditions of the variable (character states *sensu* Sereno [58]). We prefer to speak of “conditions of the variable” rather than character states, as the conditions themselves are not sufficient to constitute a character state, e.g. “fusiform” alone is not a character state (as no reference is made to the feature which takes this shape) but “heart, general shape: fusiform (1)” is a valid character state. This method of character formulation also visualizes the non-equivalence of morpheme and character/character state. It is obvious from this example that there is neither congruence between the heart as a morpheme (as a concrete *thing*; [24]) and the character as a whole (heart ≠ heart, general shape: tube-like (0), fusiform (1), …), nor between the heart as a morpheme and the individual character states (heart ≠ heart, general shape: fusiform (1)). Such congruence only exists between the morpheme and the primary locator (heart = heart). In transformational characters (Fig 9A), the variable represents the property of the primary locator (an attribute of form of the respective morpheme) which has evolved into different conditions. In neomorphic characters (i.e. absent/present characters; [58]; Fig 9B), however, there is no variable in the character statement, as the existence of the primary locator is the aspect which varies. As an example, let us take our character no. 4: *Heart*, *epicard*: *absent (0); present (1)*. The “epicard” is the primary locator (“heart” is the secondary locator) and there can only be congruence between the actual morpheme and the primary locator.

To achieve the desired semantic web compliance of an evolutionary morphology which uses ontologies [12, 13, 25, 65, 66], phenotypic statements can be formulated as RDF graphs [66, 67] which take the general form of triples [subject]-[property]-[object]. This applies both to descriptive statements for the description of morphemes [13] and to character states. In this approach, RDF graphs can be compared in order to identify putative character states. Identical RDF triples in the descriptions of the morphology of two species can indicate character state identity [61], while differences in RDF statements can point to different character states [24]. A comparison of RDF graphs represents the starting point for the transition from the descriptive to the comparative/phylogenetic level [24, 66]. An RDF triple statement for transformational characters could be [heart]-[has_shape]-[tube-like] (Fig 9A). In this case, the primary locator is the subject of the triple (and is thus an instance of the ontology concept with the label ‘heart’), the variable is a property (i.e. ontological relationship) and the condition of the variable is the object of the triple statement (in this case an instance of the ontology concept with the label ‘tube-like’). The triple as a whole [heart]-[has_shape]-[tube-like], then, represents the character state of the respective species. In neomorphic characters, the presence/absence of a feature can be expressed in RDF via (negated) has_part relationships [66], e.g. [heart]-[has_part]-[epicard] (Fig 9B). In RDF triples which use the has_part relationship to express neomorphic characters, the primary locator is the object of the statement rather than the subject as in transformational characters. In neomorphic characters a has_part relationship is established (or negated) between the secondary locator and the primary locator to form the character state, while in transformational characters, other relationships (depending on the variable) are established between the primary locator and the respective condition to form the character state.

### Reconstruction of ancestral states

Reconstruction of ancestral character states is a tool often used in evolutionary biology. Its aim is to optimize character transformations from the inferred character state in the stem species of the investigated taxa to the character states in the terminals [68]. Character state reconstruction constitutes an inferred explanatory hypothesis which can only make use of those character states which have been conceptualized a priori (although ancestral species might well have shown character states which cannot be found in recent taxa and thus cannot be reconstructed). The outcome, i.e. the hypothetical character states of the (also hypothetical) ancestral species, are not to be conflated with descriptive statements. Descriptive statements about hypothetical morphemes cannot reasonably be formulated on the basis of the inferred ancestral character states, which are themselves products of generalization and conceptualization [5, 22, 24].

We are aware of the objections to character mapping raised by Fitzhugh [69], among others. However, the phylogenetic hypotheses which provide the basis for our reconstruction also include hypotheses on population splitting events, i.e. hypotheses on the actual sequential events in arthropod evolution. To us, mapping our data onto these hypotheses constitutes a “given this scenario” approach which seeks to explore putative scenarios of character transformation against the background of a specific hypothesis on phylogenetic relationships as a reflection of past population splitting events.

In the following, hypotheses on the character states in the ground pattern of five major taxa are given in telegraphic style based on our reconstruction of ancestral states. Ambiguities are pointed out but no attempt is made here to resolve them. Evolutionary scenarios and arguments in favor of a certain character state in ambiguous nodes will be provided in the section “Evolutionary transformations of characters”.

Arthropoda: **1**. Heart: present; **2**. Heart, general shape: tube-like *OR* fusiform *OR* trough-like; **3**. Heart, extension: through approx. entire trunk; **4**. Epicard: present; **5**. Heart, myocard, cardiomyocytes, arrangement: single layer of semicircular cells; **6**. Heart, myocard, cardiomyocytes, distance between contractile parts of neighboring cardiomyocytes: directly adjoining; **7**. Heart, myocard, cardiomyocytes, semicircular cells, circumvolution: without helical shift; **9**. Heart, ostia, shape: only a slit visible from the outside, muscular valves protrude deep into the lumen *OR* deep V-shaped (horizontal section) trench; **10**. Heart, ostia, arrangement, general pattern: evenly distributed, segmental *OR* evenly distributed, non-segmental; **12**. Heart, ostia, position of paired ostia in relation to each other: opposite; **13**. Heart, paired ostia, normal vector, angle in relation to longitudinal axis: orthogonal; **14**. Anterior aorta: present; **15**. Anterior aorta, aortic valve, shape: single valve, dorsally attached *OR* paired valves, laterally attached; **16**. Posterior aorta: absent; **17**. Cardiac arteries: present; **18**. Cardiac arteries, arrangement, general pattern: evenly distributed, segmental.

Chelicerata: **1**. Heart: present; **2**. Heart, general shape: tube-like *OR* fusiform *OR* trough-like; **3**. Heart, extension: through approx. entire trunk; **4**. Epicard: present; **5**. Heart, myocard, cardiomyocytes, arrangement: single layer of semicircular cells; **6**. Heart, myocard, cardiomyocytes, distance between contractile parts of neighboring cardiomyocytes: directly adjoining; **7**. Heart, myocard, cardiomyocytes, semicircular cells, circumvolution: without helical shift; **9**. Heart, ostia, shape: only a slit visible from the outside, muscular valves protrude deep into the lumen *OR* deep V-shaped (horizontal section) trench; **10**. Heart, ostia, arrangement, general pattern: evenly distributed, segmental *OR* evenly distributed, non-segmental; **12**. Heart, ostia, position of paired ostia in relation to each other: opposite; **13**. Heart, paired ostia, normal vector, angle in relation to longitudinal axis: orthogonal; **14**. Anterior aorta: present; **15**. Anterior aorta, aortic valve, shape: single valve, dorsally attached *OR* paired valves, laterally attached; **16**. Posterior aorta: absent; **17**. Cardiac arteries: present; **18**. Cardiac arteries, arrangement, general pattern: evenly distributed, segmental.

Mandibulata: **1**. Heart: present; **2**. Heart, general shape: tube-like; **3**. Heart, extension: through approx. entire trunk; **4**. Epicard: present; **5**. Heart, myocard, cardiomyocytes, arrangement: single layer of semicircular cells; **6**. Heart, myocard, cardiomyocytes, distance between contractile parts of neighboring cardiomyocytes: directly adjoining; **7**. Heart, myocard, cardiomyocytes, semicircular cells, circumvolution: without helical shift; **9**. Heart, ostia, shape: only a slit visible from the outside, muscular valves protrude deep into the lumen *OR* deep V-shaped (horizontal section) trench; **10**. Heart, ostia, arrangement, general pattern: evenly distributed, segmental *OR* evenly distributed, non-segmental; **12**. Heart, ostia, position of paired ostia in relation to each other: opposite; **13**. Heart, paired ostia, normal vector, angle in relation to longitudinal axis: orthogonal; **14**. Anterior aorta: present; **15**. Anterior aorta, aortic valve, shape: paired valves, laterally attached; **16**. Posterior aorta: absent; **17**. Cardiac arteries: present; **18**. Cardiac arteries, arrangement, general pattern: evenly distributed, segmental.

Myriapoda: **1**. Heart: present; **2**. Heart, general shape: tube-like; **3**. Heart, extension: through approx. entire trunk; **4**. Epicard: present; **5**. Heart, myocard, cardiomyocytes, arrangement: single layer of semicircular cells; **6**. Heart, myocard, cardiomyocytes, distance between contractile parts of neighboring cardiomyocytes: directly adjoining; **7**. Heart, myocard, cardiomyocytes, semicircular cells, circumvolution: without helical shift; **9**. Heart, ostia, shape: only a slit visible from the outside, muscular valves protrude deep into the lumen; **10**. Heart, ostia, arrangement, general pattern: evenly distributed, segmental; **12**. Heart, ostia, position of paired ostia in relation to each other: opposite; **13**. Heart, paired ostia, normal vector, angle in relation to longitudinal axis: orthogonal; **14**. Anterior aorta: present; **15**. Anterior aorta, aortic valve, shape: paired valves, laterally attached; **16**. Posterior aorta: absent; **17**. Cardiac arteries: present; **18**. Cardiac arteries, arrangement, general pattern: evenly distributed, segmental.

Tetraconata: **1**. Heart: present; **2**. Heart, general shape: tube-like; **3**. Heart, extension: through approx. entire trunk; **4**. Epicard: present; **5**. Heart, myocard, cardiomyocytes, arrangement: single layer of semicircular cells; **6**. Heart, myocard, cardiomyocytes, distance between contractile parts of neighboring cardiomyocytes: directly adjoining *OR* with space between; **7**. Heart, myocard, cardiomyocytes, semicircular cells, circumvolution: without helical shift; **9**. Heart, ostia, shape: deep V-shaped (horizontal section) trench; **10**. Heart, ostia, arrangement, general pattern: evenly distributed, segmental *OR* evenly distributed, non-segmental *OR* unevenly distributed; **12**. Heart, ostia, position of paired ostia to each other: opposite; **13**. Heart, paired ostia, normal vector, angle in relation to longitudinal axis: orthogonal; **14**. Anterior aorta: present; **15**. Anterior aorta, aortic valve, shape: paired valves, laterally attached; **16**. Posterior aorta: absent; **17**. Cardiac arteries: present; **18**. Cardiac arteries, arrangement, general pattern: evenly distributed, segmental.

The ancestral morphology of the circulatory system has hardly been discussed for Arthropoda and accounts found in textbooks such as Gruner [70] or Clarke [71] barely touch on heart structure. Rather, these works focus on the mere presence of hearts and vessels and their relationship to the metameric organization of what is hypothesized to be the stem species of arthropods. To date, ancestral conditions in the arthropod circulatory system (including heart structure and the presence of certain arteries) have been deduced intuitively from single exemplar species and the commonly accepted hypothesis of a strictly segmented arthropod ancestor (e.g. [72, 73]), or even treated as textbook knowledge (e.g. [74]). However, a replicable parsimony-based reconstruction of ancestral character states taking into account underlying phylogenetic hypotheses has never been carried out. In previous accounts, the then-prevailing Articulata hypothesis turned discussion of the arthropod circulatory system into a comparison with that of annelids, with circulatory systems in both groups (often) comprising longitudinal dorsal and ventral vessels as the main channels. This led to the conclusion that the open vascular system found in arthropods and onychophorans [75] represents a kind of reduction of the closed vascular system of annelids [70, 71]. The Ecdysozoa hypothesis, however, has new implications for the evolution of circulatory systems in (Pan-)Arthropoda, as all members of Cycloneuralia are devoid of dedicated circulatory organs [4]. The metameric circulatory system in onychophorans and arthropods thus likely evolved convergently with that of annelids. The circulatory system of Onychophora consists of a tube-like heart with segmental slit-like ostia and no cardiac arteries leaving the heart [75]. This indicates that the ancestral condition in a monophylum of Onychophora and Arthropoda comprises a dorsal heart which extends the entire way through the trunk and is equipped with segmental ostia. However, whether cardiac arteries were present in the common ancestor of onychophorans and arthropods and have been reduced in onychophorans or whether cardiac arteries represent an autapomorphy of Arthropoda cannot be said with certainty. In onychophorans, the heart opens into an anterior aorta which then gives rise to antennal vessels [76]. It thus appears plausible that the common ancestor of Onychophora and Arthropoda possessed a tube-like heart extending right the way through the trunk and anteriorly giving rise to the anterior aorta. Cardiac arteries might have evolved in the ancestral lineage of Arthropoda as a feature which greatly improved the efficiency of the circulatory system, directing hemolymph flow within the body and distributing freshly oxygenated hemolymph evenly to the organs in need. For the stem species of Arthropoda then, we reconstruct a heart which extended right the way through the trunk, giving rise to an anterior aorta and segmental cardiac arteries. We avoid the term dorsal vessel in this context, as according to this study, a posterior aorta is not part of the arthropod ground pattern (see below) and the heart ended blindly. The morphology of the arthropod myocard, however, is yet to be discussed in an evolutionary context.

### Evolutionary transformation of characters

In this section, we discuss putative scenarios for evolutionary character transformations based on the parsimony reconstruction of ancestral states. We do this in detail for two exemplary characters (characters 5 and 9) but also provide some additional remarks on the other characters. Some of the reconstructed ambiguities are discussed in this section too, and arguments in favor of preferred reconstructions are explored.

Character ***5***. *Heart*, *myocard*, *cardiomyocytes*, *arrangement*: *meshwork around the lumen*, *most cells perpendicular to longitudinal axis (0); single layer of semicircular cells (1); meridional with dorsal and ventral knots (2); meshwork without dominant orientation and bundles running across the lumen (3); two distinct layers of fibers (4)*.

**Fig 10:**
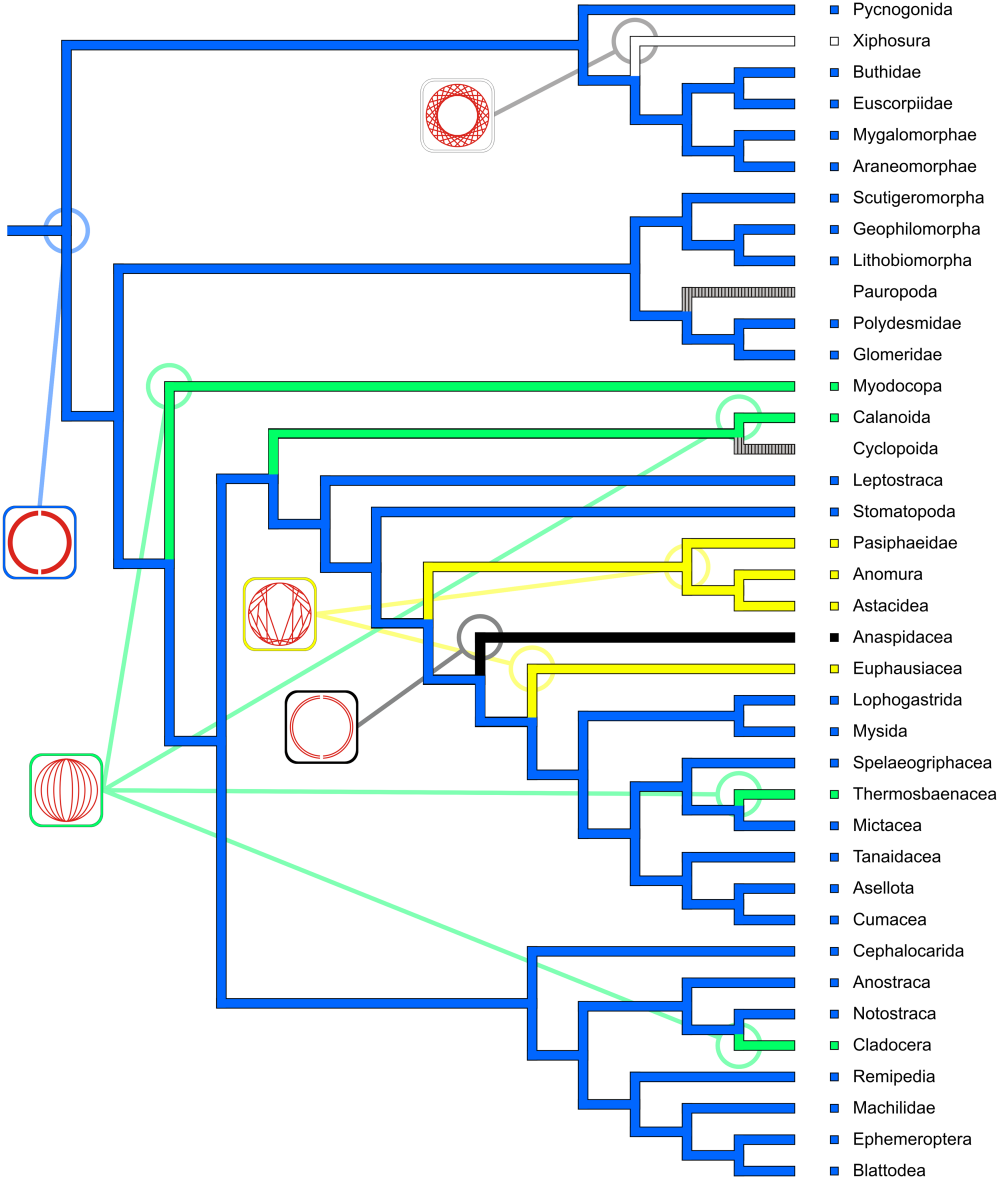
Parsimony-based reconstruction of the evolutionary transformation of character 5. 5: Heart, myocard, cardiomyocytes, arrangement: white: meshwork around the lumen, most cells perpendicular to longitudinal axis (0); blue: single layer of semicircular cells (1); green: meridional with dorsal and ventral knots (2); yellow: meshwork without dominant orientation and bundles running across the lumen (3); black: two distinct layers of fibers (4). The pictograms show schematic cross sections of the myocard to illustrate the respective character states. This is one of the most parsimonious reconstructions, for ambiguities see S1 File.

Based on the reconstruction (Fig 10), we assume that in the ground pattern of Arthropoda the myocard was made up of a single layer of cardiomyocytes shaped like semicircular arches. This is the state found not only in the majority of taxa, it is also the only condition found at all in Arachnida, Myriapoda and Hexapoda. This character seems to be fairly conservative, though distinct transformations of the original character state appear in Xiphosura, Cladocera, Calanoida and some malacostracan taxa. In Cladocera, Calanoida and Thermosbaenacea, a myocard made up of cardiomyocytes arranged meridionally evolved convergently. Individuals in these taxa are minute, and hearts are short and sack-like, suggesting that miniaturization could be behind the transformation from the original character state to the meridional arrangement of cardiomyocytes (see also [4]). In other minute arthropods, a heart is completely lacking, e.g. Pauropoda, Cyclopoida. In these taxa, hemolymph flow is driven by locomotory musculature only. It is imaginable that body motion-mediated hemolymph flow might be sufficient below a critical body size (see also [4]). The conditions found in Myodocopa or Calanoida, then, represent an intermediate character state in the course of the miniaturization coherent with heart reduction and might have been present the stem species of e.g. Copepoda. Xiphosura display an autapomorphic character state in having a myocard made up of a dense, sponge-like meshwork of cardiomyocytes which run perpendicular to the longitudinal axis only. The thick myocard in horseshoe crabs appears to be very powerful (see below) and might be the key factor in enabling the heart to efficiently supply the highly complex arterial system in these large arthropods [30]. The phylogenetic relationships within Malacostraca are far from being resolved (e.g. [5, 77, 78]). One main issue is the phylogenetic position of Euphausiacea, which the two main hypotheses frame either as the sister group to Decapoda [79] or the sister group to Peracarida [77]. With respect to character 5, these alternative hypotheses yield different interpretations of the evolutionary transformation of this character. Decapods and krill possess condensed hearts with a thick myocard (character 5 state 3). They exhibit no distinct layers but are composed of a strong meshwork of cardiomyocytes. The cardiomyocytes do not run in one particular direction, meaning that numerous vectors of contraction can act together. Furthermore, bundles of cardiomyocytes run through the heart lumen in various directions, which might additionally increase the efficiency of the contraction of the heart muscle. The so-called “globular heart” (OARCS_0000007) is still discussed as a putative synapomorphy of Decapoda and Euphausiacea (e.g. [27]). The high efficiency of the globular heart is necessary in these taxa as they possess complex arterial systems [27, 28], and decapods can reach large body sizes. In our reconstruction, based on the hypothesis of a sister group relationship between Euphausiacea and Peracarida, the decision on whether the globular heart in decapods and krill evolved only once or twice convergently cannot be made (for an illustration of the ambiguity of this character see S1 File). The autapomorphic character state found in Anaspidacea, a long tube-like heart with a double-layered myocard (character 5 state 4), does not help in solving this problem. However, the anaspidacean myocard can easily be imagined as having evolved from the single-layer myocard by duplication. This scenario, which involves convergence of the globular hearts in decapods and krill, appears more plausible than the evolution of the double-layer anaspidacean myocard from the globular heart and a reversal to a single-layer myocard in the evolution of Peracarida (see Fig 10 and character 9 in S1 File). A more detailed comparison not only of the morphology, but also of the development of the globular heart in decapods and krill, then, is required to establish putative convergence. If this peculiar condition happened to evolve twice with a high degree of similarity, the same genetic changes would be assumed to have occurred in decapods and euphausiaceans.

Character ***9***. *Heart*, *ostia*, *shape*: *only a slit visible from the outside*, *muscular valves protrude deep into the lumen (0); deep V-shaped (horizontal section) trench; valves made up of several myofibers only protrude a little way into the lumen (1); shallow (sometimes almost flat) trench*, *most of the valve visible from outside (2); origin of ostial valves extruded; V-shaped trench of medium depth (3)*.

**Fig 11:**
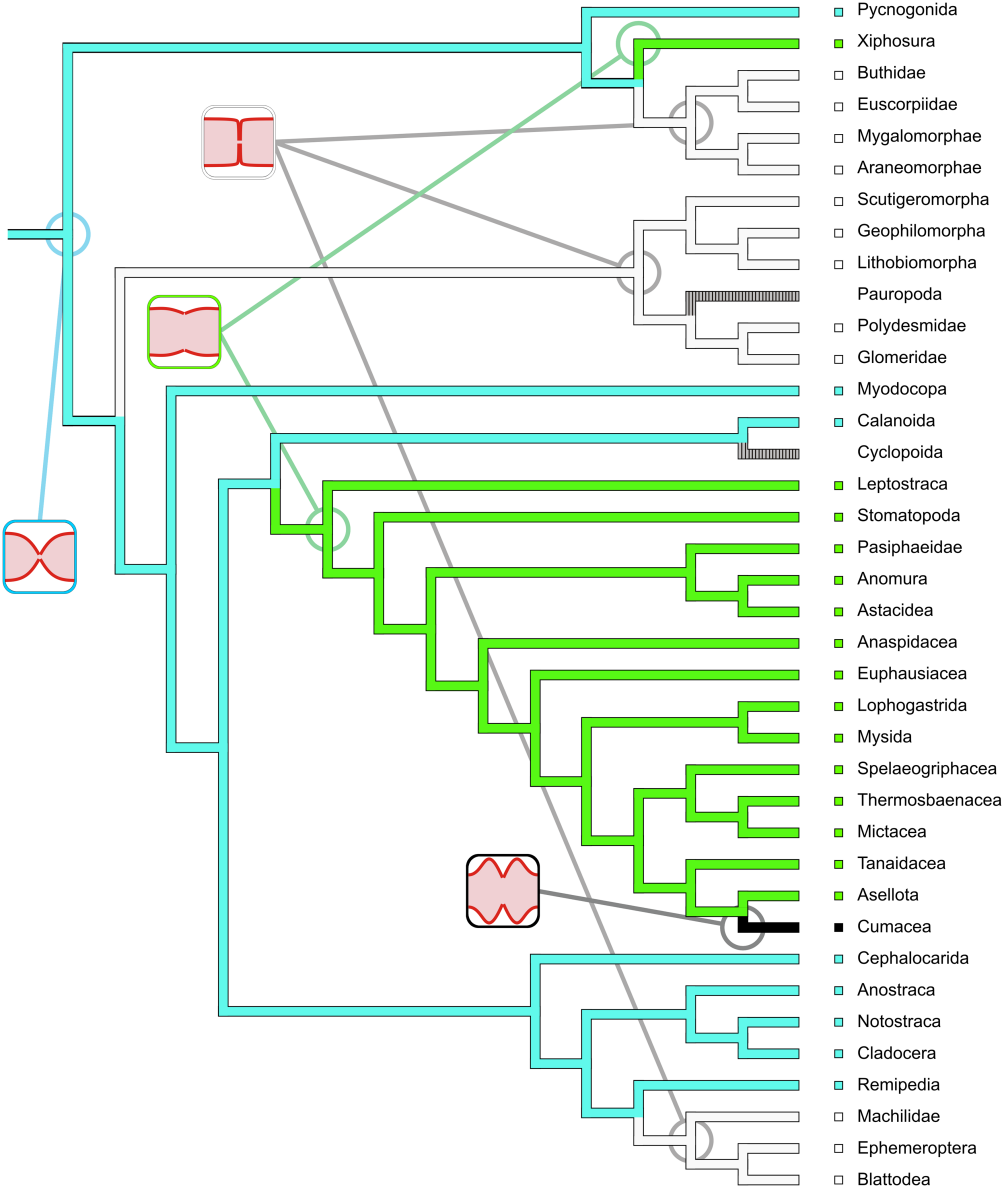
Parsimony-based reconstruction of the evolutionary transformation of character 9. 9: Heart, ostia, shape: white: only a slit visible from the outside, muscular valves protrude deep into the lumen (0); light blue: deep V-shaped (horizontal section) trench; valves made up of several myofibers only protrude a little way into the lumen (1); green: shallow (sometimes almost flat) trench, most of the valve visible from outside (2); black: origin of ostial valves extruded; V-shaped trench of medium depth (3). The pictograms show schematic horizontal sections of the ostia to illustrate the respective character states. This is one of the most parsimonious reconstructions, for ambiguities see S1 File.

Ostia in arthropods have generally been conceived of as mere slit-like openings. Though this holds true for insects, myriapods and arachnids, groups of aquatic origin have a different type of ostium (Fig 11): deep V-shaped trenches (state 1; found in Pycnogonida and the non-malacostracan Crustacea) or shallow ostia (state 2). Our reconstruction resulted in two possibilities for the arthropod ground pattern: the stem-species of Arthropoda either had a slit-like ostium or a deep, trenched ostium (state 0 or 1; see S1 File, character 9). The question cannot be resolved by comparison to velvet worms as detailed morphological investigations of ostia are lacking for Onychophora. However, from the data it is obvious that the shape of ostia strongly correlates with habitat (aquatic or terrestrial). From what is known about early arthropods from the fossil record, arthropods most likely evolved in marine habitats [74]. The correlation between ostia shape and habitat leads to the hypothesis that in the arthropod stem species, the ostial valves formed a deep V-shaped trench (state 1). From this plesiomorphic character state, then, the shallow ostium found in horseshoe crabs and malacostracans evolved convergently via the further divergence of the origins of the ostial valves. In the course of terrestrialization in Arachnida, Myriapoda and Hexapoda, the slit-like ostium evolved via the convergence of the origins of the ostial valves. Although current knowledge does not permit us to suggest a causal factor for the occurrence of this character state, a connection between circulatory system morphology and terrestrialization has been shown in various different regards [4]. All malacostracan species studied (except for Cumacea) exhibit the same character state: shallow, sometimes flat ostia, i.e. the ostial valves do not protrude into the heart lumen but are flush with the outer surface of the heart. In Cumacea, however, a unique apomorphic character state has evolved. The ostial valves of cumaceans have remarkably extruded origins (Fig 8D) and form a clinched V-shaped trench of medium depth. The tips of the ostial valves do not protrude significantly into the heart lumen. Although the trench is reminiscent of the character state found in non-malacostracan crustaceans, derivation from the malacostracan condition is easily imaginable (and obviously more parsimonious). A possible scenario for this transformation is that the origins of the ostial valves became more and more extruded while the tips of the ostial valves remained in the same position relative to the outer surface of the heart, thus causing a trench to develop secondarily.

The reconstructions of all 18 characters can be found as a single pdf file in the supplementary data (S1 File). Certain aspects of the characters not mentioned in detail above will be discussed in the following.

In most arthropods, the heart is tube-shaped or fusiform and extends through a large part of the trunk. However, the shape and extension of the heart have undergone evolutionary transformations in numerous arthropod taxa. Shortened hearts, for example, evolved at least five times independently (six times if the dumbbell-shaped hearts of Thermosbaenacea are considered shortened as well). Interestingly, the evolutionary transformation towards a shortened heart appears to have happened for different functional reasons. In calanoid copepods, for example, organisms are minute, rendering a complex vascular system superfluous (cyclopoids and harpacticoids do not have hearts or vessels at all). The small, delicate heart in Calanoida seems merely to support the locomotory structures in hemolymph circulation. In decapods, on the other hand, a shortened heart is anything but the product of simplification. The bundles of cardiomyocytes running across the lumen permit contraction along numerous vectors. The condensation undergone by the decapod globular heart, then, served to make it more efficient.

A helical arrangement of semicircular cardiomyocytes evolved at least three times independently (Scorpiones, Eumalacostraca, Ephemeroptera). It is possible this arrangement not only constricts the heart but also permits a slight contraction along its length, presumably allowing it to pump more efficiently.

While ostia and cardiac arteries are segmentally arranged in the arthropod ground pattern, an evolutionary tendency to overcome segmental patterns is detectable in Tetraconata, where we find uneven distributions of ostia and cardiac arteries and even the reduction of cardiac arteries in some crustaceans and the majority of hexapods [26].

Our reconstruction of ancestral states indicates that the historically established term “dorsal vessel” (OARCS_0000002) [4, 13], which refers to the sum of the heart and the anterior and posterior aortae, needs to be regarded as more conceptual than evolutional. A posterior aorta (character 16) is not present in the arthropod ground pattern nor in the ground patterns of Chelicerata, Mandibulata or Tetraconata (according to our reconstruction). A posterior aorta in the form of an unpaired longitudinal vessel evolved at least three times within Arthropoda (in Arachnida, Malacostraca, Hexapoda).

Recent phylogenomic studies reinforce the sister group relationship of Remipedia and Hexapoda [49]. Our reconstruction shows, that ostia pointing in a posterior direction (character 13 state 1) is a putative morphological synapomorphy supporting this sister group relationship.

### Shapes of hearts and their functional impact

As seen above, hearts in arthropods have undergone a huge number of evolutionary transformations. In other words, the form and function of hearts have been altered by evolution (as the form-function complex is what is acted upon by natural selection [80]). Physiological studies on heart muscles have been carried out for several exemplar arthropod species (e.g. [81–84]), but functional analyses of hemolymph flow and comparisons of pumping efficiency in different types of hearts are difficult to perform, partly because of differences in body size. Particle flow analyses might represent a way of obtaining these comparisons, and thanks to new technical methods they are now being carried out in arthropods [85]. Nevertheless, as function arises directly from form [80], morphology suffices to allow us to draw functional conclusions. In this section we therefore seek to explore putative causes for functional changes during the evolution of arthropod hearts.

Besides the mere presence or absence of a heart, several aspects of heart morphology are to be considered crucial for functional reasons. The relative length of a heart is obviously directly correlated with the relative amount of hemolymph to be expelled during systole. The diameter of a heart and the thickness of the myocard are key variables in volumetric change via heart contraction (as will be shown below). Since it is the cardiomyocytes which contract, and their contraction takes place along the vector of their length, the arrangement of cardiomyocytes, which has been shown to be highly disparate in Arthropoda (see above, e.g. Fig 7), has various functional implications. The number and arrangement of ostia can vary from a high number of segmental ostia (e.g. in Diplopoda) to just a few (e.g. Tanaidacea) or even only one (e.g. Calanoida). The ostia are responsible for the re-entry of hemolymph into the heart during diastole. Therefore, suction through the ostia plays an important role in the direction of overall hemolymph flow within the hemocoel. The presence of arteries leaving the heart is just as important with regard to this aspect of function. While nearly all arthropods possess an anterior aorta (except for Branchipoda and Cephalocarida), the heart often lacks lateral cardiac arteries and a posterior aorta. It is fairly clear that hemolymph flow in species lacking cardiac arteries (e.g. Ephemeroptera) will differ to that in species with segmental cardiac arteries (e.g. Diplopoda). We will now try to analyze the functional impacts of evolutionary changes in heart morphology in relation to all these aspects (heart loss, number and arrangement of ostia and cardiac arteries, spiral arrangement of cardiomyocytes, thickness of the myocard, the globular heart of decapods and krill).

#### Loss of the heart

Although hearts have been present since the rise of arthropods [4], secondary loss of vascular structures has occurred in several lineages (e.g. Pauropoda, Cyclopoida; see above). Hemolymph circulation is vital for the transport of oxygen/carbon dioxide and nutrients, but as the locomotor movement of muscles and appendages, intestinal peristalsis [70] and the flexion of the body as a whole can suffice to circulate hemolymph in minute organisms (see also [4]), specialized circulatory organs became superfluous and were reduced. In the case of free-living copepods, cyclopoids and harpacticoids lack any vascular structure whatsoever, while calanoids exhibit a delicate sack-like heart with a short anterior aorta and a single unpaired ostium at its posterior apex. Functionally, it is possible that the calanoid heart only suffices to determine the direction of hemolymph flow to be anteriad in the dorsal part of the body and posteriad in the ventral part. Although calanoids tend to be slightly larger than cyclopoids and harpacticoids, the question of why they need a heart remains unanswered.

#### Number and arrangement of ostia and cardiac arteries

In the context of hemolymph circulation in an arthropod’s body, the number and arrangement of cardiac inlets and outlets will evidently have an impact on flow pattern. Contrasting conditions help us to understand the role of ostia and cardiac arteries. Ephemeroptera (and other insect taxa [26]), Geophilomorpha (see above; [32]) and Craterostigmomorpha [32] possess segmental ostia. However, cardiac arteries are lacking in all three taxa and the only outlet is the anterior aorta. Hemolymph flow is driven by the large number of ostia which suction hemolymph from all segments of the abdomen/trunk into the heart lumen. The role of the outlet, i.e. the anterior aorta, is primarily to supply the head region (especially the compound eyes and antennae) with hemolymph. Arthropods with extensive cardiac artery systems (e.g. Xiphosura [30], Araneae [31], Decapoda [28]) generally possess more powerful hearts (see below), indicating that the overall circulation of hemolymph is driven more by the discharge of hemolymph through the outlets. The presence and direction of the cardiac arteries also underline the need in these taxa for body regions other than the head to be supplied explicitly with freshly enriched hemolymph (rather than hemolymph which has already provided some of its nutrients and oxygen to the head region, as is the case in taxa without cardiac arteries).

#### Spirally arranged cardiomyocytes

The central functional principle when it comes to hearts is contraction. The different arrangements of cardiomyocytes found in arthropod myocards (Fig 7) are the cause of differences in the function of these hearts. The plesiomorphic (and widespread) arrangement of cardiomyocytes as parallel semicircular arches (Fig 7B; character states 5:1, 7:0) allows contraction to reduce heart volume only by means of constriction. Spirally arranged cardiomyocytes (Fig 7C and 7D; character states 5:1, 7:1 and 5:4, 7:1) have evolved in several arthropod lineages, i.e. scorpions, stomatopods, anaspidaceans, several peracarids and insects. The spiral arrangement of cardiomyocytes adds another dimension to the contractile function of the heart, augmenting constriction with contraction along the longitudinal axis, possibly in order to further increase volume reduction. However, contraction along the longitudinal axis is limited by the elasticity of the heart and the spatial constraints constituted by arteries leaving the heart.

#### Thickness of the myocard

**Fig 12:**
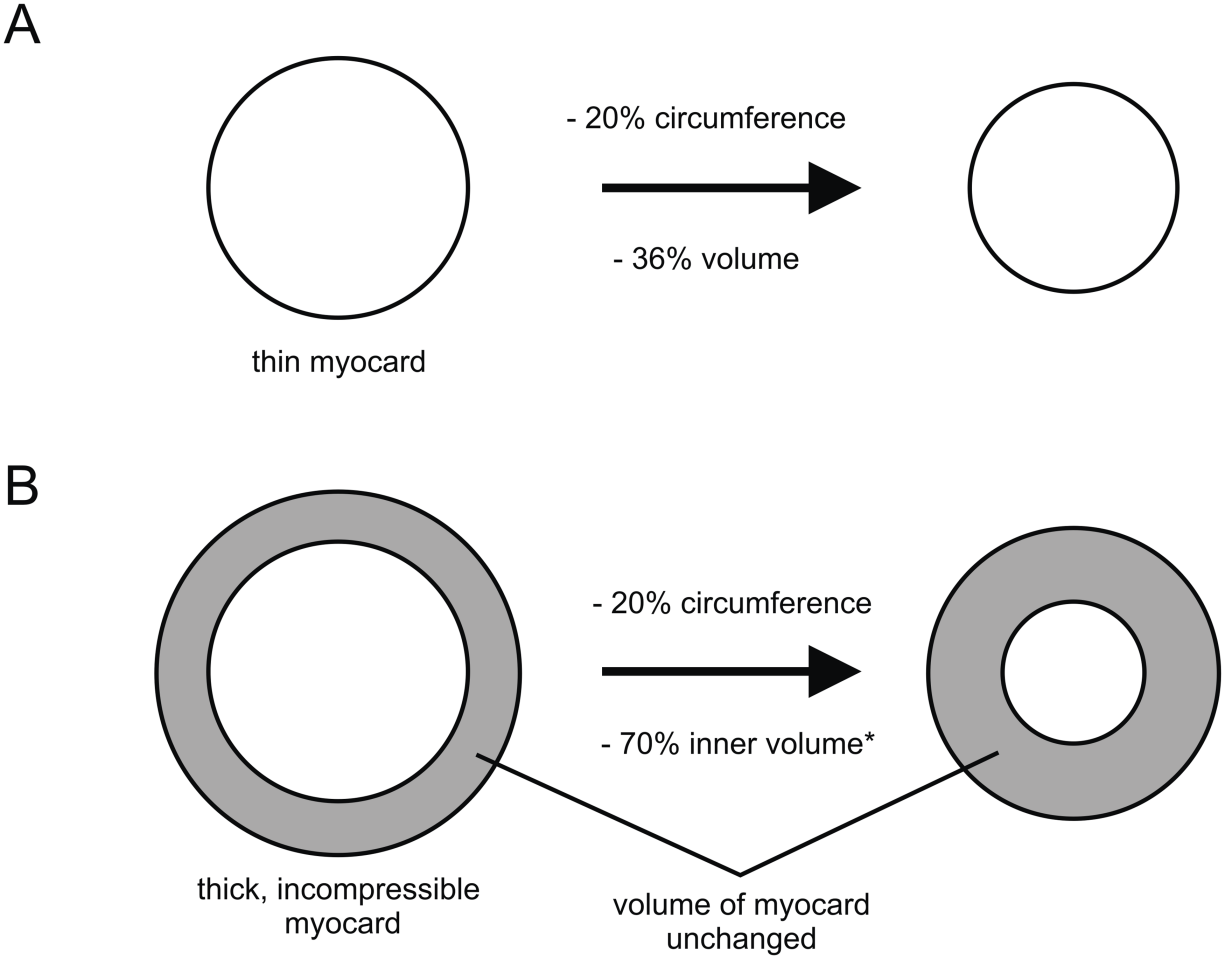
Schematic representation of the impact of a thick, incompressible myocard on relative volume reduction via constriction during systole in tubular hearts (adapted from Vogel [86] for spherical hearts). (*calculation based on the original scale of the drawing with a luminal diameter of 15 mm and myocard thickness of 6 mm during diastole).

The thickness of the myocard varies remarkably in arthropods. Single-layer myocards made up of semicircular cardiomyocytes (character state 5:1) often tend to be delicate, as in Remipedia and Diplopoda, for example. Hearts in Xiphosura are “tubular hearts” (OARCS_0000008), as in most other arthropods, but their thick myocard with its dense meshwork of cardiomyocytes (Fig 7A) indicate a significantly higher pumping efficiency [30]. Vogel [86] illustrated the geometric relationship between hearts with thin walls and hearts with thick, incompressible walls (Fig 12). A thicker heart wall means that with the same percentage of reduction in external circumference a higher reduction in the heart lumen is effected (Fig 12B) [86]. Though this is a strictly geometrical phenomenon, it hints towards a possible evolutionary explanation for why horseshoe crab hearts are relatively large in diameter compared to overall body size [30]. To efficiently pump hemolymph through a highly complex vascular system [30] in the large horseshoe crab body, the myocard evolved to be thicker while the heart lumen remained the same size. This optimized the geometrical relationship, enabling a higher volume of hemolymph to be expelled with every heartbeat. This principle also applies in the hearts of Scutigeromorpha, where the individual cardiomyocytes are significantly thicker than in other chilopods [32], providing more pumping efficiency in these active predators.

#### Globular hearts

The globular hearts in decapods and krill exhibit an arrangement of cardiomyocytes which differs significantly from that found in other arthropods (Fig 7F). The evolutionary transformations and hypothetical intermediate conditions undergone in these groups have been discussed by Wilkens [87] (see also [88]). However, Wilkens only considered the lobster/crayfish heart, which has three pairs of ostia, while caridid shrimps such as *Pasiphaea* have five pairs (see above). Nevertheless, Wilkins’ scenario of S-shaped bending and condensation remains the only explanatory scenario for globular heart evolution. The functional implications of the morphology of globular hearts have rarely been discussed. The globular heart is considered to be highly efficient with respect to its relatively small size [4, 88, 89], and as in Xiphosurans, the complex vascular systems in decapods and krill [27, 28] can be assumed to require a high level of efficiency in generating hemolymph pressure. Why the heart in these groups is restricted to the posterior cephalothorax, however, remains a crucial question. The restriction may have evolved because a substantial part of the cephalothorax is occupied by the stomach and the hepatopancreas and the pleon basically consists of pleonal muscles only [37], leaving the posterior cephalothorax the only space within the body where the heart could possibly be situated. To guarantee sufficient pumping efficiency under these circumstances, the myocard evolved to its recent condition. The globular heart found in decapods and euphausiaceans exhibits a thick outer myocard which offers the same advantages as the thick myocard in horseshoe crabs (see above, Fig 12; [86]). Additionally, thick bundles of myofibers run through the heart lumen (Figs 4G and 7F), adding new vectors of contraction in various directions. Further, the inner bundles of cardiomyocytes, which are incompressible, thicken to retain their absolute volume within the heart lumen during systole (the volume decrease of around 0.002% in contracting muscles is negligible, [90]). This increases the relative reduction in volume and thus also the relative amount of hemolymph which is expelled. Our reconstruction of the evolution of heart morphology implies a putative convergence of the globular hearts of decapods and krill (see Discussion above). Although convergence can be considered debatable due to the high number of congruities, the morphological constraints constituted by the large amount of space occupied by the digestive system and the pleonal musculature were certainly present in both taxa and may indeed have driven the convergent evolution of globular hearts in the two groups. As matters stand, the origin of the globular heart will remain an important object of evolutionary morphological investigation.

## Supporting information

S1 Table Scored matrix with all 18 characters for 45 species (Table 1). (PDF)

S2 Table Concatenated matrix with all 18 characters for 38 supraspecific taxa. (PDF)

S1 File Reconstruction of ancestral states illustrated with composite cladogram (based on Regier et al. [48], Wirkner & Richter [5] and Schwentner et al. [49]). (PDF)
