# Supplementary material for: Morphological description, character conceptualization and the reconstruction of ancestral states exemplified by the evolution of arthropod hearts
Source: PLoS One. 2018 Sep 20;13(9):e0201702. doi: 10.1371/journal.pone.0201702 (PMC6147405; doi:10.1371/journal.pone.0201702)
Supplement: S1 Table — (PDF) [file pone.0201702.s001.pdf]

Taxon / Character	1	2	3	4	5	6	7	8	9	10	11	12	13	14	15	16	17	18
Endeis	1	5	0	1	1	0	0	-	1	1	-	0	0	1	?	0	0	-
Limulus polyphemus	1	1	2	1	0	0	-	-	2	0	-	0	0	1	0	0	1	2
Euscorpius tergestrinus	1	1	0	1	1	0	1	0	0	0	-	0	0	1	0	1	1	0
Hottentotta hottentotta	1	1	0	1	1	0	1	1	0	0	-	1	0	1	0	1	1	0
Lasiodora parahybana	1	1	3	1	1	0	0	-	0	1	-	0	0	1	0	1	1	1
Araneus diadematus	1	1	3	1	1	0	0	-	0	1	-	0	0	1	0	1	1	1
Scutigera coleoptrata	1	0	0	0	1	0	0	-	0	0	-	0	0	1	1	0	1	0
Geophilus flavus	1	0	0	?	1	0	0	-	0	0	-	0	1	1	?	0	0	-
Lithobius forficatus	1	0	0	1	1	0	0	-	0	0	-	0	1	1	1	0	1	2
Pauropus silvaticus	0	-	-	-	-	-	-	-	-	-	-	-	-	-	-	-	-	-
Glomeris marginata	1	0	0	1	1	0	0	-	0	0	-	0	0	1	?	0	1	0
Polydesmus complanatus	1	0	0	1	1	0	0	-	0	0	-	0	0	1	?	0	1	0
Doloria levis	1	2	4	1	2	1	-	-	1	1	-	0	0	1	1	0	1	2
Artemia	1	4	0	0	1	0	0	-	1	0	-	0	0	0	-	0	0	-
Triops cancriformis	1	1	1	?	1	0	0	-	1	0	-	0	0	?	?	0	0	-
Lepidurus arcticus	1	?	?	1	1	0	0	-	?	?	?	?	?	?	?	?	?	?
Daphnia magna	1	2	4	0	2	1	-	-	1	1	-	0	0	0	-	0	0	-
Centropages typicus	1	2	5	?	2	1	-	-	1	2	2	-	-	1	1	0	0	-
Eurytemora affinis	1	2	5	?	2	1	-	-	1	2	2	-	-	1	1	0	0	-
Pseudodiaptomus pelagicus	1	2	5	?	2	1	-	-	1	2	2	-	-	1	1	0	0	-
Cyclops	0	-	-	-	-	-	-	-	-	-	-	-	-	-	-	-	-	-
Munida sarsi	1	2	5	1	3	0	-	-	2	2	3	0	0	1	1	1	1	2
Procambarus fallax f. virginalis	1	2	5	1	3	0	-	-	2	2	3	0	0	1	1	1	1	2
Pasiphaea multidentata	1	2	5	1	3	0	-	-	2	2	4	0	0	1	1	1	1	2
Nebalia	1	0	1	1	1	0	0	-	2	2	5	0	0	1	1	1	1	0
Paranebalia longipes	1	0	1	1	1	0	0	-	2	2	5	0	0	1	1	1	1	0
Gonodactylaceus falcatus	1	0	0	1	1	0	1	1	2	2	6	1	0	1	1	1	1	0
Anaspides tasmaniae	1	0	0	1	4	0	1	?	2	2	1	0	0	1	1	1	1	2
Meganyctiphanes norvegica	1	2	5	1	3	0	-	-	2	2	8	0	0	1	1	0	1	2
Asellus aquaticus	1	0	3	1	1	1	1	1	2	1	-	1	0	1	1	0	1	2
Leucon nasica	1	1	1	1	1	1	0	-	3	2	0	0	0	1	1	0	1	2
Diastyloides biplicatus	1	1	1	?	1	1	0	-	3	2	0	0	0	1	1	0	1	2
Diastylis tumida	1	1	1	?	1	1	0	-	3	2	0	0	0	1	1	0	1	2
Hemilamprops uniplicatus	1	1	1	1	1	1	0	-	3	2	0	0	0	1	1	0	1	2
Lophogaster typicus	1	0	1	1	1	0	1	?	2	2	7	0	0	1	1	1	1	0
Mictocaris halope	1	0	1	1	1	1	1	?	2	2	1	0	0	1	1	0	0	-
Neomysis integer	1	1	1	1	1	0	0	-	2	2	8	0	0	1	1	1	1	2
Spelaeogriphus lepidops	1	0	1	1	1	1	1	?	2	1	-	0	0	1	1	0	0	-
Apseudes spinosus	1	0	2	?	1	1	1	1	2	2	9	0	2	1	1	0	1	2
Tethysbaena argentarii	1	3	4	1	2	1	-	-	2	2	0	0	1	1	1	0	0	-
Hutchinsoniella macrantha	1	0	1	1	1	1	0	-	1	2	5	0	0	0	-	0	0	-
Xibalbanus tulumensis	1	0	0	1	1	0	0	-	1	0	-	0	1	1	1	?	1	0
Petrobius brevistylis	1	0	0	1	1	0	0	-	0	0	-	0	1	1	1	1	1	0
Procloeon bifidum	1	0	3	?	1	0	1	1	0	0	-	0	1	1	?	1	0	-
Blaptica dubia	1	0	0	1	1	0	0	-	0	0	-	0	1	1	?	0	1	2
