# Supplementary material for: Morphological description, character conceptualization and the reconstruction of ancestral states exemplified by the evolution of arthropod hearts
Source: PLoS One. 2018 Sep 20;13(9):e0201702. doi: 10.1371/journal.pone.0201702 (PMC6147405; doi:10.1371/journal.pone.0201702)
Supplement: S2 Table — (PDF) [file pone.0201702.s002.pdf]

Taxon / Character	1	2	3	4	5	6	7	8	9	10	11	12	13	14	15	16	17	18
Pycnogonida	1	5	0	1	1	0	0	-	1	1	-	0	0	1	?	0	0	-
Xiphosura	1	1	2	1	0	0	-	-	2	0	-	0	0	1	0	0	1	2
Euscorpiidae	1	1	0	1	1	0	1	0	0	0	-	0	0	1	0	1	1	0
Buthidae	1	1	0	1	1	0	1	1	0	0	-	1	0	1	0	1	1	0
Mygalomorphae	1	1	3	1	1	0	0	-	0	1	-	0	0	1	0	1	1	1
Araneomorphae	1	1	3	1	1	0	0	-	0	1	-	0	0	1	0	1	1	1
Scutigeromorpha	1	0	0	0	1	0	0	-	0	0	-	0	0	1	1	0	1	0
Geophilomorpha	1	0	0	?	1	0	0	-	0	0	-	0	1	1	?	0	0	-
Lithobiomorpha	1	0	0	1	1	0	0	-	0	0	-	0	1	1	1	0	1	2
Pauropoda	0	-	-	-	-	-	-	-	-	-	-	-	-	-	-	-	-	-
Glomeridae	1	0	0	1	1	0	0	-	0	0	-	0	0	1	?	0	1	0
Polydesmidae	1	0	0	1	1	0	0	-	0	0	-	0	0	1	?	0	1	0
Myodocopa	1	2	4	1	2	1	-	-	1	1	-	0	0	1	1	0	1	2
Anostraca	1	4	0	0	1	0	0	-	1	0	-	0	0	0	-	0	0	-
Notostraca	1	1	1	1	1	0	0	-	1	0	-	0	0	?	?	0	0	-
Cladocera	1	2	4	0	2	1	-	-	1	1	-	0	0	0	-	0	0	-
Calanoida	1	2	5	?	2	1	-	-	1	2	2	-	-	1	1	0	0	-
Cyclopoida	0	-	-	-	-	-	-	-	-	-	-	-	-	-	-	-	-	-
Anomura	1	2	5	1	3	0	-	-	2	2	3	0	0	1	1	1	1	2
Astacidea	1	2	5	1	3	0	-	-	2	2	3	0	0	1	1	1	1	2
Pasiphaeidae	1	2	5	1	3	0	-	-	2	2	4	0	0	1	1	1	1	2
Leptostraca	1	0	1	1	1	0	0	-	2	2	5	0	0	1	1	1	1	0
Stomatopoda	1	0	0	1	1	0	1	1	2	2	6	1	0	1	1	1	1	0
Anaspidacea	1	0	0	1	4	0	1	?	2	2	1	0	0	1	1	1	1	2
Euphausiacea	1	2	5	1	3	0	-	-	2	2	8	0	0	1	1	0	1	2
Asellota	1	0	3	1	1	1	1	1	2	1	-	1	0	1	1	0	1	2
Cumacea	1	1	1	1	1	1	0	-	3	2	0	0	0	1	1	0	1	2
Lophogastrida	1	0	1	1	1	0	1	?	2	2	7	0	0	1	1	1	1	0
Mictacea	1	0	1	1	1	1	1	?	2	2	1	0	0	1	1	0	0	-
Mysida	1	1	1	1	1	0	0	-	2	2	8	0	0	1	1	1	1	2
Spelaeogriphacea	1	0	1	1	1	1	1	?	2	1	-	0	0	1	1	0	0	-
Tanaidacea	1	0	2	?	1	1	1	1	2	2	9	0	2	1	1	0	1	2
Thermosbaenacea	1	3	4	1	2	1	-	-	2	2	0	0	1	1	1	0	0	-
Cephalocarida	1	0	1	1	1	1	0	-	1	2	5	0	0	0	-	0	0	-
Remipedia	1	0	0	1	1	0	0	-	1	0	-	0	1	1	1	?	1	0
Machilidae	1	0	0	1	1	0	0	-	0	0	-	0	1	1	1	1	1	0
Ephemeroptera	1	0	3	?	1	0	1	1	0	0	-	0	1	1	?	1	0	-
Blattodea	1	0	0	1	1	0	0	-	0	0	-	0	1	1	?	0	1	2
