# Supplementary figures and images for: Morphological description, character conceptualization and the reconstruction of ancestral states exemplified by the evolution of arthropod hearts
Source: PLoS One. 2018 Sep 20;13(9):e0201702. doi: 10.1371/journal.pone.0201702 (PMC6147405; doi:10.1371/journal.pone.0201702)

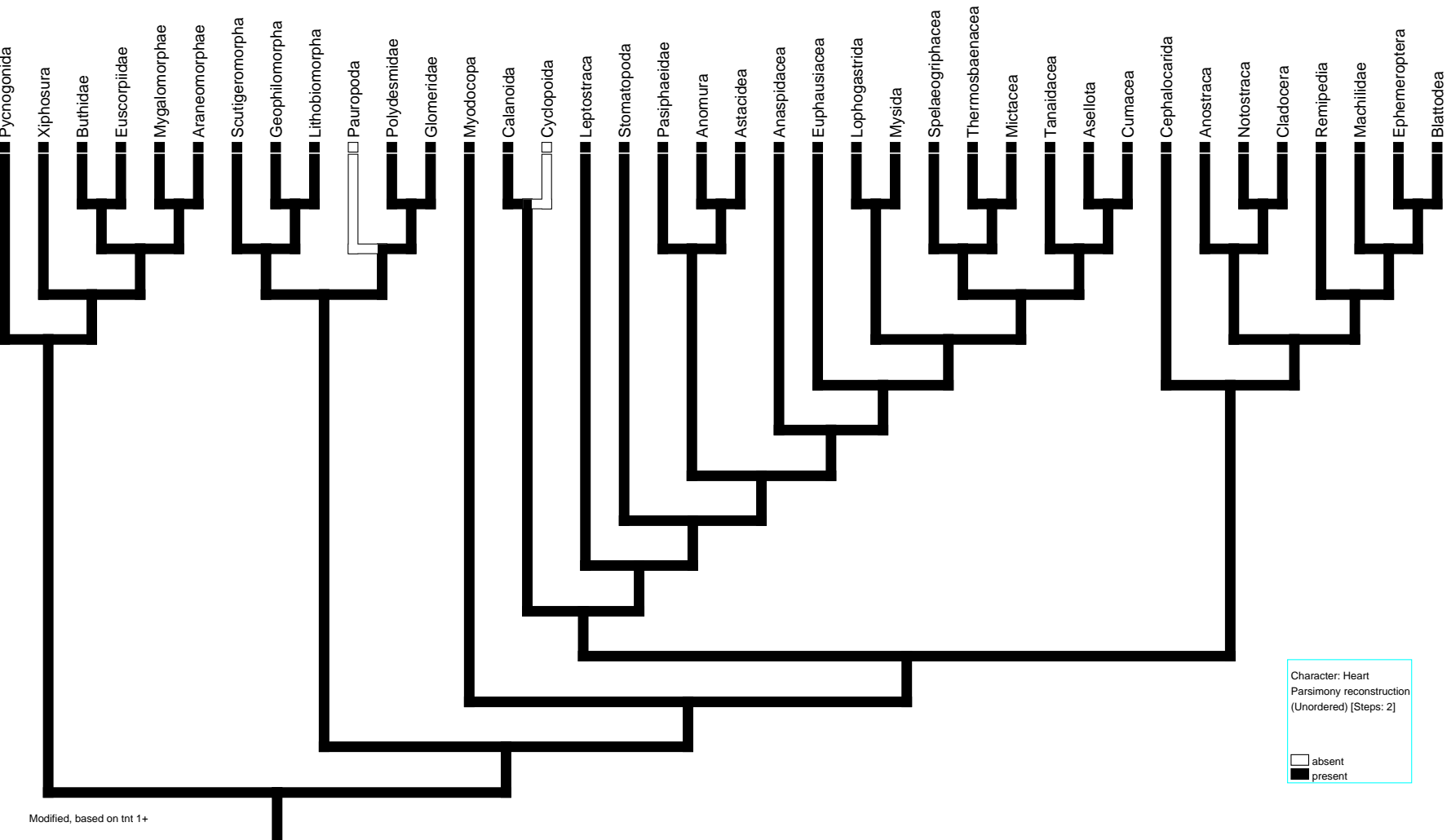

Character: Heart  
Parsimony reconstruction  
(Unordered) [Steps: 2]

☐ absent  
☒ present

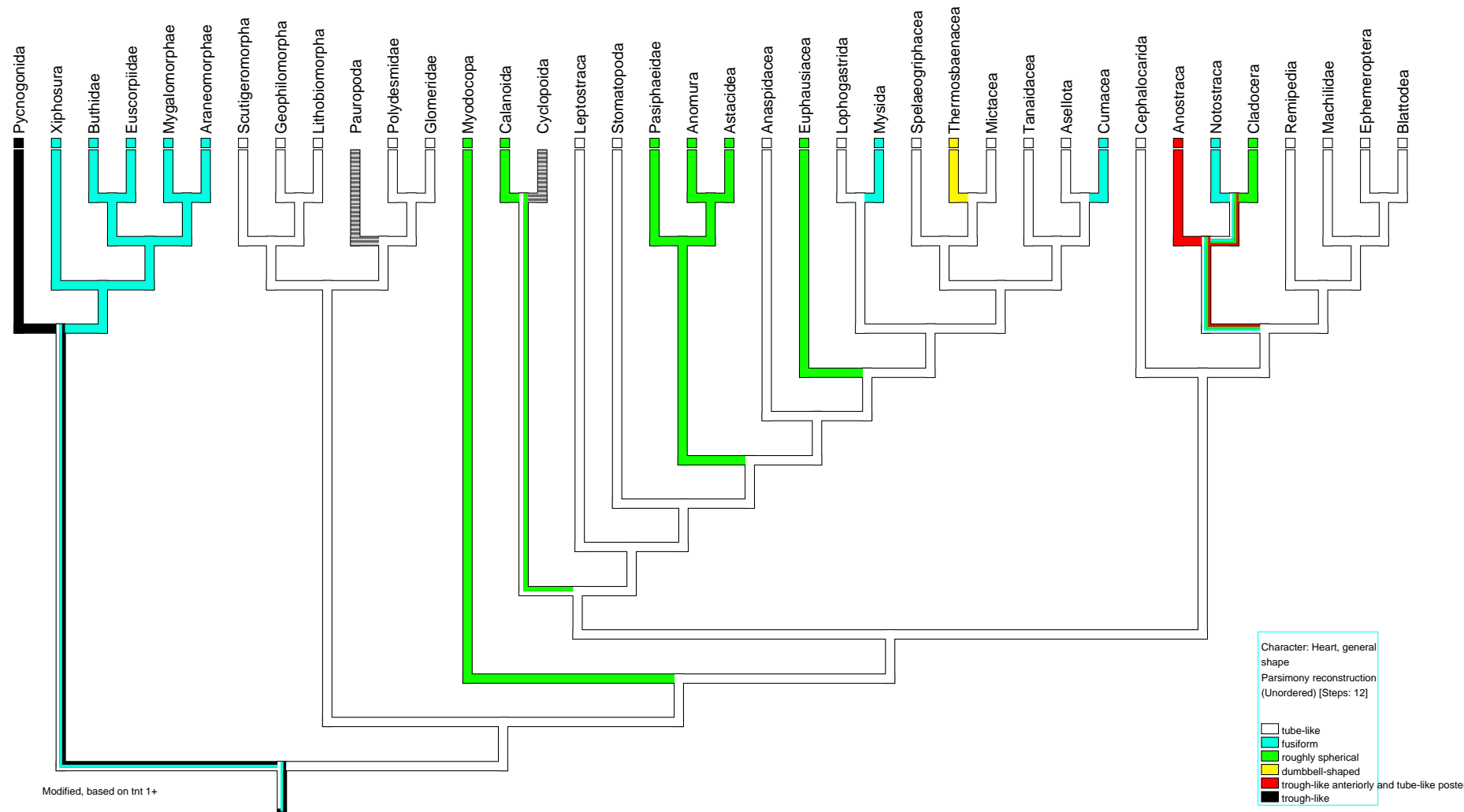

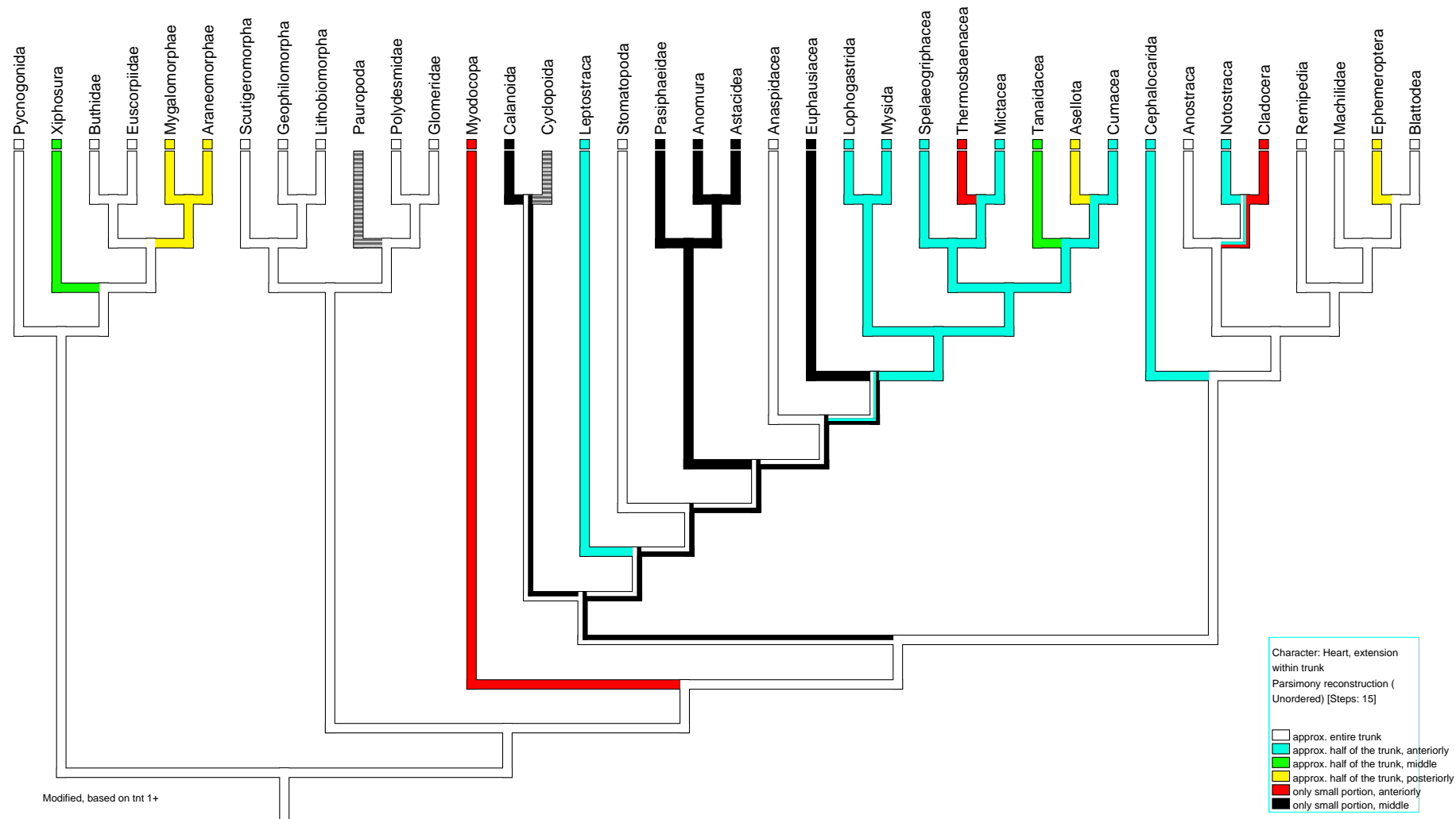

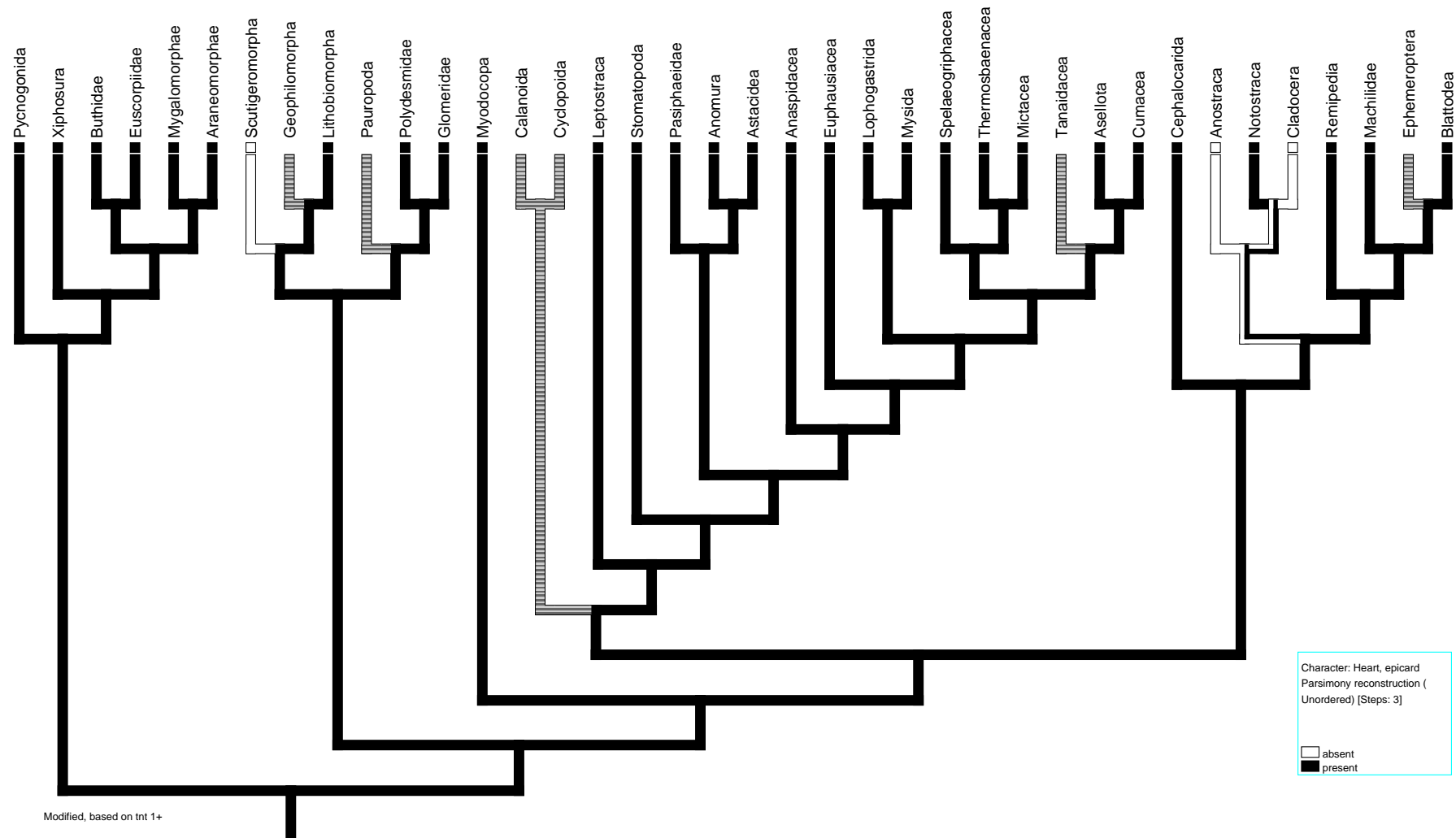

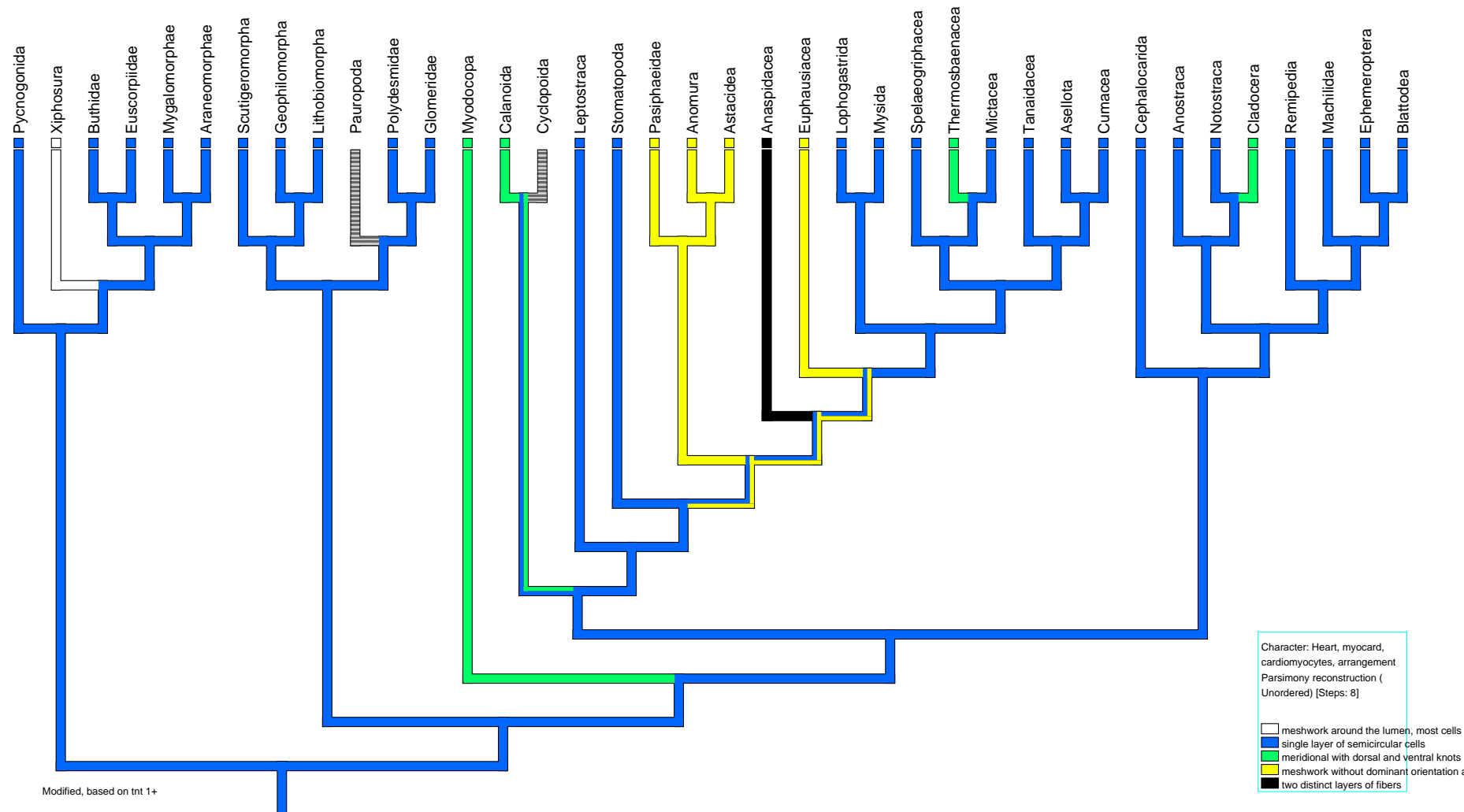

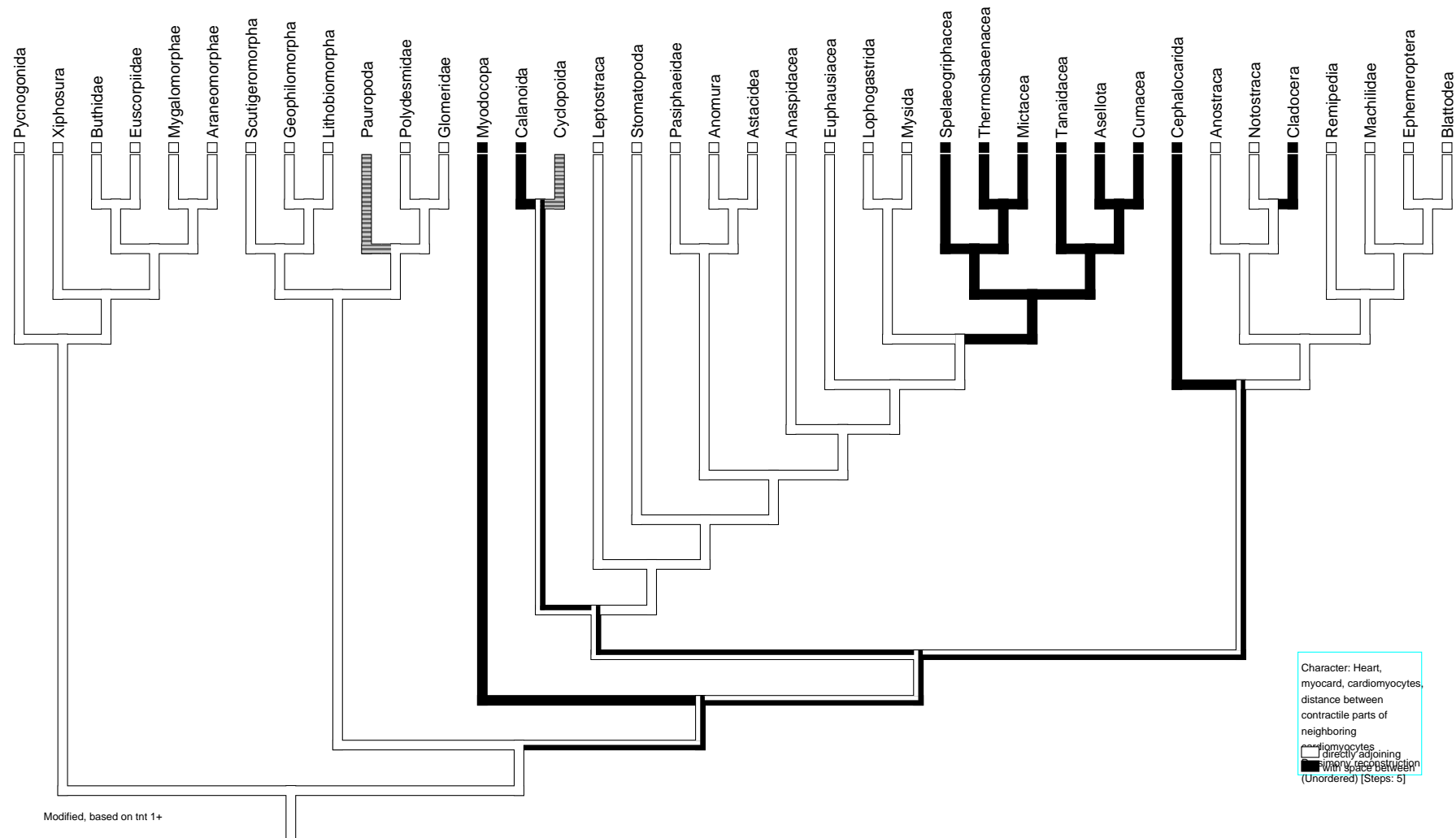

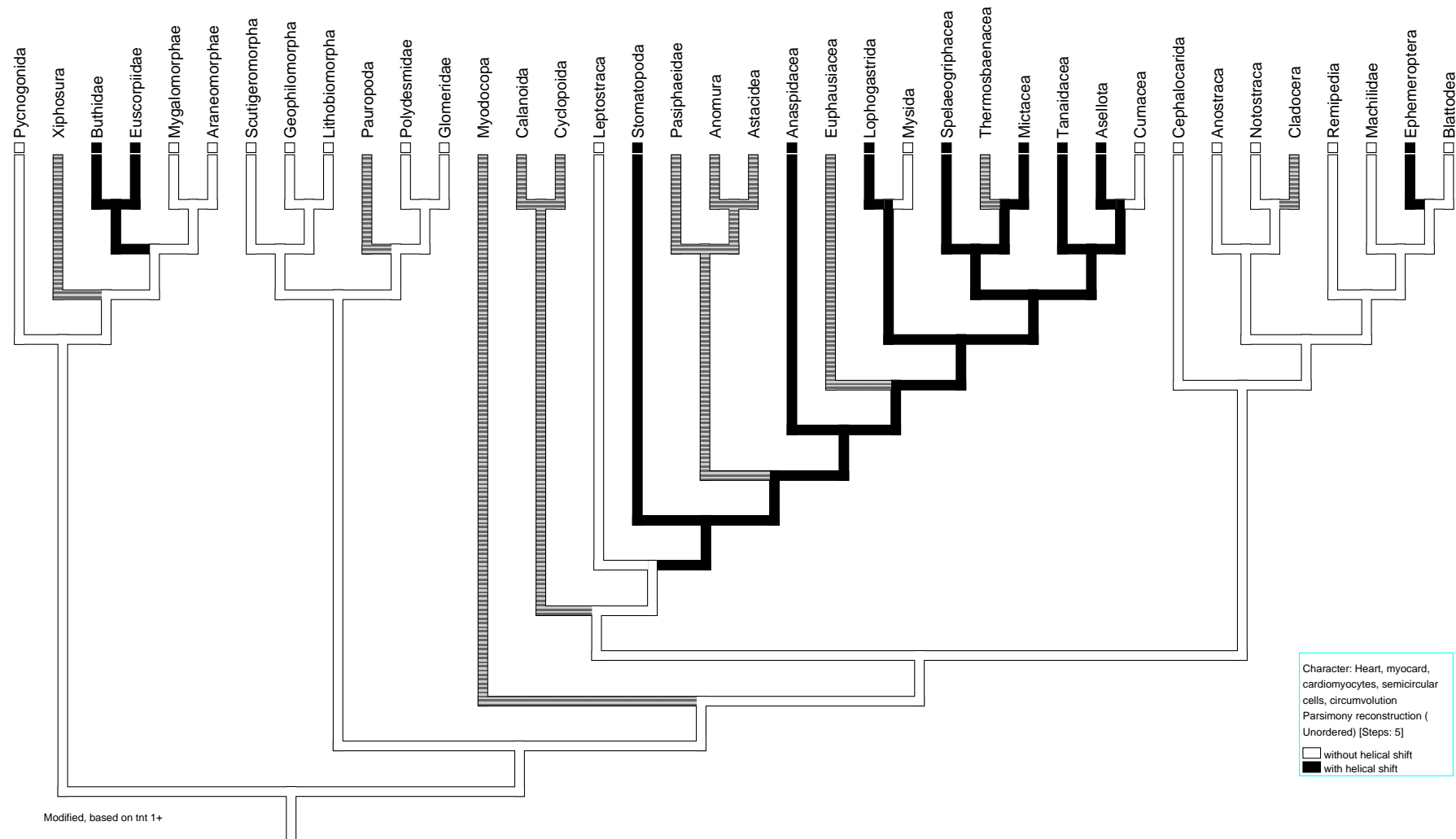

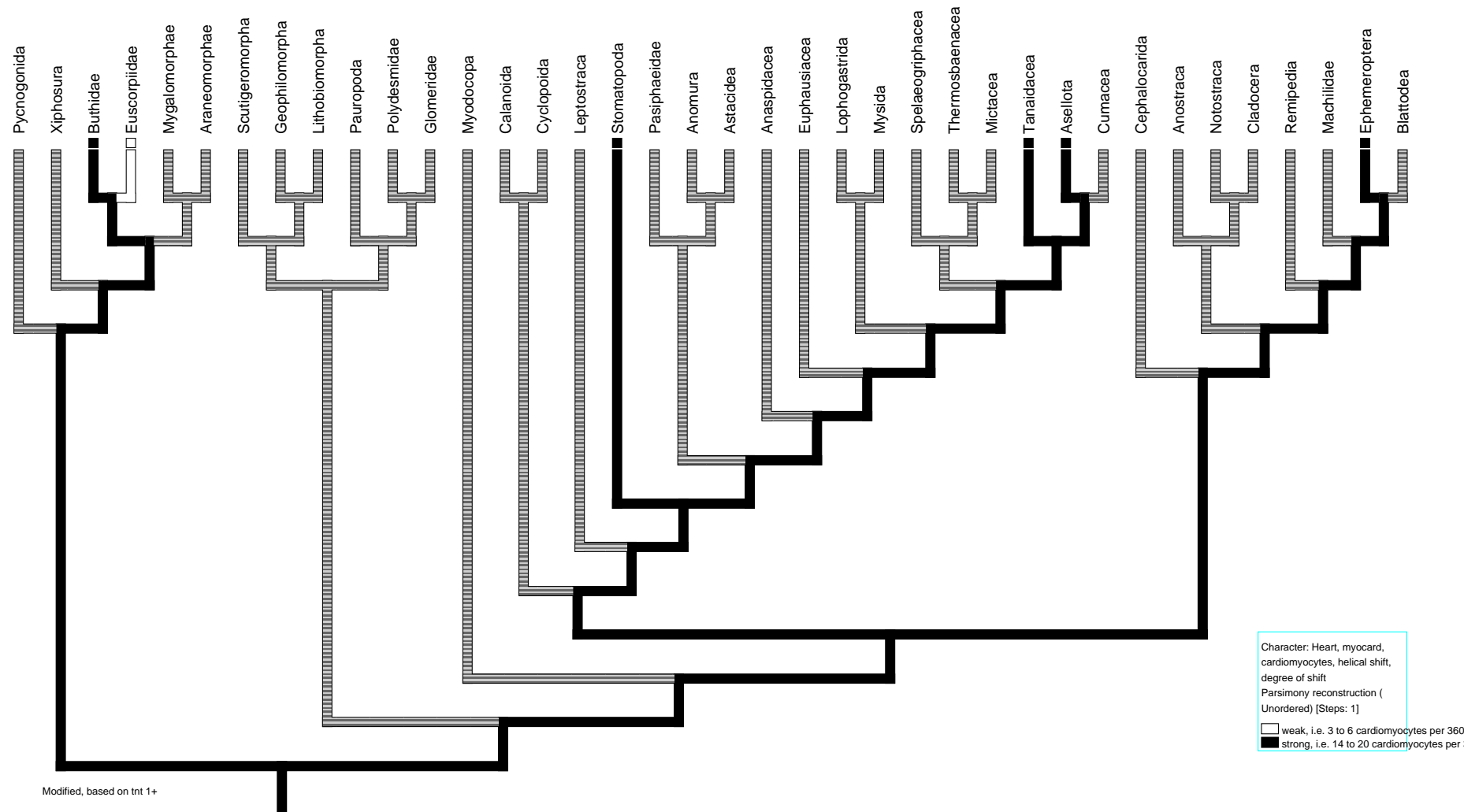

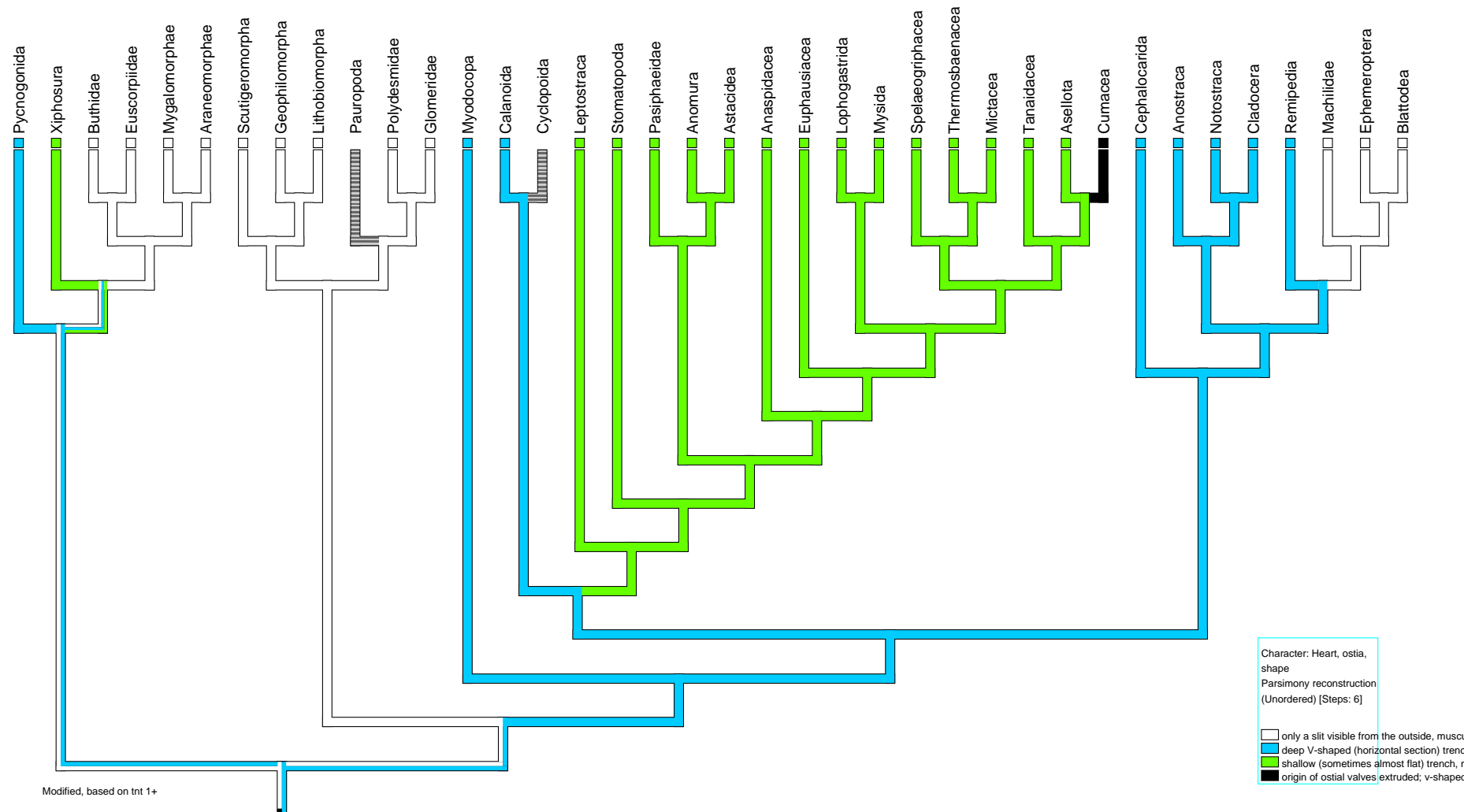

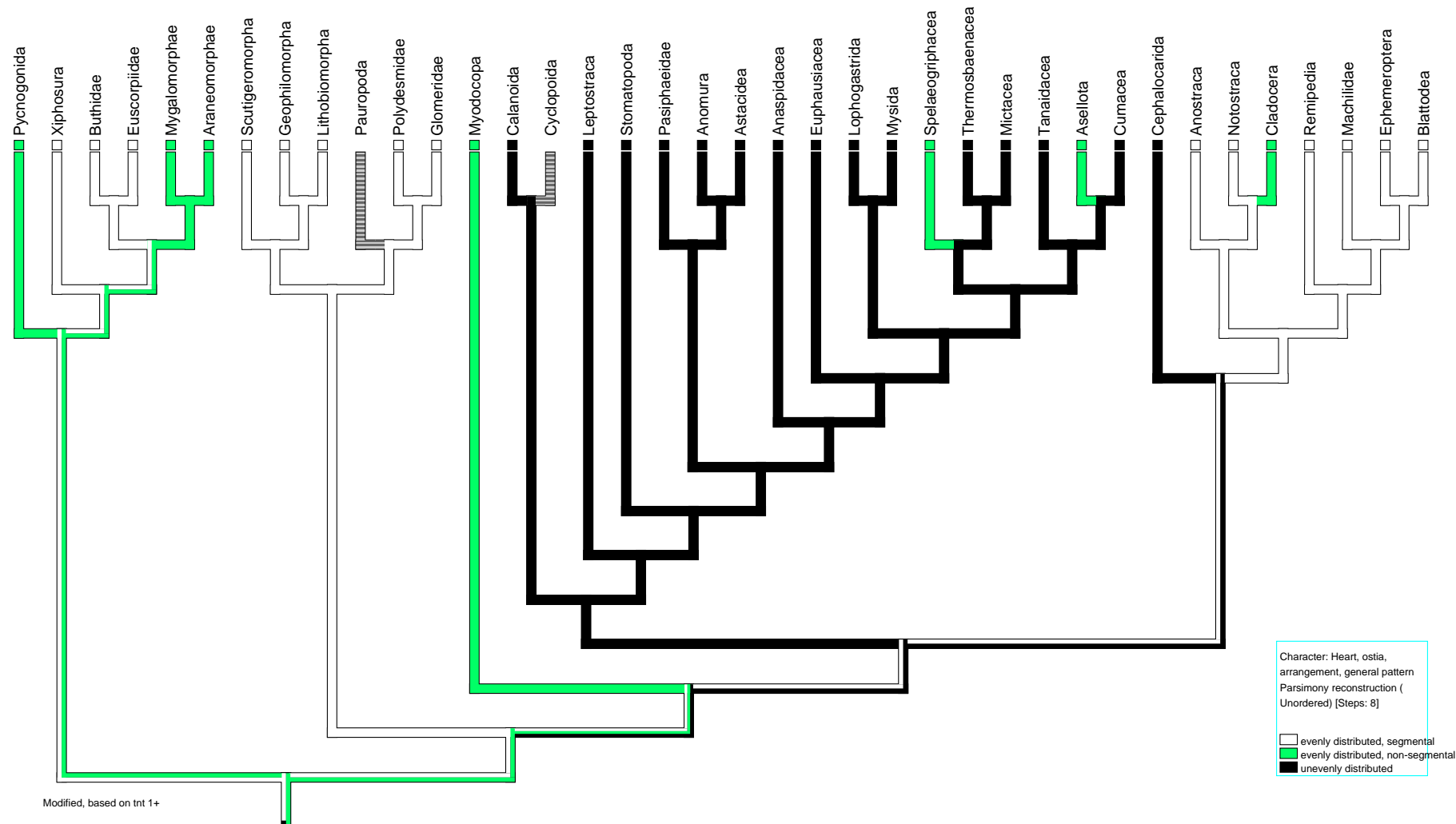

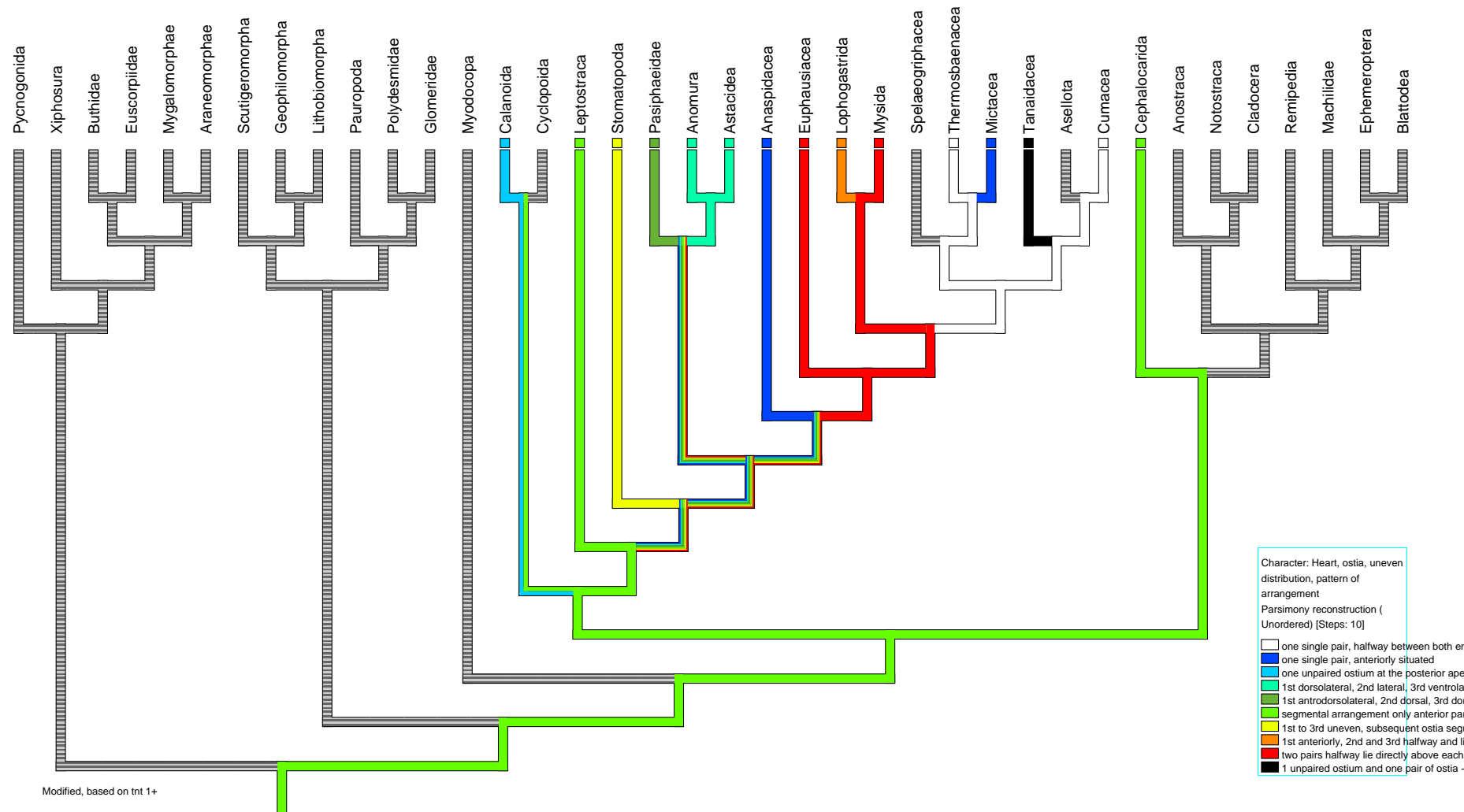

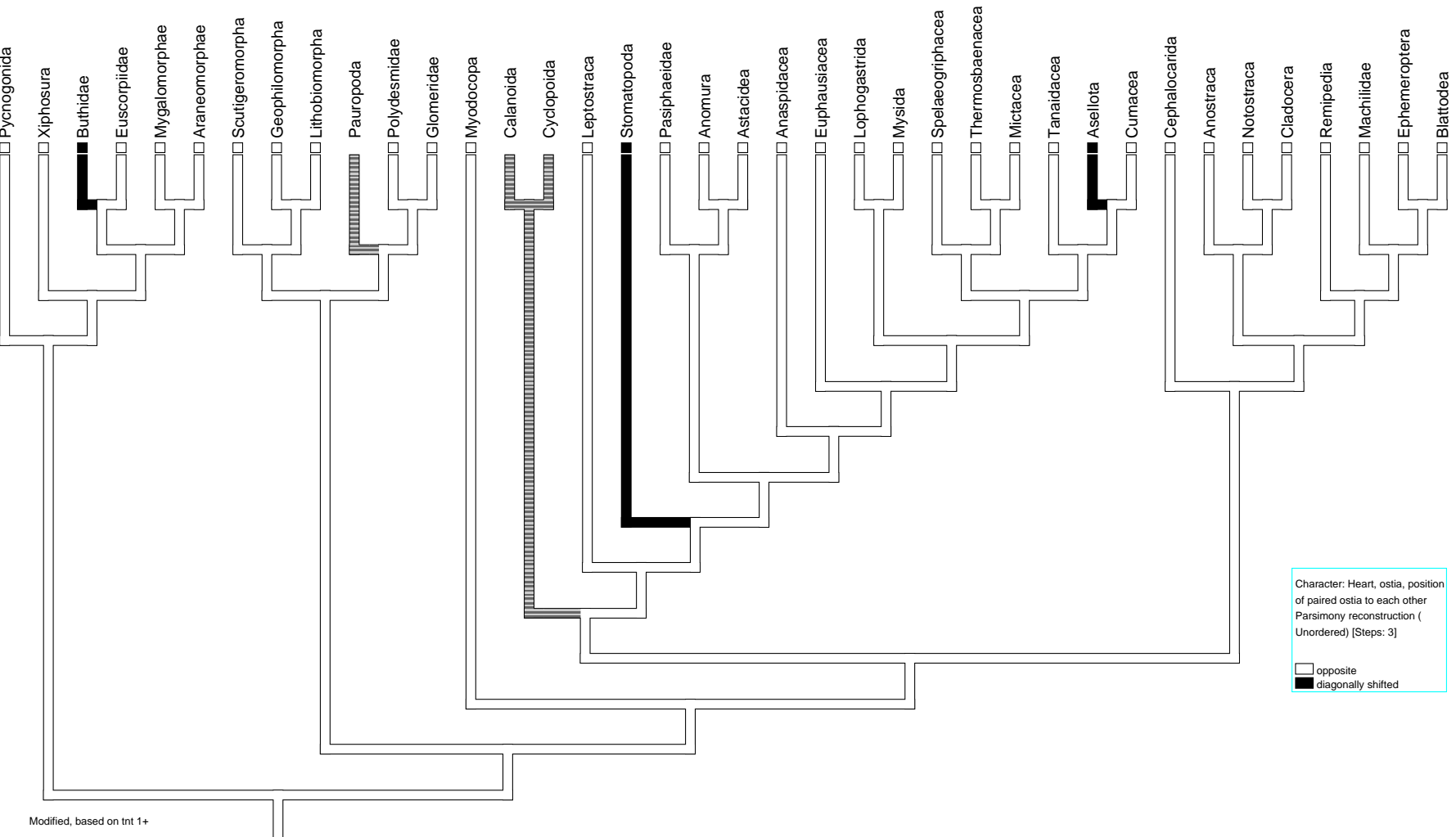

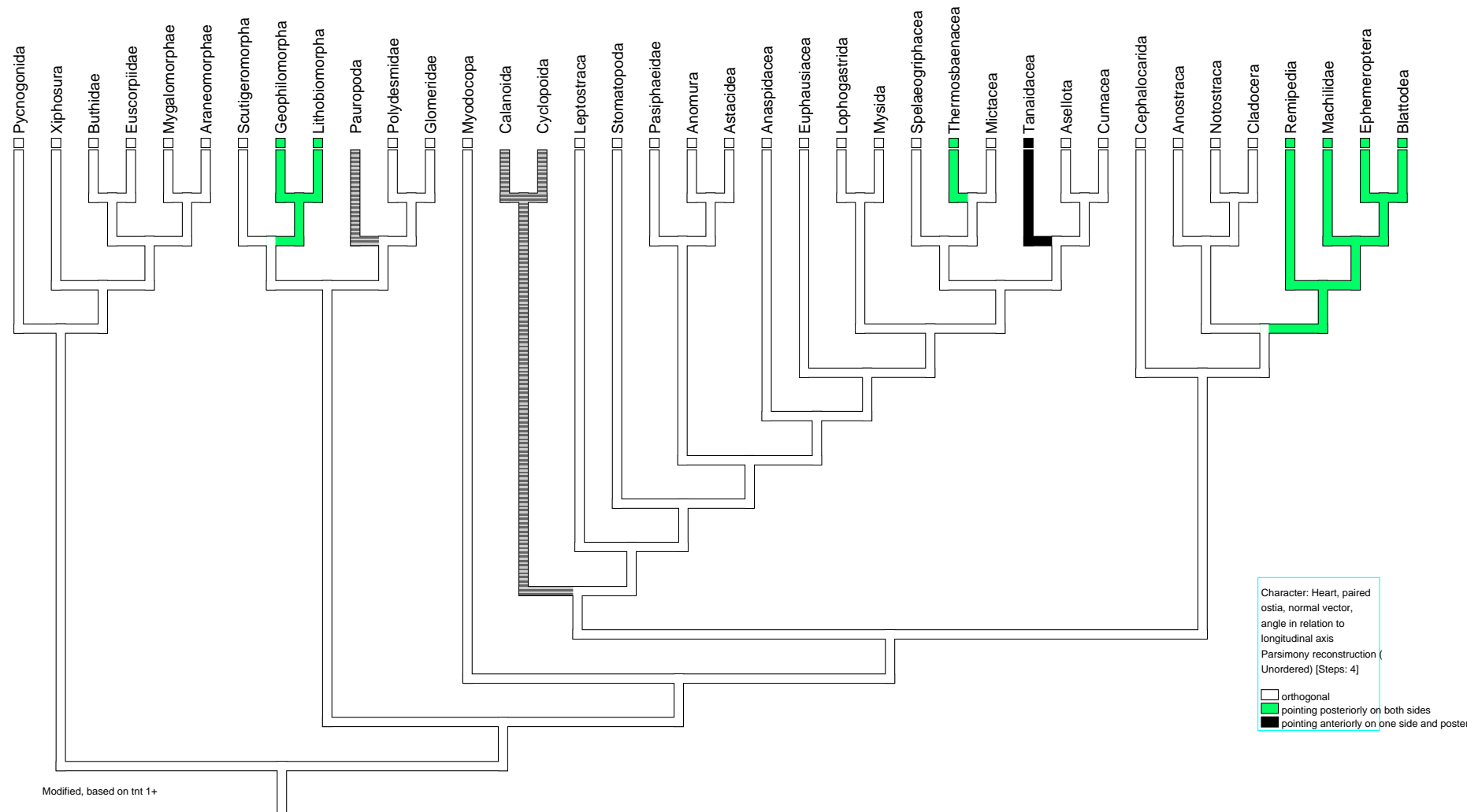

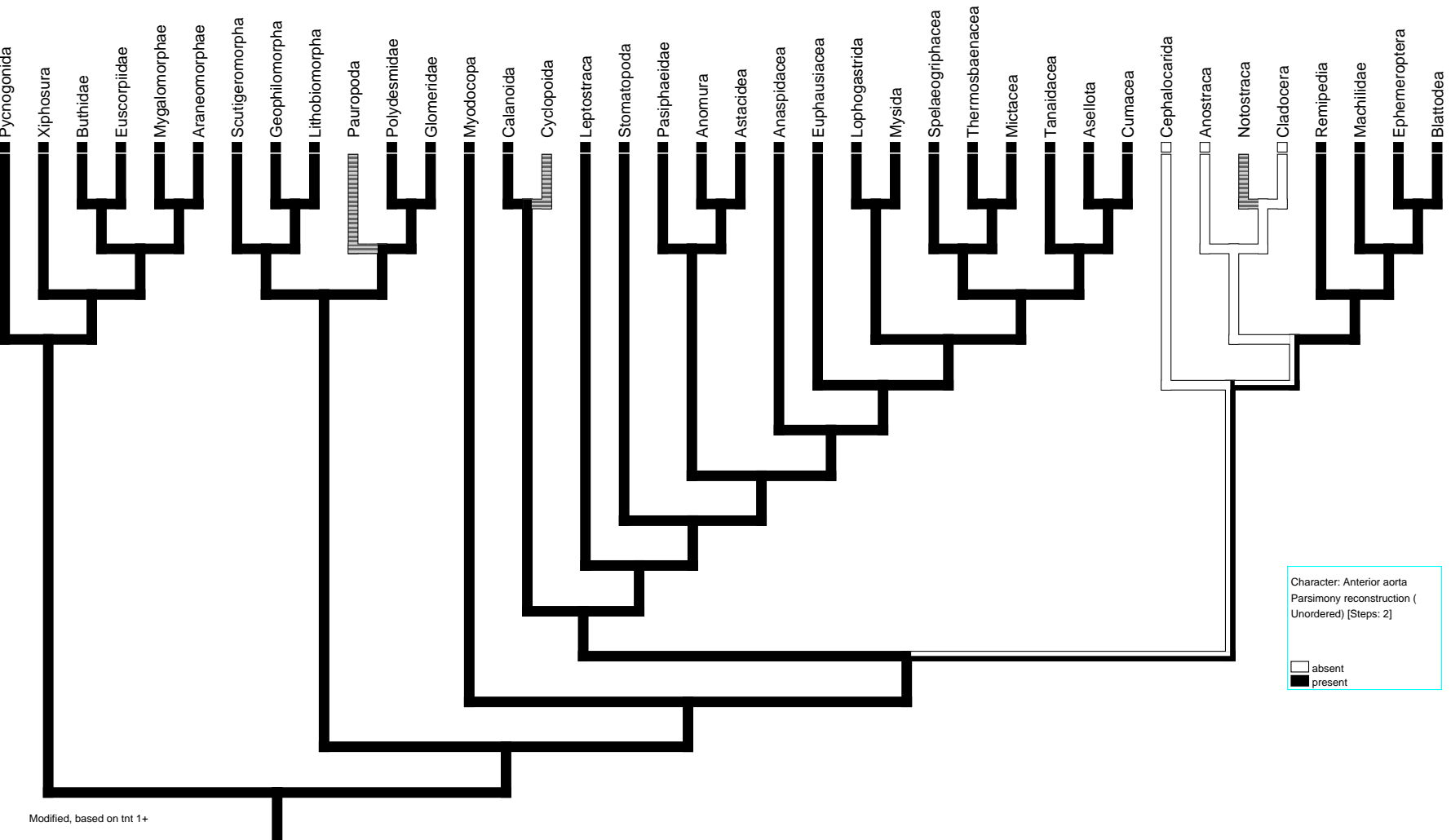

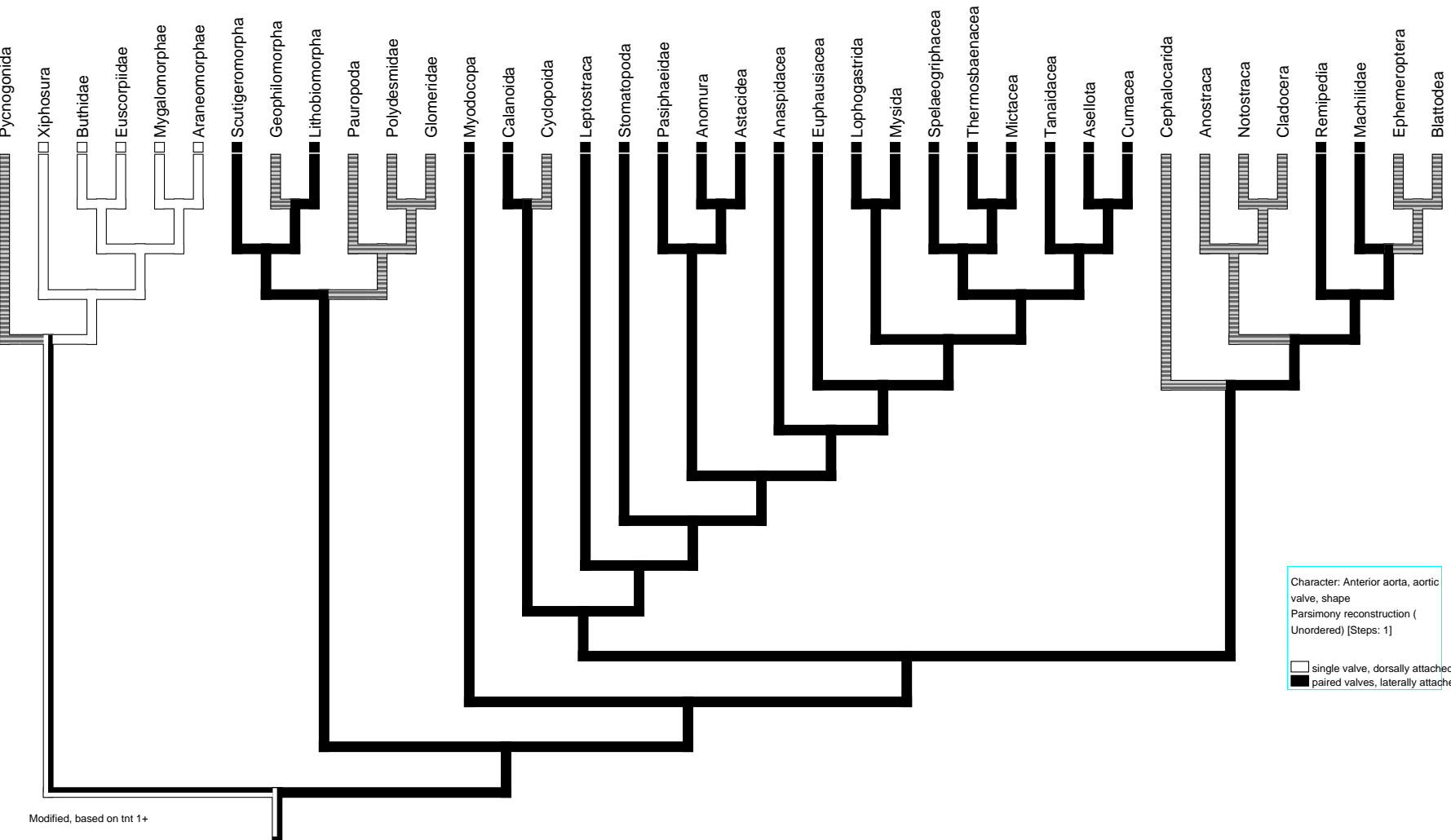

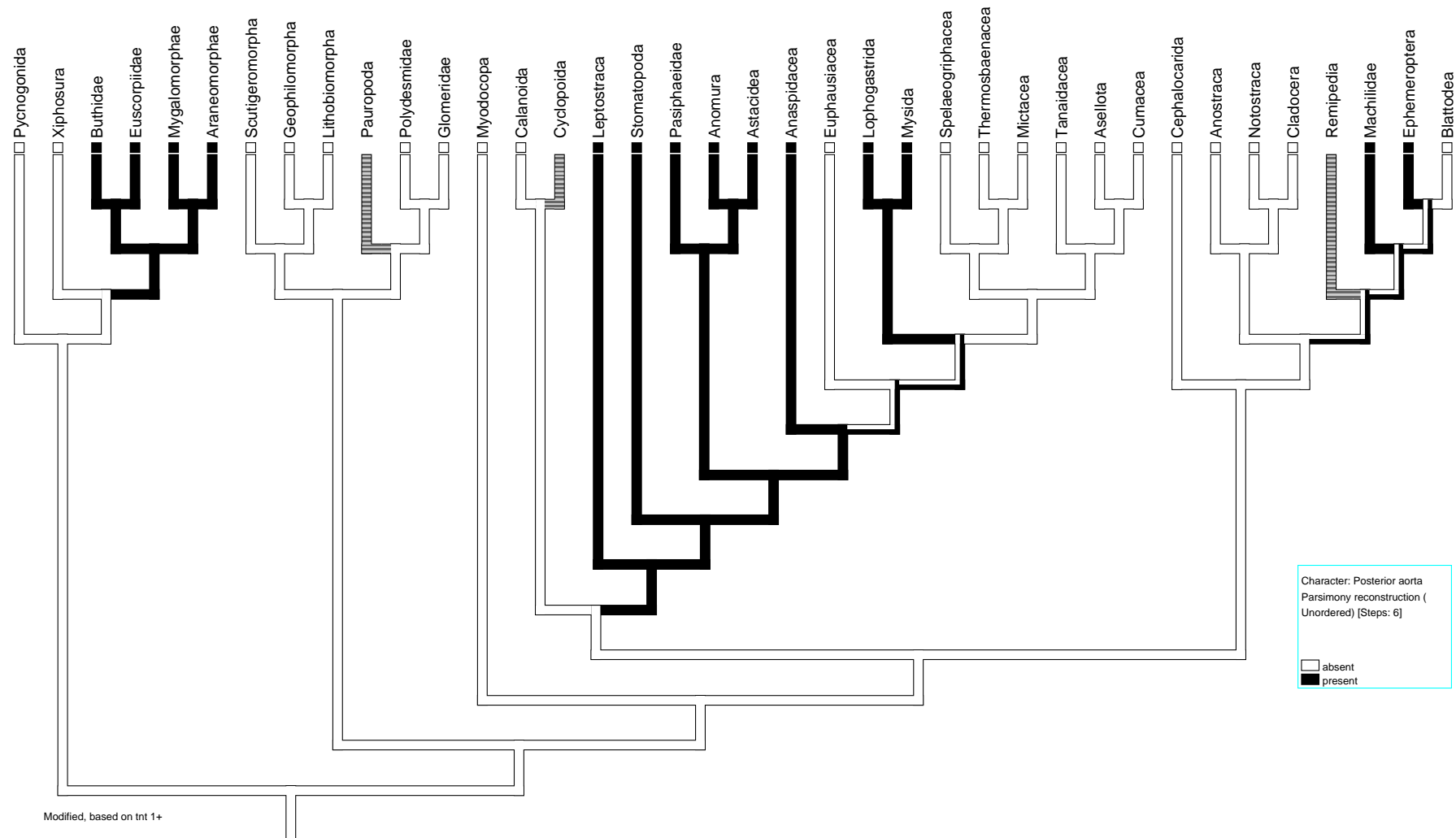

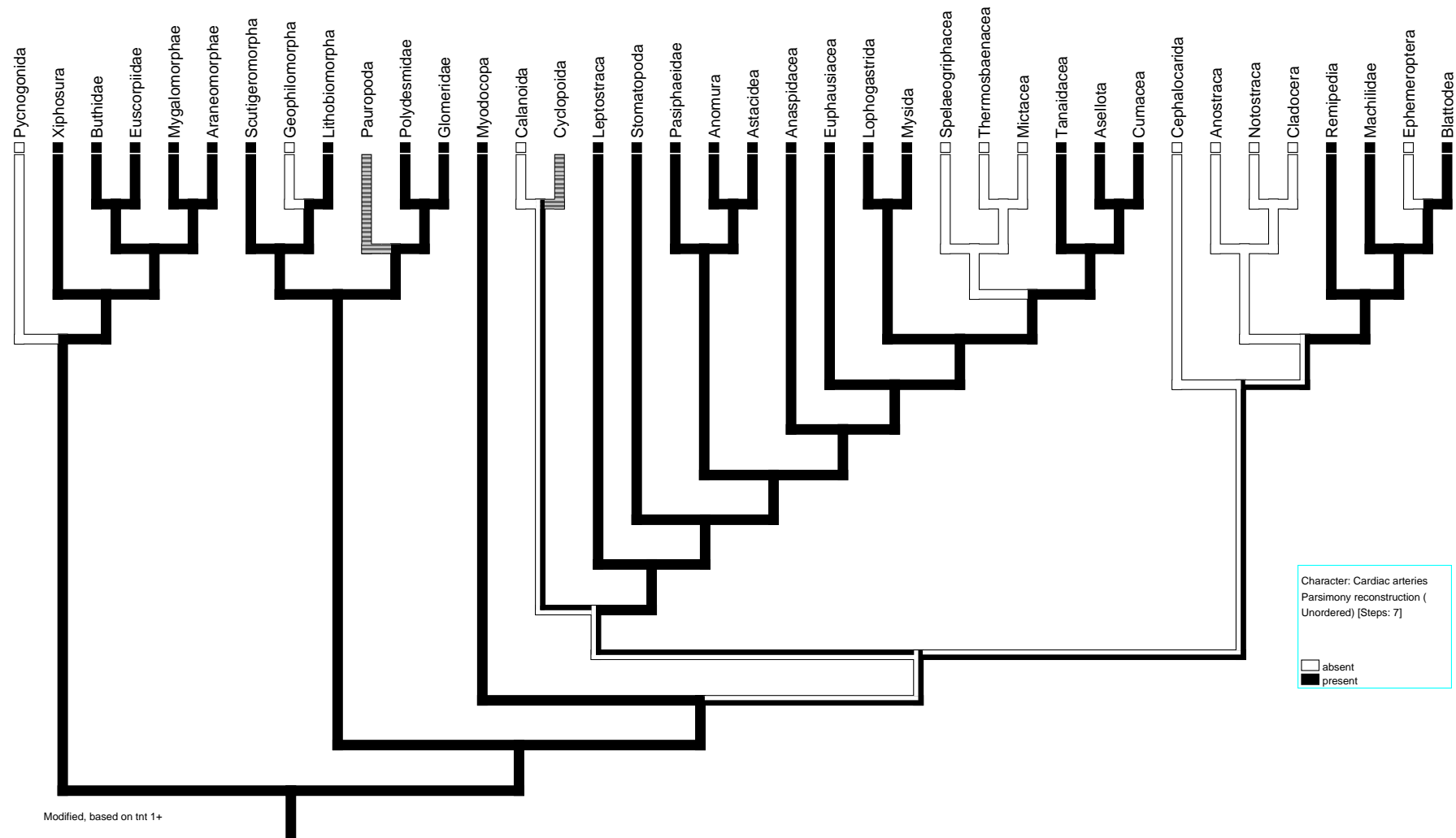

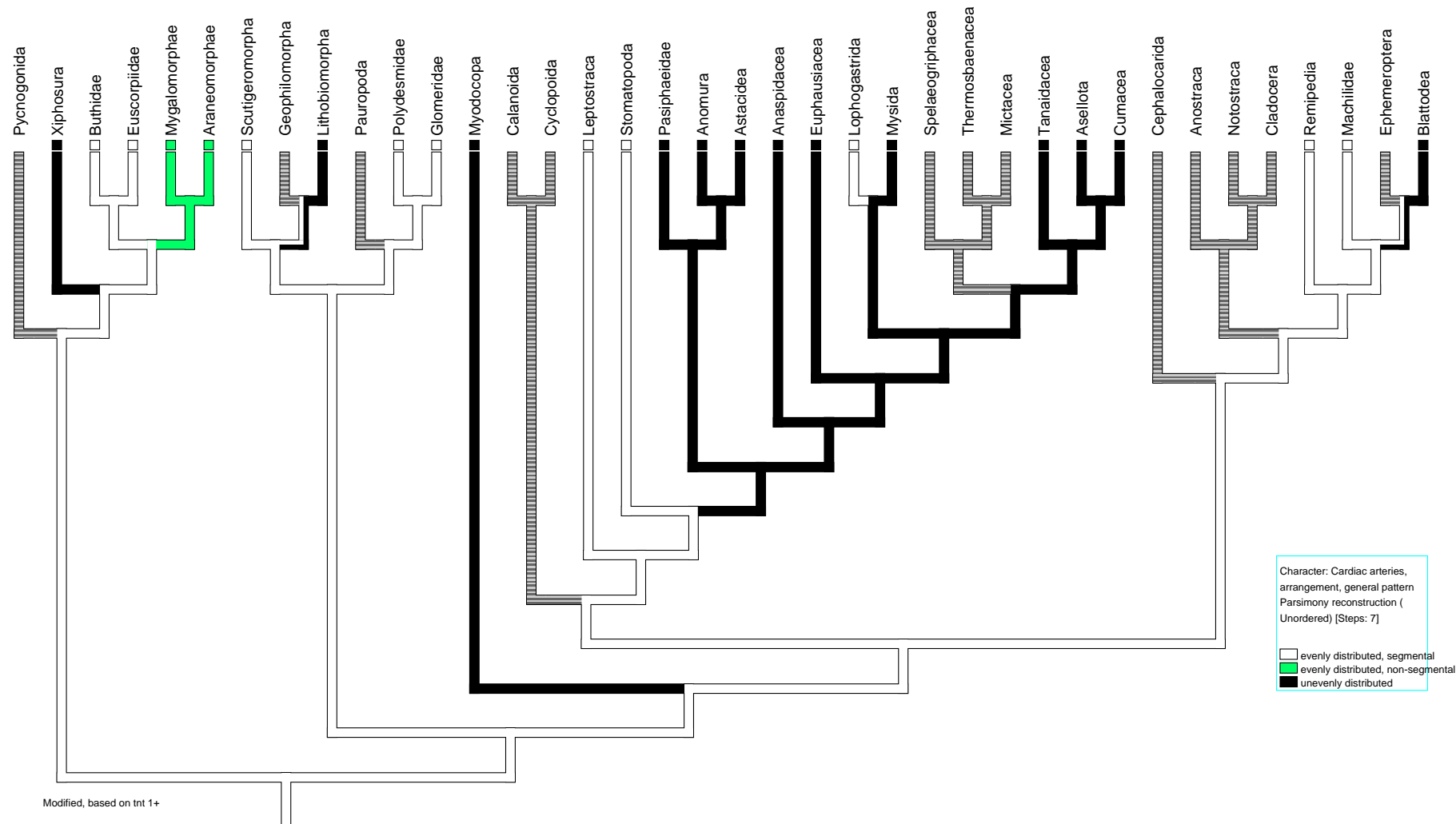

Supplement: S1 File — (PDF) [file pone.0201702.s003.pdf]
